# Identification of human telomerase assembly inhibitors enabled by a novel method to produce hTERT

**DOI:** 10.1093/nar/gkv425

**Published:** 2015-05-09

**Authors:** Guillaume Kellermann, Markus Kaiser, Florent Dingli, Olivier Lahuna, Delphine Naud-Martin, Florence Mahuteau-Betzer, Damarys Loew, Evelyne Ségal-Bendirdjian, Marie-Paule Teulade-Fichou, Sophie Bombard

**Affiliations:** 1 INSERM UMR-S 1007, Cellular Homeostasis and Cancer, Paris, France; 2 Université Paris Descartes, Paris Sorbonne Cité, Paris, France; 3 Institut Curie, CMIB, CNRS UMR 9187- INSERM U1196, Orsay, France; 4 Institut Curie/laboratoire de spectrométrie de masse protéomique, Paris, France; 5 INSERM U-1016, Institut Cochin, Paris, France

## Abstract

Telomerase is the enzyme that maintains the length of telomeres. It is minimally constituted of two components: a core reverse transcriptase protein (hTERT) and an RNA (hTR). Despite its significance as an almost universal cancer target, the understanding of the structure of telomerase and the optimization of specific inhibitors have been hampered by the limited amount of enzyme available. Here, we present a breakthrough method to produce unprecedented amounts of recombinant hTERT and to reconstitute human telomerase with purified components. This system provides a decisive tool to identify regulators of the assembly of this ribonucleoprotein complex. It also enables the large-scale screening of small-molecules capable to interfere with telomerase assembly. Indeed, it has allowed us to identify a compound that inhibits telomerase activity when added prior to the assembly of the enzyme, while it has no effect on an already assembled telomerase. Therefore, the novel system presented here may accelerate the understanding of human telomerase assembly and facilitate the discovery of potent and mechanistically unique inhibitors.

## INTRODUCTION

Telomerase maintains the length of telomeres by catalyzing the elongation of the 3′ end of telomeric DNA. In humans, the core enzyme is composed of two components, a catalytic reverse transcriptase protein (hTERT) and a noncoding RNA (hTR) that provides the template for telomere synthesis (1–3). Both components functionally associate in the nucleus during the S phase, with the transient assistance of several additional factors (3–5). As telomerase is reactivated in 85% of human tumors and supports the unlimited proliferation of cancer cells, it is a promising target for cancer treatment. Indeed, a telomerase inhibitor is expected to provide a therapeutic benefit in most cancers while having little side-effects (6). The adult stem cells that express telomerase in normal tissues divide slowly and have long telomeres, therefore they should be less impacted by telomerase inhibition than the cancer cells which divide rapidly and usually possess short telomeres. In the past decades, several strategies have been proposed to inhibit telomerase, but the present inhibitors lack of specificity and potency *in vivo*. Therefore, there is a need to discover new classes of telomerase inhibitors. Presently, most drugs target the activity of the full enzyme (6). Despite pioneering attempts which showed that telomerase reconstitution can be perturbed *in vitro* by small RNA-binding molecules (7), no specific inhibitor of telomerase assembly has been reported so far, because only low throughput screens can be performed using the current system based on the rabbit reticulocyte lysate (8). Indeed, this complex mixture traps drugs, produces artifacts (9), and necessitates an immunoprecipitation step for the reliable measurement of telomerase activity, rendering the procedure incompatible with large-scale screenings. Alternative attempts have been stopped, due to the impossibility to produce large amount of soluble TERT (10). Indeed, several groups reported their inability to produce recombinant hTERT in bacteria, yeast or insect cells (8,11,12). A lack of solubility of the protein has been repeatedly described in insect cells (13–15). Although small amounts of human telomerase can nevertheless be detected in yeast or insect cell extracts (15–17), recombinant hTERT no longer produced telomerase activity after purification (18–20), precluding its use for the identification of factors capable to regulate telomerase assembly.

Here, we present a method to reconstitute human telomerase with purified hTERT. This system provides a decisive tool to study the proper assemblage of the telomerase ribonucleoprotein complex and also enables the large chemical screening for small-molecules capable to interfere with telomerase assembly.

## MATERIALS AND METHODS

### Production of recombinant hTERT

Constructs using the GAPDH promoter were cloned into the pGAPZ vector, whereas constructs using the AOX1 promoter were cloned into the pPIC 3.5K vector (Life Technologies). The expression was followed by western blot analysis using antibodies against GST (Sigma), HA (Covance, HA.11,) or hTERT (rabbit monoclonal Epitomics [Y182], Abcam 32020) (21). Soluble protein fractions were prepared by the centrifugation of the samples at 10 000 rpm for 30 min. The pGAPZ-MBP-hTERT vector was obtained by gene synthesis (Eurofins Genomics) after optimization of the coding and untranslated regions (Supplementary Figures S1 and S2). Twenty micrograms of plasmid was linearized with AvrII, purified and electroporated into the X-33 strain of *P. pastoris* (Life Technologies) using a Bio-Rad Gene Pulser (1500 V, 25 μF, 200 Ω) to generate stable transformants. Multi-copy integrants were selected on agar plates (0.2% yeast nitrogen base with ammonium sulfate, 1% yeast extract, 2% peptone, 2% dextrose, 1 M sorbitol, pH 7.0, 300 μg/ml zeocin, 1.5% agar) and incubated at 27°C for 2–3 days. A colony was re-streaked, amplified in 200 ml (1% yeast extract, pH 7.0, 1% dextrose) at 160 rpm, 29°C, then aliquoted in 2 ml tubes and stored at −80°C with 10% glycerol. For each new culture, yeast were first allowed to recover from freezing 1–2 days on agar plates (0.2% yeast nitrogen base with ammonium sulfate, 1% yeast extract, 2% dextrose, 1.5% agar). Then, they were grown overnight at 160 RPM, 29°C, in 2 l shake-flasks containing 500 ml of medium (2% yeast extract, 4% glucose, 100 mM, monosodium phosphate pH 7.5) until an OD_600_ of 12–15 was reached. The purification was performed in a cold room with cold solutions and refrigerated instruments. Yeast from a 1-l culture were pelleted at 1500 rpm for 10 min, washed in water, then resuspended in 10 ml of water, and added to 10 ml of glass beads (425–600 μm, Sigma) in a 50 ml centrifuge tube (Falcon). Protein extraction was induced by vortexing at maximum speed (3000 rpm) for 10 min. Due to the culture conditions defined above, the intracellular pH measured in the whole cell lysate was close to 6.3. This parameter was selected because it ensures that hTERT was not degraded, and was also found to provide the best yield for the subsequent purification of this protein (Supplementary Figure S3A). The optimal pH for the recovery of active hTERT was different from the optimal pH of telomerase for enzymatic activity *in vitro* (Supplementary Figure S3B). Then, the lysate was centrifugated at 3000 g for 10 min and again at 3000 g for 15 min in a new tube. Salt concentration was raised to 50 mM with a 5 M NaCl stock solution and 100 μg RNase A was added (Fermentas). The supernatant was applied to 1 ml of pre-rinsed amylose-agarose beads (NEB) in a 15 ml polypropylene column (Qiagen). After 1 h on a rotating wheel, the column was washed once with a high-salt solution (600 mM NaCl, 10 mM monosodium phosphate pH 7.0), and twice with a salt-free solution (10 mM Hepes pH 7.0). MBP-hTERT was eluted with 1 ml elution buffer (130 mM KCl or NaCl, 2 mM MgCl_2_, 10 mM Hepes pH 7.0, 50 mM maltose, 0.2 units/μl RiboLock, Fermentas). MBP-hTERT protein concentration was estimated on SDS-PAGE after Coomassie brilliant blue staining against bovine serum albumin (BSA) dilutions. MBP-hTERT was typically obtained in a concentration between 0.1 and 0.3 mg/ml. The protein was stable for at least 2 days at 4°C, and for several months when stored frozen (−80°C) with the addition of 10% glycerol. The protein was concentrated using Vivaspin or Vivacell concentrators (PES membrane, Sartorius). To cleave the MBP, an excess of His6-TEV protease was added and the sample was incubated for one hour at room temperature.

### hTR synthesis and purification

pT7hTER plasmid (kind gift of Joachim Lingner, EPFL) or two pIDT plasmids containing the complementary hTR truncations (33–209 and 207–325) under the T7 promoter were linearized using BamHI or EcorI and purified by phenol/chloroform followed by ethanol precipitation. Five microgram of DNA was used as a template in 40 mM Tris–HCl pH 7.9, 8 mM MgCl_2_, 10 mM dithiothreitol, 2 mM spermidine, 4 mM NTPs and 250 U of T7 RNA polymerase (New England Biolabs) in a total volume of 100 μl for 3 h at 37°C. The sample was then heated for 2 min at 70°C and stored at −20°C. Truncations or full length hTR used for drug screening were not purified. For biochemical studies, hTR was purified using an oligo-displacement method (22). EDTA was added to a final concentration of 10 mM and hTR was diluted 3 times with solution A (333 mM KCl, 1 mM EDTA, 10 mM Hepes pH 7.0). One nanomole of affinity oligonucleotide [5′-biotin-CTAGACCTGTCATCA-rmeG-(rmeU)_2_-rmeA-(rm-eG)_3_-(rmeU)_2_-rmeA-rmeG (rme  =  2′*O*-methyl ribonucleotides)] was added and the sample was incubated at 30°C for 10 min. Agarose–streptavidin beads were then added and the sample was agitated for 1 h at 4°C. Then, beads were washed three times with solution A, and hTR was eluted for 30 min at room temperature with a three molar excess of displacement oligonucleotide 5′-GATTGGGATTCTGATGACAGGTCTAG-3′ with one bead volume in 130 mM KCl, 2 mM MgCl_2_, 10 mM Hepes pH 7.0. hTR concentration was quantified on a 1% agarose gel containing SYBR Green II RNA (Invitrogen) against a reference RNA using Storm 860 molecular imager and the Imagequant software (GE Healthcare).

### Electrophoretic mobility shift assay

hTR was synthetized with the addition of 50 μCi of [α-^32^P]-CTP and purified as above. For each lane, 1 μg of MBP-hTERT was incubated for 1 h with 0.5 μg of labeled hTR in 20 μl. Complexes were migrated at 110 V for 2 h on a 1.2% refrigerated agarose gel in 1× TBE. The gel was fixed for one hour in 10% acetic acid and 10% ethanol, dried and exposed to a phosphorimager screen. STORM 860 (GE Healthcare) was used to perform the scan and Imagequant software to quantify the images.

### Telomerase reconstitution and inhibition

MBP-hTERT (100–300 ng/μl) was mixed with an equal amount of hTR and incubated at room temperature for 45 min, then placed at 37°C for 10 min to produce recombinant telomerase typically between 0.3–1 μM. For maximal inhibition of telomerase activity by TAI1, MBP-hTERT was preincubated 10 min with the compound before the addition of hTR.

### Direct telomerase assay

The direct assay was performed as described (23), with slight modifications. For each sample to be analyzed, recombinant telomerase (300 nM) was reconstituted in the presence of absence of the inhibitor in a volume of 12 μl and then assessed for telomerase activity in a reaction buffer containing 40 mM Tris–HCl pH 7.9, 1 mM MgCl_2_, 1 mM dithiothreitol, 2 mM spermidine, 20 mM KCl, 40 μM dATP, 80 μM dTTP, 2 μM dGTP, 20 μCi of [α-^32^P]-dGTP (3000 Ci/mmol) and 2 μM of a 5′-biotinylated primer as a telomerase substrate. Reactions were stopped by adding 25 mM EDTA and small amounts of a 5′-radiolabeled and 3′-biotinylated 15-mer non-telomeric oligonucleotide (5′-CCAGTCATCTAGATC-3′) were added to each reaction as a recovery control (RC). Unincorporated nucleotides were removed by binding the biotinylated primer to 20 μl of streptavidin-agarose beads (GE Healthcare) for 30 min at room temperature. Beads were washed once with 10 mM Tris–HCl pH 8.0, 1 mM EDTA, 1 M NaCl, and twice with 10 mM Tris–HCl pH 8.0 and 1 mM EDTA. The primer was eluted by heating at 95°C for 10 min in 90% formamide, 10 mM EDTA, 0.5 mM biotin (Sigma) and separated on 20% polyacrylamide–urea sequencing gels (19:1 acrylamide:bisacrylamide ratio). The gel was covered with a plastic film, exposed to a phosphorimager screen and scanned using a STORM 860 molecular imager (GE Healthcare). Enzyme processivity was measured using the Imagequant software (GE Healthcare) and was calculcated for each lane following the formula: 100 × (1 − (intensity of repeat 1)/(intensity of repeat 1+2+3+4))).

### RT-TRAP

RT-TRAP was performed as described (24), with slight modifications. For drug screening, RT-TRAP was performed in 96-well plates using an Applied Biosystems 7900HT. Then, to achieve accurate measurement of telomerase activity, RT-TRAP was performed in capillaries using a LightCycler^®^ 2.0 instrument (Roche). Reactions were carried out in a 20 μl scale with SYBR Green PCR Master Mix (Applied) or LightCycler^®^ FastStart DNA Master SYBR Green I (Roche) with 0.1 μg of telomerase primer TS (5′-AATCCGTCGAGCAGAGTT-3′), 0.05 μg of reverse primer ACX (5′-GCGCGGCTTACCCTTACCCTTACCCTAACC-3′), 0.5 mM additional MgCl_2_, and 1 μl of the sample to be tested. When using the Applied Biosystems instrument, samples were incubated for 30 min at 37°C, 10 min at 95°C and amplified in 40 PCR cycles for 30 s at 95°C, 60 s at 60°C and 60 s at 72°C. When using the LightCycler^®^, samples were incubated 30 min at 37°C, 10 min at 95°C and amplified in 40 PCR cycles for 60 s at 95°C and 5 s at 60°C. PCR efficiency was calculated by a serial dilution of the most active sample and crossing-points (CP) were determined using the second derivative maximum method (LightCycler^®^ software). For each experiment, the most active sample was arbitrarily set to 100 and values are presented as Telomerase arbitrary units (T.A.U.).

### Chemical library screening

The Institut Curie–CNRS chemical library contains 9200 molecules stored in 96-well microplates at a concentration of 10 mg/ml in dimethyl sulfoxide (DMSO). Microplates were kept at −20°C. Three thousand compounds of this collection were screened at 3 μg/ml. The residual level of DMSO (<0.1%) had no effect on the assay. For each test, 100 ng of hTERT, the molecule diluted in 130 mM KCl, 1 mM MgCl_2_, Hepes 10 mM pH7.0, and 50 ng hTR were sequently distributed in 96-well PCR plates. After 45 min at room temperature, the PCR mix was added and telomerase activity was measured by RT-TRAP. Positive and negative control wells were included in each assay (*Z*-factor was 0.3).

### Chemical compounds

The inhibitor TAI1 (C_45_H_61_N_9_O_10;_ MW 888.02) was synthesized *via* reductive amination from 2-carboxaldehyde dibenzo[*b*,*j*][4,7]phenanthroline (synthesis described in (25)) and a protected tobramycin–lysine analogue (synthesis described in (26)) following the procedure previously described (26) and summarized in (Supplementary Figure S4). In brief, 2-carboxaldehyde dibenzo[*b*,*j*][4,7]phenanthroline (1 eq.) was dissolved in DCM/MeOH (1:1). The protected lysine-tobramycin analogue (2 eq.) and TEA (2 eq.) were added and the resulting mixture was stirred for 4 days at room temperature. The solution was filtered through a pad of celite, evaporated to dryness, taken up in DCM/MeOH (1:1) and NaBH_4_ (3 eq.) was added. The resulting mixture was stirred for 3 h at room temperature and then quenched by addition of a 5% aq. NaHCO_3_ solution. Further DCM was added, the organic phase was separated, dried over Na_2_SO_4_ and the organic phase was evaporated to dryness. The residue was taken up in TFA/DCM (95:5), stirred for 1 h at room temperature and evaporated to dryness. Subsequent RP-HPLC chromatography then delivered purified TAI1. Similar inhibition of telomerase was obtained using two independent batches.

^1^H-NMR (D_2_O, 400 MHz): *δ* = 10.10 (s, 1H, Ar), 9.99 (s, 1H, Ar), 8.42–8.36 (m, 4H, Ar), 8.25–8.06 (m, 4H, Ar), 7.96–7.92 (m, 1H, Ar), 5.50 (d, *J* = 3.4 Hz, H1’), 5.06 (d, *J* = 3.6 Hz, H1’’), 4.49 (s, 2H, NHC*H*_2_Ar), 3.98–3.36 (m, 17H, Lys-H_α_ and remaining sugar protons), 3.16 (t, *J* = 7.8 Hz, 2H, Lys-H_ϵ_), 2.52–2.49 (m, 1H, H2_a_), 2.29–2.42 (m, 1H, H3’_a_), 1.91–1.63 (m, 6H, Lys-H_β_, Lys-H_δ_, H3’_e_ and H2’_e_), 1.43–1.33 (m, 2H, Lys-H_γ_) ppm.

^13^C-NMR (D_2_O, 700 MHz): *δ* = 170.1, 143.0, 141.8, 141.0, 140.7, 139.1, 138.9, 137.2, 135.8, 132.8, 132.2, 130.9, 130.0, 129.6, 128.8, 126.9, 126.6, 124.2, 122.1, 121.8, 120.5, 100.6, 95.0, 83.6, 78.7, 73.8, 73.2, 72.9, 68.0, 65.3, 63.6, 59.7, 54.9, 53.0, 50.4, 49.6, 48.4, 47.8, 47.2, 39.1, 30.5, 29.2, 27.7, 25.2, 21.5 ppm.

ESI–LC–MS: *t*_R_ = 1.67 min, *m/z* = 888.46 calcd. for C_45_H_62_N_9_O_10_^+^ [M+H]^+^ and 444.74 calcd. for C_45_H_63_N_9_O_10_^+^ [M+2H]^2+^, found: 888.27 and 444.87.

The synthesis of the related molecules TD1, TM1 and 11, as well as MMQ1 and the Neomycin bis-lysine acridine macrocycles have been previously described (26–28). Tobramycin and Kanamycin were ordered from Sigma-Aldrich.

### Proteomics and mass spectrometry analysis

MBP-hTERT in solution digestion was performed using trypsin (1/100, w/w, Sequencing Grade, Promega) for at least 4 h at 37°C in 25 mM ammonium bicarbonate. Peptides were analyzed by nano-LC–MS/MS using an Ultimate 3000 system (Dionex S.A.) coupled to an LTQ-Orbitrap XL mass spectrometer (Thermo Fisher Scientific, Bremen, Germany). Samples were loaded on a C18 precolumn (300 μm inner diameter × 5 mm; Dionex) at 20 μl/min in 5% acetonitrile, 0.1% TFA. After 3 min of desalting, the precolumn was switched on line with the analytical C18 column (75 μm inner diameter × 50 cm; C18 PepMap^TM^, Dionex) equilibrated in solvent A (2% acetonitrile, 0.1% formic acid). Bound peptides were eluted using a 100 min linear gradient (from 0 to 30% (v/v)) of solvent B (80% acetonitrile, 0.085% formic acid) at a 150 nl/min flow rate and an oven temperature of 40°C. Data-dependent acquisition was performed on the LTQ-Orbitrap mass spectrometer in the positive ion mode. Survey MS scans were acquired in the Orbitrap on the 475–1200 *m/z* range with the resolution set to a value of 60 000. Each scan was recalibrated in real time by co-injecting an internal standard from ambient air into the C-trap (‘lock mass option’). The five most intense ions per survey scan were selected for CID fragmentation and the resulting fragments were analyzed in the linear trap (LTQ). Target ions already selected for MS/MS were dynamically excluded for 180 s.

### Mass spectrometry data analysis

Data were acquired using the Xcalibur software (version 2.2) and the resulting spectra were then analyzed *via* the Sequest HT Softwares created with Proteome Discoverer (version 1.4.0.288, Thermo Scientific) using the SwissProt *P. pastoris* database, which contains keratins and MBP-hTERT protein sequences (9933 queries). Carbamidomethylation of cysteines, oxidation of methionine, protein N-terminal acetylation were set as variable modifications. Specificity of trypsin digestion was set and four missed cleavage sites were allowed. The mass tolerances in MS and MS/MS were set to 2 ppm and 0.5 Da, respectively. The result files were loaded into the myProMS(29) server for further processing. In myProMS we fixed the estimated false discovery rate (FDR) of all peptide and protein identifications to <1%.

## RESULTS

### Optimization of hTERT production

**Figure 1.**
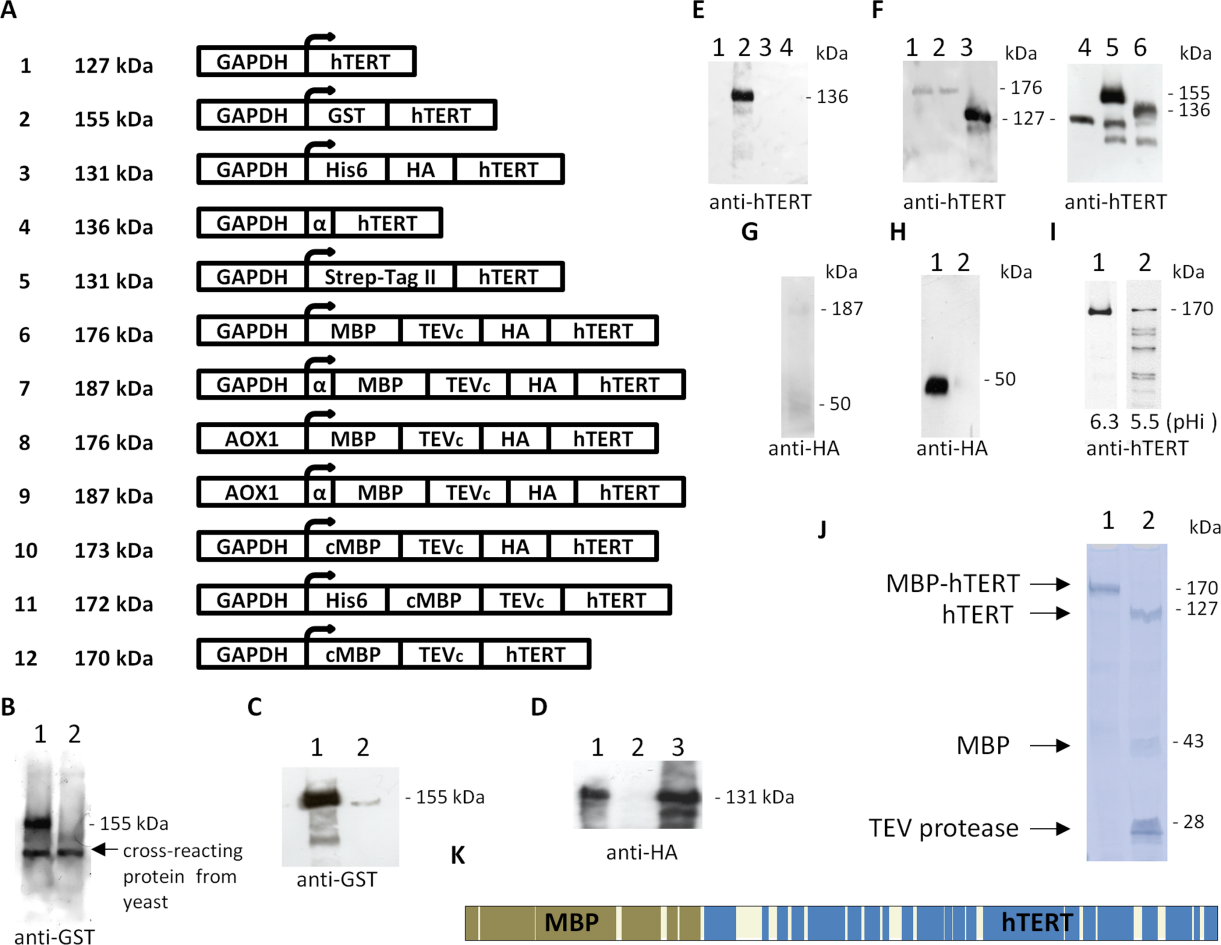
Production of hTERT in yeast. (**A**) Constructs used to produce hTERT. For each construct, the water-soluble fraction of the yeast lysate was subjected to SDS-PAGE, followed by western blot. (**B**) Detection of GST-hTERT (construct 2) in transformed (lane 1) or wild-type yeast lysate (lane 2). (**C**) GST-hTERT remains in the flow (lane 1), and only a tiny fraction binds to a glutathion-column (lane 2). (**D**) His6-HA-hTERT (construct 3) was immunoprecipitated with an anti-HA tag antibody (lane 1). A control was performed by omitting the antibody (lane 2) and the input was loaded (lane 3). (**E**) Intracellular (lanes 1 and 2) and extracellular (lanes 3 and 4) fractions of wild type yeast (lanes 1 and 3) or yeast expressing α-hTERT (lanes 2 and 4, construct 4). (**F**) Comparison of the expression level of two different clones transformed with MBP-hTERT (lanes 1 and 2, construct 6), with yeast expressing hTERT (lanes 3 and 4, construct 1), GST-hTERT (lane 5, construct 2) and α-hTERT (lane 6, construct 4). (**G**) α-MBP-hTERT (construct 7). (**H**) MBP-hTERT (lane 1, construct 8) and α-MBP-hTERT (lane 2, construct 9) after induction with 1% methanol. Only a degradation product is detectable. (**I**) cMBP-hTERT (construct 12) from yeast grown until the intracellular pH reaches 6.3 (lane 1) or 5.5 (lane 2). (**J**) Coomassie brilliant blue stained SDS-PAGE of soluble protein fractions of purified cMBP-hTERT (construct 10), after amylose resin purification (lane 1), and cleavage of the MBP tag by His6-TEV protease (lane 2). (**K**) Protein sequence coverage. Purified cMBP-hTERT (construct 12) was digested with trypsin, and the peptides were analyzed by nano-LC–MS/MS. The sequence coverage was near 84%. MBP is shown in brown, hTERT in blue. Data are representative of at least three independent experiments. Figure (J) is representative of over 100 experiments.

Like others, we failed to produce recombinant hTERT in bacteria (10–13), and could difficulty generate a few micrograms of partially purified protein using the previously described methods based on insect cells (13–16). While searching for a more powerful alternative, we found that hTERT could be constitutively expressed in the yeast *P. pastoris* using the glyceraldehyde 3-phosphate dehydrogenase promoter (GAPDH). Interestingly, the extraction of hTERT expressed in this system did not required denaturants, detergents or high salts after mechanical lysis, and the protein was readily soluble in the supernatant after centrifugation. Despite large cultures of yeast cells expressing hTERT could be generated, the protein was not easy to isolate. Its purification required a long optimization process during which several constructs have been successively tested (Figure 1A).

We tagged hTERT with the glutathione S-transferase (GST) (Figure 1B) or with a polyhistidine-tag (His6), but the binding of these fusion proteins to their affinity matrix was strikingly inefficient (Figure 1C). This could be due to an inaccessibility of the N-terminal region. However, contrary to a previous report (13), His6-HA-hTERT could be immunoprecipitated with an antibody against the hemagglutinin tag (HA) (Figure 1D). To facilitate the purification, we then tried to promote the secretion of hTERT with the peptide signal of the *Saccharomyces cerevisiae* alpha-factor (α), but the protein remained intracellular (Figure 1E). No protein could be detected after the fusion of hTERT to the Strep-Tag II, while a fusion to the *Escherichia coli* maltose-binding protein (MBP) was also strongly detrimental to the expression level, as seen by the comparison with untagged hTERT and previous constructs (Figure 1F). Interestingly, the MBP-hTERT protein seemed to bind to its affinity matrix, but because of its low expression level, no significant amount of purified protein could be recovered. To allow the efficient production of MBP-hTERT, several strategies were then tested. First, we added the peptide signal of the alpha-factor (α) to MBP-hTERT, but the protein became barely detectable, only intracellulary with the apparition of degradation products (Figure 1G). Secondly, to try to increase their expression level, the two MBP-fusions were tested with the alcohol oxidase (AOX1) promoter, but only a degradation product was detected with the construct devoided of the peptide signal of the α-factor (Figure 1H). Altogether, these observations show that contrary to the situation described in insect cells (14), the addition of a secretion signal did not improve hTERT expression in this system (Figure 1F), and was even detrimental in association with the MBP (Figure 1G and H). Noticeably, the MBP contains an endogenous periplasmic targeting sequence that is predicted by the signalP algorithm (30) to be recognized as a signal peptide by eukaryotic cells. We therefore generated alternative MBP-hTERT constructs deleted of this leader sequence (cMBP). These new vectors finally allowed the production of an unprecedented amount of purified hTERT when the yeasts were grown in special conditions. Indeed, we found that the expression of hTERT was decorrelated from yeast growth, because this protein was degraded early during the acidification induced by glucose metabolism (Figure 1I). Therefore, the correct expression of hTERT required the careful monitoring of the culture, and the selection of conditions where the intracellular pH measured after cell lysis has not dropped below 6. When using yeast transformed with constructs 10, 11 or 12 (Figure 1A) and grown in these optimized conditions, 1 mg of MBP-hTERT was recovered from a 3-l culture in a simple and cost-effective manner. After a one-step purification with an amylose–resin, MBP-hTERT was detected as the most abundant protein by Coomassie brilliant blue staining and by a proteomic LC–MS/MS approach (Figure 1J and K, Supplementary Figure S3C. and Tables S1 and S2). Purified MBP-hTERT (170 kDa) could be reconcentrated from 0.1 to 2 mg/ml by ultrafiltration using a membrane with a 100 kDa cut-off, while it completely passed through a membrane with a 300 kDa cut-off. Moreover, the MBP tag could be efficiently cleaved by the TEV protease, without inducing protein precipitation (Figure 1J). Altogether, these analyses demonstrate that hTERT produced in this system is not aggregated.

### *In vitro* telomerase reconstitution with purified hTERT

**Figure 2.**
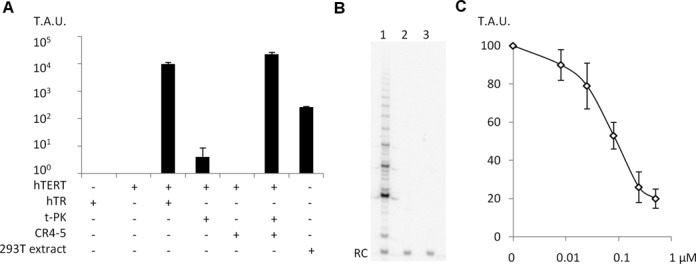
*In vitro* telomerase reconstitution. (**A**) Telomerase activity measured by RT-TRAP assays performed with MBP-hTERT, full length hTR, the t-PK truncation (hTR 33–209 nt), and the CR4–5 truncation (hTR 207–325 nt) assembled with 50 ng each. For comparison, telomerase activity was measured in an extract of HEK 293T cells containing 50 ng of protein (error bar ± SD, *n* = 3). Telomerase activity is expressed in arbitrary units (T.A.U.). (**B**) Detection of telomerase activity by direct assay in a sample containing both MBP-hTERT and hTR (300 nM each) (lane 1), or either hTR (lane 2) or MBP-hTERT alone (lane 3). RC: recovery control. (**C**) Recombinant telomerase (10 nM) was assayed by RT-TRAP with increasing concentrations of BIBR1532 (IC_50_ ≈ 0.08 μM). Error bars show standard deviation from at least three independent experiments.

In previous systems (18,19), purified hTERT failed to reconstitute detectable telomerase activity when combined with *in vitro* transcribed hTR. On the contrary, the use of hTERT (50 ng) produced with this new method readily displayed a telomerase activity 49-fold higher than the level detectable in an extract of HEK 293T cells containing the same total protein concentration (Figure 2A and Supplementary Figure S5A). The activity of the recombinant enzyme was detected using the real-time telomere repeat amplification protocol (RT-TRAP) and confirmed by direct assay that showed the processivity (Figure 2B). It was also inhibited by BIBR1532, a previously described inhibitor of human telomerase (31) (Figure 2C). The activity based on the hTERT molecule number of this recombinant telomerase assembled *in vitro* was ∼10^6^ less than the one found in HEK 293T cells (32). To our knowledge, the efficiency of telomerase reconstitution in rabbit reticulocyte lysate or insect cells has not been compared to human cells, but our observation is consistent with the view that several essential factors assist the proper assembly of telomerase *in vivo* (3–5).

### hTR folding limits telomerase reconstitution *in vitro*

**Figure 3.**
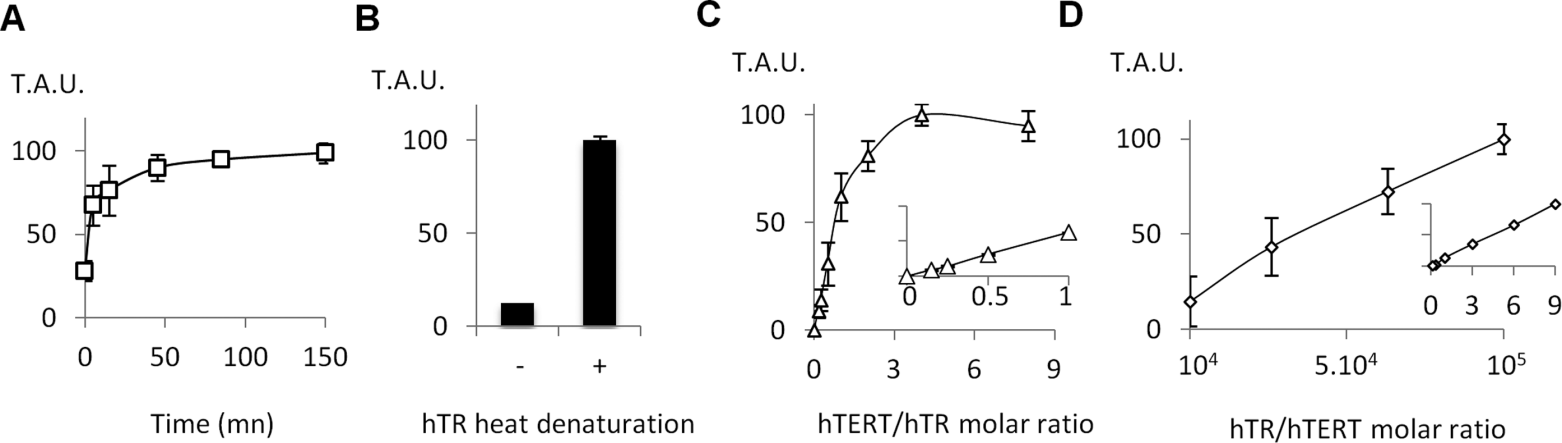
hTR limits telomerase reconstitution (**A**) Kinetic of *in vitro* telomerase reconstitution. hTR and MBP-hTERT were combined for different durations at room temperature, and telomerase activity was measured by RT-TRAP. (**B**) hTR folding. A 2-min heat-denaturation of hTR at 70°C increased the reconstituted activity level eightfold. (**C**) Telomerase activity measured by RT-TRAP assays performed with a fixed concentration of hTR (25 nM) and increasing concentrations of MBP-hTERT (main curve and insert). (**D**) Telomerase activity measured by RT-TRAP assays performed with 25 pM (main curve) or 25 nM (insert) MBP-hTERT and increasing concentrations of hTR. Error bars show standard deviation from at least three independent experiments.

We attempted to identify the factors limiting telomerase assembly *in vitro*. First, we found that telomerase reconstitution proceeded rapidly at room temperature and continued to increase slightly over-time, showing that the recombinant enzyme was stable (Figure 3A). The purified MBP-hTERT protein did not reduce the telomerase activity of a cancer cell extract; therefore the preparation did not contain a telomerase inhibitor. Furthermore, MBP-hTERT purified with low or high salt buffers reconstituted similar activity. The addition of ATP (10 mM), fresh yeast lysate, or the Hsp90 inhibitor geldanamycin (100 μM) during the reconstitution did not affect the level of telomerase activity generated; therefore we did not find evidence for the contribution of a protein cofactor co-purified from yeast. Besides, we noticed that the reconstitution of telomerase was highly dependent on the folding state of hTR (Figure 3B). Moreover, whereas titration experiments with increasing amounts of hTERT and constant amounts of hTR showed that telomerase activity increased lineary and then reached a plateau at concentrations slightly higher than the equimolar ratio (Figure 3C), we were not able to reach saturation for telomerase activity by adding varying amounts of hTR to a constant concentration of hTERT, even with a 10^5^ fold molar excess (Figure 3D). These results suggest that most of the *in vitro* transcribed hTR is not properly folded to form an active telomerase complex. This view is in agreement with a previous analysis which suggested that cellular protein cofactors are required to fold hTR (33). As hTERT may preferentially bind to the properly folded hTR fraction, more active telomerase complexes may be formed when an excess of RNA is provided. Also, hTR may complement some inactive complexes in *trans* (34,35). On the contrary, the addition of hTR to the telomerase extracted from HEK 293T cells did not affect its activity. A three-fold higher level of telomerase was obtained when the recombinant enzyme was reconstituted with two complementary hTR truncations containing the template-pseudoknot (t-PK) and the conserved regions 4 and 5 (CR4–5), which are believed to reduce the folding heterogeneity (36) (Figure 2A). Altogether, our observations suggest that hTR folding is the major factor limiting telomerase assembly *in vitro* and that much higher levels of telomerase activity will be available as soon as the factors required to fold hTR correctly have been identified.

### Screening for inhibitors of telomerase assembly

**Figure 4.**
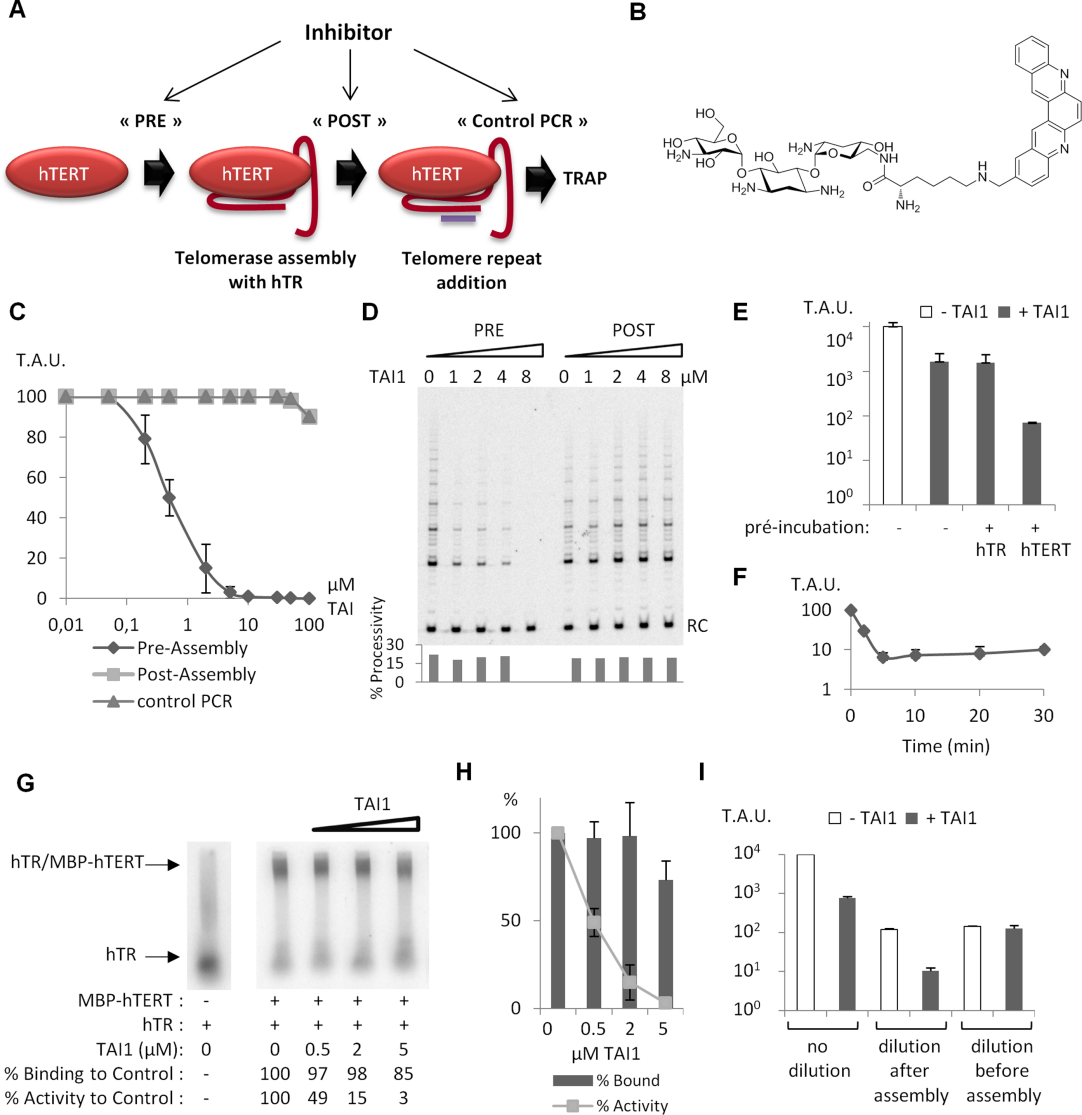
Identification and characterization of the Telomerase Assembly Inhibitor 1 (TAI1). (**A**) Scheme of the screening assay. In order to distinguish assembly from catalytic inhibitors, and to exclude false positives, each hit identified in the first screen was added before telomerase assembly (PRE), after assembly (POST) or after primer elongation (PCR control). (**B**) Chemical structure of TAI1. (**C**) Telomerase activity quantified by RT-TRAP. The assembly assay was performed with MBP-hTERT and hTR (100 nM each) and increasing concentrations of TAl1 added at different steps. Telomerase activity is expressed in Arbitrary Units (T.A.U.). (**D**) Telomerase activity detected by direct assay. Recombinant telomerase (300 nM) was incubated with increasing concentrations of TAI1 at the pre- or post- assembly step. RC: recovery control. Quantification of the repeat addition processivity for each lane (bottom). (**E**) Telomerase activity measured by RT-TRAP assays performed with hTERT and hTR (300 nM each) with an additional preincubation step of 10 min of TAl1 (20 μM) with either MBP-hTERT or hTR (error bar ± SD, *n* = 3). (**F**) Time-dependence effect of the preincubation step of TAI1 (5 μM) with hTERT. (**G**) EMSA with radioactive labelled hTR (170 nM) and MBP-hTERT (300 nM) in the presence of increasing concentrations of TAI1. Telomerase reconstitution was also performed with unlabeled hTR to quantify telomerase activity by RT-TRAP. (**H**) Quantification of the relative hTERT/hTR association measured by EMSA and enzymatic activity by RT-TRAP assays in the presence of increasing concentrations of TAI1 (error bar ± SD, *n* = 3). (**I**) The hTERT-hTR telomerase complex (300 nM) formed in the presence of TAI1 (5 μM) was diluted 100-fold (after assembly), and telomerase activity was measured by RT-TRAP. Alternatively, MBP-hTERT (300 nM) was preincubated for 10 min with TAI1 (5 μM), diluted 100-fold (before assembly) and incubated again 10 min, Then telomerase was reconstitued by the addition of hTR (300 nM) and the activity was measured as above (error bar ± SD, *n* = 3). In the non-diluted samples 92% of the activity was inhibited. In the samples diluted after assembly 91% of the activity was inhibited. In the samples diluted before assembly 11% of the activity was inhibited. Error bars show standard deviation from at least three independent experiments.

The possibility to reconstitute telomerase *in vitro* with minimal components offers a unique means to identify regulators of the assembly of the telomerase core enzyme. As we found that the purification of hTERT and the reconstitution of active telomerase were highly reproducible using this new methodology, enough telomerase components are therefore now available to perform a large number of telomerase reconstitution tests, in order to identify small-molecules capable of interfering with telomerase assembly. As a proof of concept, we decided to validate this new approach by screening a medium-sized chemical library consisting of 3000 compounds issued from the proprietary chemical library of the Institut Curie-CNRS. In a first screen, the molecules were added before telomerase assembly, and the reconstituted activity was measured by RT-TRAP in multiwell plates. This provided a direct measurement of telomerase activity and enabled rapid data analysis, without the need to perform time-consuming gel migrations and band quantifications (24). Compounds displaying more than 70% inhibition were then reassayed under three conditions: each compound was added either before telomerase assembly (pre-assembly condition), after assembly (post-assembly condition), or after primer elongation (Figure 4A). Molecules inhibiting telomerase activity only in the pre-assembly condition were scored as assembly inhibitors, whereas molecules inhibiting telomerase activity both in the pre- and post-assembly conditions were classified as catalytic inhibitors of telomerase. Finally, compounds inhibiting telomerase activity in all conditions were identified as poorly selective molecules or potential false positives interfering with the PCR step.

Using this protocol, we identified a new molecule, TAI1 (Figure 4B), which strongly inhibited telomerase activity when added prior to the assembly of the enzyme, while displaying no effect on an already assembled telomerase at the same concentration (Figure 4C and D). Similar properties were also found with another, albeit less potent compound (Supplementary Figure S5B). These observations suggest that the binding site of some telomerase inhibitors is no longer accessible after the telomerase complex is formed. Direct assay showed that TAI1 prevents the DNA polymerase activity of telomerase, while it had no detectable effect on the translocation of the enzyme (Figure 4D).

### Characterization of the mode of inhibition by TAI1

In order to determine whether hTERT or hTR is the target of the drug, we compared the telomerase inhibition levels obtained when TAI1 was preincubated with either hTERT or hTR before assembly. A preincubation step of MBP-hTERT with TAI1 strongly increased inhibition potency, while the same procedure with hTR provided no benefit compared to the parallel experiment performed without preincubation (Figure 4E). This suggests that hTERT is the main target of the drug. A 5 min preincubation with hTERT was required for maximal TAI1 efficiency, while longer preincubation did not increased the inhibition further (Figure 4F). As hTR and hTERT associate through at least three independent interactions (37), a small molecule is unlikely to compete with all sites concomitantly. Indeed, EMSA showed that TAI1 only slightly reduced the binding of hTERT to hTR, in conditions where telomerase activity was completely inhibited (Figure 4G and H). As, the recombinant telomerase assembled *in vitro* contains misassembled subunits because of the heterogeneous folding of hTR, we could not follow the effect of TAI1 on the assembly of the active telomerase only. However, if the compound abolished the interaction between hTERT and the properly folded hTR fraction, it would also have suppressed the association with the partially unfolded hTR, as the affinity of hTERT for the inactive RNA is expected to be lower. Therefore, these observations suggest that TAI1 allowed the formation of ribonucleoprotein complexes, which were inactive. Surprisingly, these inactive complexes were irreversibly inhibited, since they cannot be rescued by a 100-fold dilution, even after several hours of incubation (Figure 4I). On the contrary, no telomerase inhibition was observed if MBP-hTERT preincubated with TAI1 was diluted before assembly (Figure 4I). Altogether, these findings suggest that TAI1 reversibly interacts with hTERT before assembly, but is irreversibly trapped inside the hTERT-hTR complex after assembly, in a manner that is reminiscent of interfacial inhibitors (38).

### Structure–activity relationship studies

**Figure 5.**
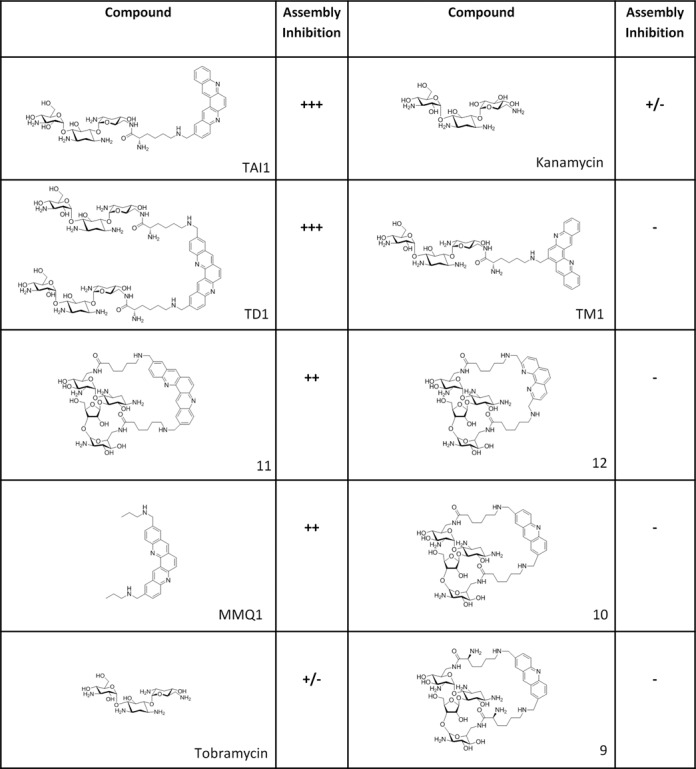
Structure–activity relationships of TAI1. Several TAI1-related compounds were tested by RT-TRAP for their inhibitory activity against telomerase assembly. +++ (IC_50_ = 0.5 ± 0.2 μM); ++ (IC_50_ = 6 ± 2 μM); +/− (IC_50_ = 400 ± 100 μM); − (no detectable effect on telomerase assembly). TD1, TM1 and the compound 11 have been previously described (26), as well as MMQ1 (27) and the Neomycin bis-lysine acridine macrocycles (compounds 12,10, 9) (28). Data are representative of three independent experiments.

**Figure 6.**
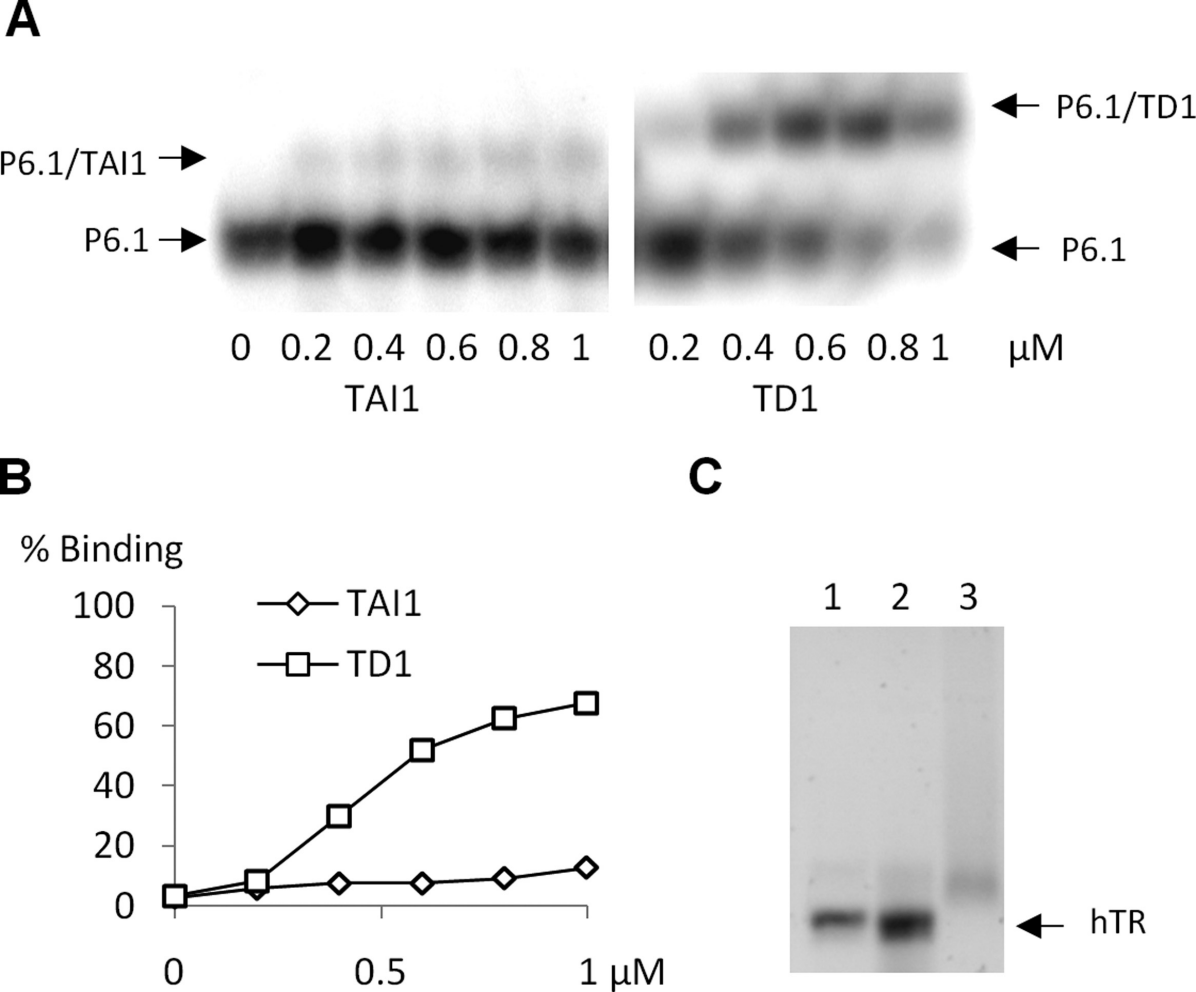
TAI1 and TD1 binding to hTR. (**A**) The binding ability of TAI1 or TD1 to the P6.1 helix of human telomerase RNA (1 μM) was followed by EMSA (26). (**B**) Quantification of the EMSA. (**C**) Agarose gel migration of full length hTR incubated alone (lane 1), or with a 50 molar excess of TAI1 (lane 2) or TD1 (lane 3). Data are representative of three independent experiments.

TAI1 belongs to the quinacridine-aminoglycoside conjugate family (26). The effect of TAI1 on telomerase assembly was therefore compared to the one of other compounds from this series (Figure 5). The most potent inhibitors were the quinacridine–tobramycin conjugates TAI1 and TD1, although these two molecules showed striking differences in an RNA binding assay using the P6.1 helix of hTR and full length hTR (Figure 6). Also, TD1 inhibited PCR in contrast to TAI1. The analogue TM1, in which the quinacridine residue is linked with the tobramycin at a different position, was inactive. A non-sugar modified quinacridine derivative (MMQ1) or a quinacridine analogue linked to a neomycin residue (compound 11) were moderately active, while the related acridine-neomycin conjugates (compounds 12, 10, 9) were inactive. This suggests a critical role of the quinacridine residue for activity, which is enhanced by a spatially appropriate linkage to the tobramycin moiety. The unmodified aminoglycoside tobramycin, as well as kanamycin, were slightly active at high concentrations, consistent with a previous report showing that some aminoglycosides can interfere with telomerase assembly (7). Altogether, these observations demonstrate that TAI1 is composed of two synergic components, and display a specific ability to inhibit telomerase assembly compared to other related molecules.

## DISCUSSION

Despite several attempts in different systems (11–20), the production of recombinant hTERT has remained limited by the impossibility to produce large amounts of soluble and functional protein. Moreover, only partial purification could be obtained using insect cells (13,14,16). The key reasons for the efficient production of hTERT with this system are the optimized culture conditions associated with the use of a constitutive expression in a rich medium, which promotes better protein quality than a rapid induction in stressful conditions. Additionally, the MBP tag turned out to be essential to obtain an efficient purification of hTERT. The MBP is often used in bacteria as a solubility-enhancer, but this effect does not seem to be the explanation here, because hTERT was found to be soluble without this tag. Moreover, His6-MBP-hTERT could not be purified efficiently using nickel-affinity. Interestingly, hTERT has been shown to functionally multimerize (19) and may therefore form large protein complexes when overexpressed. However, most commonly used tags usually fail to support the purification of large protein complexes because of their modest binding affinity. Notably, amylose-resins are made of long glucose polymers that impose less steric constraints for binding than most other matrix-bound ligands, and can therefore provide more accessible binding sites to large protein complexes. In consequence, the successful conditions described here may also apply to other proteins facing the same issues.

The possibility to reconstitute human telomerase with purified components is highly significant as even the super-telomerase system fail to generate a functional enzyme when hTERT and hTR are produced separately (39). Therefore, our methodology provides an unique tool for the study of telomerase structure and assembly. It may be used to identify the proteins required for the correct folding of hTR and also to clarify the function of the other factors that assist the proper assemblage of the telomerase complex. Furthermore, it may allow the large-scale screenings for telomerase inhibitors that cannot be discovered using conventional methods. Indeed, our study supports the view that a small-molecule can interfere efficiently with telomerase assembly. The analysis performed with the quinacridine-aminoglycoside TAI1 thereby suggests that such inhibitors might become stably trapped inside the telomerase complex. Notably, aminoglycosides are well-known RNA binders and among several quinacridine-aminoglycoside conjugates, TD1 has been reported to bind to the P6.1 helix of hTR *in vitro* (26), whereas TAI1 does not. Surprisingly, hTERT was found to be the main target of TAI1. The aminoglycoside part of this inhibitor was found to be important, but not essential, as the aromatic part only (the quinacridine residue) already displayed a significant inhibitory activity. Therefore, it might be speculated that the quinacridine part of TAI1 first binds to a hydrophobic pocket in the RNA binding interface of hTERT, and then the aminoglycoside part further disturbs hTR positioning, thus producing an inactive ternary complex.

These observations may furthermore implicate that potent telomerase inhibitors have probably escaped detection during all previous screens performed with an already assembled enzyme. The assay presented here should allow the identification of such molecules, which might lead to a real breakthrough in the treatment of cancers. Indeed, interfacial drugs have a remarkable potential for the specific inhibition of macromolecular complexes (40). Therefore, we anticipate that the present method may lead to the discovery of molecules with high clinical value.

## SUPPLEMENTARY DATA

Supplementary Data are available at NAR Online.

SUPPLEMENTARY DATA

## References

[1] Cohen S.B., Graham M.E., Lovrecz G.O., Bache N., Robinson P.J., Reddel R.R. (2007). Protein composition of catalytically active human telomerase from immortal cells. Science.

[2] Gardano L., Holland L., Oulton R., Le Bihan T., Harrington L. (2012). Native gel electrophoresis of human telomerase distinguishes active complexes with or without dyskerin. Nucleic Acids Res..

[3] Egan E.D., Collins K. (2012). Biogenesis of telomerase ribonucleoproteins. RNA.

[4] Tomlinson R.L., Ziegler T.D., Supakorndej T., Terns R.M., Terns M.P. (2006). Cell cycle-regulated trafficking of human telomerase to telomeres. Mol. Biol. Cell.

[5] Venteicher A.S., Meng Z., Mason P.J., Veenstra T.D., Artandi S.E. (2008). Identification of ATPases pontin and reptin as telomerase components essential for holoenzyme assembly. Cell.

[6] Harley C.B. (2008). Telomerase and cancer therapeutics. Nat. Rev. Cancer.

[7] Dominick P.K., Keppler B.R., Legassie J.D., Moon I.K., Jarstfer M.B. (2004). Nucleic acid-binding ligands identify new mechanisms to inhibit telomerase. Bioorg. Med. Chem. Lett..

[8] Holt S.E., Aisner D.L., Baur J., Tesmer V.M., Dy M., Ouellette M., Trager J.B., Morin G.B., Toft D.O., Shay J.W. (1999). Functional requirement of p23 and Hsp90 in telomerase complexes. Genes Dev..

[9] Keppler B.R., Jarstfer M.B. (2004). Inhibition of telomerase activity by preventing proper assemblage. Biochemistry.

[10] Jacobs S.A., Podell E.R., Wuttke D.S., Cech T.R. (2005). Soluble domains of telomerase reverse transcriptase identified by high-throughput screening. Protein Sci..

[11] Frohnert C., Hutten S., Walde S., Nath A., Kehlenbach R.H. (2014). Importin 7 and Nup358 promote nuclear import of the protein component of human telomerase. PLoS One.

[12] Akincilar S.C., Low K.C., Liu C.Y., Yan T.D., Oji A., Ikawa M., Li S., Tergaonkar V. (2015). Quantitative assessment of telomerase components in cancer cell lines. FEBS Lett..

[13] Masutomi K., Kaneko S., Hayashi N., Yamashita T., Shirota Y., Kobayashi K., Murakami S. (2000). Telomerase activity reconstituted in vitro with purified human telomerase reverse transcriptase and human telomerase RNA component. J. Biol. Chem..

[14] Wu C.K., Gousset K., Hughes S.H. (2007). Targeting to the endoplasmic reticulum improves the folding of recombinant human telomerase reverse transcriptase. Protein Expr. Purif..

[15] Mikuni O., Trager J.B., Ackerly H., Weinrich S.L., Asai A., Yamashita Y., Mizukami T., Anazawa H. (2002). Reconstitution of telomerase activity utilizing human catalytic subunit expressed in insect cells. Biochem. Biophys. Res. Commun..

[16] Wenz C., Enenkel B., Amacker M., Kelleher C., Damm K., Lingner J. (2001). Human telomerase contains two cooperating telomerase RNA molecules. EMBO J..

[17] Bah A., Bachand F., Clair E., Autexier C., Wellinger R.J. (2004). Humanized telomeres and an attempt to express a functional human telomerase in yeast. Nucleic Acids Res..

[18] Mizuno H., Khurts S., Seki T., Hirota Y., Kaneko S., Murakami S. (2007). Human telomerase exists in two distinct active complexes in vivo. J. Biochem..

[19] Moriarty T.J., Huard S., Dupuis S., Autexier C. (2002). Functional multimerization of human telomerase requires an RNA interaction domain in the N terminus of the catalytic subunit. Mol. Cell. Biol..

[20] Bachand F., Autexier C. (1999). Functional reconstitution of human telomerase expressed in Saccharomyces cerevisiae. J. Biol. Chem..

[21] Zaug A.J., Crary S.M., Jesse Fioravanti M., Campbell K., Cech T.R. (2013). Many disease-associated variants of hTERT retain high telomerase enzymatic activity. Nucleic Acids Res..

[22] Schnapp G., Rodi H.P., Rettig W.J., Schnapp A., Damm K. (1998). One-step affinity purification protocol for human telomerase. Nucleic Acids Res..

[23] D’Ambrosio D., Reichenbach P., Micheli E., Alvino A., Franceschin M., Savino M., Lingner J. (2012). Specific binding of telomeric G-quadruplexes by hydrosoluble perylene derivatives inhibits repeat addition processivity of human telomerase. Biochimie.

[24] Wege H., Chui M.S., Le H.T., Tran J.M., Zern M.A. (2003). SYBR Green real-time telomeric repeat amplification protocol for the rapid quantification of telomerase activity. Nucleic Acids Res..

[25] Bertrand H., Bombard S., Monchaud D., Teulade-Fichou M.P. (2007). A platinum-quinacridine hybrid as a G-quadruplex ligand. J. Biol. Inorg. Chem..

[26] Kaiser M., Sainlos M., Lehn J.M., Bombard S., Teulade-Fichou M.P. (2006). Aminoglycoside-quinacridine conjugates: towards recognition of the P6.1 element of telomerase RNA. Chembiochem.

[27] Hounsou C., Guittat L., Monchaud D., Jourdan M., Saettel N., Mergny J.L., Teulade-Fichou M.P. (2007). G-quadruplex recognition by quinacridines: a SAR, NMR, and biological study. ChemMedChem.

[28] Kaiser M., De Cian A., Sainlos M., Renner C., Mergny J.L., Teulade-Fichou M.P. (2006). Neomycin-capped aromatic platforms: quadruplex DNA recognition and telomerase inhibition. Org. Biomol. Chem..

[29] Poullet P., Carpentier S., Barillot E. (2007). myProMS, a web server for management and validation of mass spectrometry-based proteomic data. Proteomics.

[30] Petersen T.N., Brunak S., von Heijne G., Nielsen H. (2011). SignalP 4.0: discriminating signal peptides from transmembrane regions. Nat. Methods.

[31] Pascolo E., Wenz C., Lingner J., Hauel N., Priepke H., Kauffmann I., Garin-Chesa P., Rettig W.J., Damm K., Schnapp A. (2002). Mechanism of human telomerase inhibition by BIBR1532, a synthetic, non-nucleosidic drug candidate. J. Biol. Chem..

[32] Xi L., Cech T.R. (2014). Inventory of telomerase components in human cells reveals multiple subpopulations of hTR and hTERT. Nucleic Acids Res..

[33] Antal M., Boros E., Solymosy F., Kiss T. (2002). Analysis of the structure of human telomerase RNA in vivo. Nucleic Acids Res..

[34] Tesmer V.M., Ford L.P., Holt S.E., Frank B.C., Yi X., Aisner D.L., Ouellette M., Shay J.W., Wright W.E. (1999). Two inactive fragments of the integral RNA cooperate to assemble active telomerase with the human protein catalytic subunit (hTERT) in vitro. Mol. Cell. Biol..

[35] Rivera M.A., Blackburn E.H. (2004). Processive utilization of the human telomerase template: lack of a requirement for template switching. J. Biol. Chem..

[36] Kim N.K., Theimer C.A., Mitchell J.R., Collins K., Feigon J. (2010). Effect of pseudouridylation on the structure and activity of the catalytically essential P6.1 hairpin in human telomerase RNA. Nucleic Acids Res..

[37] Robart A.R., Collins K. (2011). Human telomerase domain interactions capture DNA for TEN domain-dependent processive elongation. Mol. Cell.

[38] Pommier Y., Marchand C. (2012). Interfacial inhibitors: targeting macromolecular complexes. Nat. Rev. Drug Discov..

[39] Cristofari G., Adolf E., Reichenbach P., Sikora K., Terns R.M., Terns M.P., Lingner J. (2007). Human telomerase RNA accumulation in Cajal bodies facilitates telomerase recruitment to telomeres and telomere elongation. Mol. Cell.

[40] Pommier Y., Cherfils J. (2005). Interfacial inhibition of macromolecular interactions: nature's paradigm for drug discovery. Trends Pharmacol. Sci..

