# Supplementary material for: Identification of human telomerase assembly inhibitors enabled by a novel method to produce hTERT
Source: Nucleic Acids Res. 2015 May 9;43(15):e99. doi: 10.1093/nar/gkv425 (PMC4551907; doi:10.1093/nar/gkv425)
Supplement: SUPPLEMENTARY DATA [file supp_43_15_e99__index.html]

Identification of human telomerase assembly inhibitors enabled by a novel method to produce hTERT — SUPPLEMENTARY DATA 

# Identification of human telomerase assembly inhibitors enabled by a novel method to produce hTERT

## SUPPLEMENTARY DATA

- SUPPLEMENTARY DATA
