# Supplementary material for: Identification of human telomerase assembly inhibitors enabled by a novel method to produce hTERT
Source: Nucleic Acids Res. 2015 May 9;43(15):e99. doi: 10.1093/nar/gkv425 (PMC4551907; doi:10.1093/nar/gkv425)
Supplement: SUPPLEMENTARY DATA [file supp_gkv425_nar-00372-f-2015-File008.pdf]

Identification of human telomerase assembly inhibitors enabled by a novel method to produce hTERT

Guillaume Kellermann[1,2], Markus Kaiser[3,4], Florent Dingli[5], Olivier Lahuna[6], Delphine Naud-Martin[3], Florence Mahuteau-Betzer[3], Damarys Loew[5], Evelyne Ségal-Bendirdjian[1,2], Marie-Paule Teulade-Fichou[3] and Sophie Bombard[1,2]

[1] INSERM UMR-S 1007, Cellular Homeostasis and Cancer, Paris, France.
[2] Université Paris Descartes; Sorbonne Paris Cité, Paris, France.
[3] Institut Curie / CNRS UMR 176, Orsay, France.
[4] Present address: ZMB, Faculty of Biology, University of Duisburg-Essen, Essen, Germany.
[5] Institut Curie / laboratoire de spectrométrie de masse protéomique, Paris, France.
[6] INSERM U-1016, Institut Cochin, Paris, France.

Supplementary Data

- Figures S1-S5

-Table S1-S2

AGATCTTTTTTGTAGAAATGTCTTGGTGTCCTCGTCCAATCAGGTAGCCATCTCTGAAATATCTGGCTCCGTTGCAACTCCGAACGACCTGCTGGCAACGTAAAATTCT
CCGGGGTAAAACTTAAATGTGGAGTAATGGAACCAGAAACGTCTCTTCCCTTCTCTCTCCTTCCACCGCCCGTTACCGTCCCTAGGAAATTTTACTCTGCTGGAGAGCT
TCTTCTACGGCCCCCTTGCAGCAATGCTCTTCCCAGCATTACGTTGCGGGTAAAACGGAGGTCGTGTACCCGACCTAGCAGCCCAGGGATGGAAAAGTCCCGGCCGT
CGCTGGCAATAATAGCGGGCGGACGCATGTCATGAGATTATTGGAAACCACCAGAATCGAATATAAAAGGCGAACACCTTTCCCAATTTTGGTTTCTCCTGACCCAAA
GACTTTAAATTTAATTTATTTGTCCCTATTTCAATCAATTGAACAACTATCAAAACACA**ATG**AAGATCGAAGAGGGAAAGTTGGTTATCTGGATCAACGGTGACAAGG
GTTACAACGGTTTGGCTGAGGTTGGAAAGAAGTTCGAGAAGGACACTGGTATCAAGGTTACTGTTGAGCACCCAGACAAGTTGGAAGAGAAGTTCCCACAAGTTGC
TGCTACTGGAGATGGTCCAGACATCATTTTCTGGGCTCACGACAGATTCGGTGGTTACGCTCAATCTGGTTTGTTGGCTGAGATCACTCCAGACAAGGCTTTCCAGGA
TAAGTTGTACCCATTCACTTGGGACGCTGTTAGATACAACGGAAAGTTGATCGCTTACCCAATCGCTGTTGAGGCTTTGTCCTTGATCTACAACAAGGACTTGTTGCCA
AACCCACCAAAGACTTGGGAAGAGATTCCAGCTTTGGACAAAGAGTTGAAGGCTAAGGGAAAGTCCGCTTTGATGTTCAACTTGCAAGAGCCATACTTCACTTGGCC
ATTGATTGCTGCTGATGGTGGATACGCTTTCAAGTACGAGAACGGTAAATACGACATCAAGGACGTTGGTGTTGACAACGCTGGTGCTAAGGCTGGTTTGACTTTCTT
GGTTGACTTGATTAAGAACAAGCACATGAACGCTGACACTGACTACTCCATTGCTGAGGCTGCTTTCAACAAGGGTGAGACTGCTATGACTATCAACGGTCCATGGG
CTTGGTCCAACATTGACACTTCCAAGGTTAACTACGGTGTTACTGTTTTGCCAACTTTCAAGGGTCAGCCATCCAAGCCATTCGTTGGAGTTTTGTCCGCTGGTATTAAC
GCTGCTTCCCCAAACAAAGAATTGGCTAAAGAGTTCTTGGAGAACTACTTGTTGACTGACGAGGGTTTGGAGGCTGTTAACAAGGATAAGCCATTGGGTGCTGTTGC
TTTGAAGTCCTACGAAGAAGAGTTGGCGAAGGACCCAAGAATCGCTGCTACTATGGAAAACGCTCAAAAGGGTGAAATCATGCCAAACATCCCACAGATGTCCGCTT
TCTGGTATGCTGTTAGAACTGCTGTTATCAATGCTGCTTCCGGTAGACAGACTGTTGACGAGGCTTTGAAGGACGCTCAAACTAACTCCTCCTCCAACAACAACAACA
ACAATAACAACAACAACGACTACGACCCAACTACT**GAGAACTTGTACTTCCAG**GGT**ATG**CCAAGAGCACCAAGATGTAGAGCTGTTAGATCTTTGTTGAGATCCCACT
ACAGAGAGGTTTTGCCATTGGCTACTTTCGTTAGAAGATTGGGTCCACAGGGTTGGAGATTGGTTCAAAGAGGAGATCCAGCTGCTTTTAGAGCTTTGGTTGCTCAG
TGTTTGGTTTGTGTTCCATGGGATGCTAGACCACCACCAGCTGCTCCATCTTTTAGACAGGTTTCTTGTTTGAAAGAGTTGGTTGCTAGAGTTTTGCAGAGATTGTGTG
AGAGAGGTGCTAAGAACGTTTTGGCTTTCGGTTTCGCTTTGTTGGACGGTGCTAGAGGTGGTCCACCAGAGGCTTTTACTACTTCAGTTAGATCCTACTTGCCAAACA
CTGTTACTGACGCTTTGAGAGGTTCTGGTGCTTGGGGTTTGTTGTTGAGAAGAGTTGGAGATGACGTTTTGGTTCACTTGTTGGCTAGATGTGCTTTGTTCGTTTTGG
TTGCTCCATCCTGTGCTTACCAGGTTTGTGGTCCTCCATTGTACCAATTGGGAGCTGCTACTCAAGCTAGACCTCCTCCTCATGCTTCTGGACCTAGAAGAAGATTGGG
ATGTGAGAGAGCTTGGAACCACTCCGTTAGAGAAGCTGGTGTTCCATTGGGTTTGCCAGCTCCAGGTGCTAGAAGAAGAGGTGGTTCTGCTTCCAGATCTTTGCCTTT
GCCAAAGAGACCAAGAAGAGGTGCTGCTCCAGAACCAGAAAGAACTCCAGTTGGTCAAGGTTCTTGGGCTCATCCAGGTAGAACTAGAGGTCCATCTGACAGAGGT
TTCTGTGTTGTTTCTCCAGCTAGACCAGCTGAAGAGGCTACTTCTTTGGAAGGTGCTTTGTCCGGTACTAGACACTCTCATCCATCCGTTGGTAGACAACATCATGCTG
GTCCACCTTCTACTTCCAGACCACCAAGACCATGGGATACTCCATGTCCACCAGTTTACGCTGAGACTAAGCACTTCTTGTACTCCTCCGGTGACAAAGAGCAATTGAG
ACCTTCCTTCTTGTTGTCCTCCTTGAGACCATCTTTGACTGGTGCTAGAAGATTGGTTGAGACTATCTTCTTGGGTTCCAGACCTTGGATGCCAGGTACTCCTAGAAGAT
TGCCTAGATTGCCACAGAGATACTGGCAGATGAGACCTTTGTTCTTGGAGTTGTTGGGTAACCACGCTCAGTGTCCATACGGTGTTTTGTTGAAAACTCACTGTCCATT
GAGAGCTGCTGTTACTCCTGCTGCTGGTGTTTGTGCTAGAGAAAAGCCACAAGGATCTGTTGCTGCTCCTGAGGAAGAAGATACTGACCCTAGAAGATTGGTTCAGT
TGTTGAGACAGCACTCCTCACCATGGCAAGTTTACGGATTCGTTAGAGCTTGTTTGAGAAGATTGGTTCCACCAGGTTTGTGGGGTTCCAGACACAACGAAAGAAGA
TTCTTGAGAAACACAAAGAAGTTCATCTCATTGGGAAAGCACGCTAAGTTGTCCTTGCAAGAATTGACTTGGAAGATGTCTGTTAGAGACTGTGCTTGGTTGAGAAG
ATCCCCAGGTGTTGGTTGTGTTCCAGCTGCTGAACATAGATTGAGAGAAGAGATCTTGGCTAAGTTCTTGCACTGGTTGATGTCCGTTTACGTTGTTGAGTTGTTGAG
ATCTTTCTTCTACGTTACTGAAACTACTTTCCAGAAGAACAGATTGTTCTTCTACAGAAAGTCTGTTTGGTCCAAGTTGCAGTCCATCGGTATCAGACAACACTTGAAGA
GAGTTCAATTGAGAGAGTTGTCCGAAGCTGAGGTTAGACAGCACAGAGAGGCTAGACCTGCTTTGTTGACTTCAAGATTGAGATTCATCCCAAAGCCAGACGGTTTG
AGACCAATCGTTAACATGGACTATGTTGTTGGTGCTAGAACTTTCAGAAGAGAGAAGAGAGCTGAGAGATTGACTTCCAGAGTTAAGGCTTTGTTCTCCGTTTTGAAC
TACGAGAGAGCTAGAAGACCAGGTTTGTTGGGAGCTTCCGTTTTGGGTTTGGACGACATTCATAGAGCTTGGAGAACATTCGTTTTGAGAGTTAGAGCACAGGACCC
ACCACCTGAGTTGTACTTCGTTAAGGTTGACGTTACTGGTGCTTACGACACTATCCCACAGGACAGATTGACTGAGGTTATCGCTTCCATCATCAAGCCACAGAACACT
TACTGTGTTAGAAGATACGCTGTTGTTCAAAAGGCTGCTCACGGTCACGTTAGAAAGGCTTTTAAGTCCCACGTTTCCACTTTGACTGACTTGCAGCCTTACATGAGAC
AGTTCGTTGCTCACTTGCAAGAAACTTCCCCATTGAGAGATGCTGTTGTTATCGAGCAGTCATCTTCCTTGAACGAGGCTTCTTCCGGTTTGTTCGACGTTTTCTTGAGA
TTCATGTGTCACCACGCTGTTAGAATCAGAGGAAAGTCCTACGTTCAATGTCAGGGTATCCCACAAGGTTCCATCTTGTCCACTTTGTTGTGTTCCTTGTGTTACGGTG
ACATGGAAAACAAGTTGTTCGCTGGAATTAGAAGAGATGGTTTGTTGTTGAGATTGGTTGACGACTTCTTGTTGGTTACTCCACACTTGACTCACGCTAAGACTTTCTT
GAGAACTTTGGTTAGAGGTGTTCCAGAGTACGGTTGTGTTGTTAACTTGAGAAAGACTGTTGTTAATTTCCCAGTTGAGGATGAGGCTTTGGGTGGTACTGCTTTCGT
TCAAATGCCAGCTCACGGTTTGTTCCCATGGTGTGGTTTGTTGTTGGACACTAGAACTTTGGAGGTTCAGTCCGACTACTCCTCCTACGCTAGAACTTCTATCAGAGCT
TCCTTGACTTTCAACAGAGGATTCAAGGCTGGTAGAAACATGAGAAGAAAGTTGTTCGGTGTTTTGAGATTGAAGTGTCACTCCTTGTTCTTGGACTTGCAGGTTAAC
TCCTTGCAGACTGTTTGTACTAATATCTACAAAATCTTGTTGTTGCAGGCTTACAGATTCCACGCTTGTGTTTTGCAGTTGCCATTCCACCAACAGGTTTGGAAGAACCC
AACATTCTTCTTGAGAGTTATCTCCGACACTGCTTCCTTGTGTTATTCCATCTTGAAGGCTAAGAACGCTGGAATGTCCTTGGGAGCTAAGGGTGCTGCTGGACCATTG
CCATCTGAAGCTGTTCAATGGTTGTGTCATCAGGCTTTCTTGTTGAAGTTGACTAGACACAGAGTTACTTACGTTCCTTTGTTGGGATCCTTGAGAACTGCTCAGACTC
AGTTGTCCAGAAAGTTGCCTGGTACTACTTTGACTGCTTTGGAAGCTGCTGCTAATCCTGCTTTGCCATCCGATTTCAAGACAATCTTGGAC***TAA***TAGGCGGCCGCATT
TCTAGAGTTTTAGCCTTAGACATGACTGTTCCTCAGTTCAAGTTGGGCACTTACGAGAAGACCGGTCTTGCTAGATTCTAATCAAGAGGATGTCAGAATGCCATTTGCC
TGAGAGATGCAGGCTTCATTTTTGATACTTTTTTATTTGTAACCTATATAGTATAGGATTTTTTTTGTCATTTTGTTTCTTCTCGTACGAGCTTGCTCCTGATCAGCCTATC
TCGCAGCTGATGAATATCTTGTGGTAGGGGTTTGGGAAAATCATTCGAGTTTGATGTTTTTCTTGGTATTTCCCACTCCTCTTCAGAGTACAGAAGATTAAGTGAGACC
TTCGTTTGTGCGGATCCCCCACACACCATAGCTTCAAAATGTTTCTACTCCTTTTTTACTCTTCCAGATTTTCTCGGACTCCGCGCATCGCCGTACCACTTCAAAACACCC
AAGCACAGCATACTAAATTTCCCTCTTTCTTCCTCTAGGGTGTCGTTAATTACCCGTACTAAAGGTTTGGAAAAGAAAAAAGAGACCGCCTCGTTTCTTTTTCTTCGTC
GAAAAAGGCAATAAAAATTTTTATCACGTTTCTTTTTCTTGAAATTTTTTTTTTTTAGTTTTTTTCTCTTTCAGTGACCTCCATTGATATTTAAGTTAATAAACGGTCTTCAA
TTTCTCAAGTTTCAGTTTCATTTTTCTTGTTCTATTACAACTTTTTTTACTTCTTGTTCATTAGAAAGAAAGCATAGCAATCTAATCTAAGGGCGGTGTTGACAATTAATC
ATCGGCATAGTATATCGGCATAGTATAATACGACAAGGTGAGGAACTAAACCATGGCCAAGTTGACCAGTGCCGTTCCGGTGCTCACCGCGCGCGACGTCGCCGGA
GCGGTCGAGTTCTGGACCGACCGGCTCGGGTTCTCCCGGGACTTCGTGGAGGACGACTTCGCCGGTGTGGTCCGGGACGACGTGACCCTGTTCATCAGCGCGGTCC
AGGACCAGGTGGTGCCGGACAACACCCTGGCCTGGGTGTGGGTGCGCGGCCTGGACGAGCTGTACGCCGAGTGGTCGGAGGTCGTGTCCACGAACTTCCGGGACG
CCTCCGGGCCGGCCATGACCGAGATCGGCGAGCAGCCGTGGGGGCGGGAGTTCGCCCTGCGCGACCCGGCCGGCAACTGCGTGCACTTCGTGGCCGAGGAGCAG
GACTGACACGTCCGACGGCGGCCCACGGGTCCCAGGCCTCGGAGATCCGTCCCCCTTTTCCTTTGTCGATATCATGTAATTAGTTATGTCACGCTTACATTCACGCCCT
CCCCCCACATCCGCTCTAACCGAAAAGGAAGGAGTTAGACAACCTGAAGTCTAGGTCCCTATTTATTTTTTTATAGTTATGTTAGTATTAAGAACGTTATTTATATTTCA
AATTTTTCTTTTTTTTCTGTACAGACGCGTGTACGCATGTAACATTATACTGAAAACCTTGCTTGAGAAGGTTTTGGGACGCTCGAAGGCTTTAATTTGCAAGCTGGAG
ACCAACATGTGAGCAAAAGGCCAGCAAAAGGCCAGGAACCGTAAAAAGGCCGCGTTGCTGGCGTTTTTCCATAGGCTCCGCCCCCCTGACGAGCATCACAAAAATCG
ACGCTCAAGTCAGAGGTGGCGAAACCCGACAGGACTATAAAGATACCAGGCGTTTCCCCCTGGAAGCTCCCTCGTGCGCTCTCCTGTTCCGACCCTGCCGCTTACCGG
ATACCTGTCCGCCTTTCTCCCTTCGGGAAGCGTGGCGCTTTCTCAATGCTCACGCTGTAGGTATCTCAGTTCGGTGTAGGTCGTTCGCTCCAAGCTGGGCTGTGTGCAC
GAACCCCCCGTTCAGCCCGACCGCTGCGCCTTATCCGGTAACTATCGTCTTGAGTCCAACCCGGTAAGACACGACTTATCGCCACTGGCAGCAGCCACTGGTAACAGG
ATTAGCAGAGCGAGGTATGTAGGCGGTGCTACAGAGTTCTTGAAGTGGTGGCCTAACTACGGCTACACTAGAAGGACAGTATTTGGTATCTGCGCTCTGCTGAAGCC
AGTTACCTTCGGAAAAAGAGTTGGTAGCTCTTGATCCGGCAAACAAACCACCGCTGGTAGCGGTGGTTTTTTTGTTTGCAAGCAGCAGATTACGCGCAGAAAAAAAG
GATCTCAAGAAGATCCTTTGATCTTTTCTACGGGGTCTGACGCTCAGTGGAACGAAAACTCACGTTAAGGGATTTTGGTCATGCATGAGATC

Fig. S1. Nucleotidic sequence of pGAPZ-MBP-hTERT plasmid vector (Fig. 1A, construct 12). In blue, the MBP coding sequence without its original periplasmic targeting sequence; in red, the linker; in bold black, the Tobacco Etch Virus (TEV) protease cleavage site (TEVc) (ENLYFQ); in green, hTERT.

MKIEEGKLVIWINGDKGYNGLAEVGKKFEKDTGIKVTVEHPDKLEEKFPQVAATGDGPDIIF
WAHDRFGGYAQSGLLAEITPDKAFQDKLYPFTWDAVRYNGKLIAYPIAVEALSLIYNKDLLP
NPPKTWEEIPALDKELKAKGKSALMFNLQEPYFTWPLIAADGGYAFKYENGKYDIKDVGV
DNAGAKAGLTFLVDLIKNKHMNADTDYSIAEAAFNKGETAMTINGPWAWSNIDTSKVNYG
VTVLPTFKGQPSKPFVGVLSAGINAASPNKELAKEFLENYLLTDEGLEAVNKDKPLGAVAL
KSYEEELAKDPRIAATMENAQKGEIMPNIPQMSAFWYAVRTAVINAASGRQTVDEALKDA
QTNSSSNNNNNNNNNNDYDPTT**ENLYFQ**GMPRAPRCRAVRSLLRSHYREVLPLATFVRR
LGPQGWRLVQRGDPAAFRALVAQCLVCVPWDARPPPAAPSFRQVSCLKELVARVLQRL
CERGAKNVLAFGFALLDGARGGPPEAFTTSVRSYLPNTVTDALRGSGAWGLLLRRVGDD
VLVHLLARCALFVLVAPSCAYQVCGPPLYQLGAATQARPPPHASGPRRRLGCERAWNHS
VREAGVPLGLPAPGARRRGGSASRSLPLPKRPRRGAAPEPERTPVGQGSWAHPGRTRG
PSDRGFCVVSPARPAEEATSLEGALSGTRHSHPSVGRQHHAGPPSTSRPPRPWDTPCP
PVYAETKHFLYSSGDKEQLRPSFLLSSLRPSLTGARRLVETIFLGSRPWMPGTPRRLPRLP
QRYWQMRPLFLELLGNHAQCPYGVLLKTHCPLRAAVTPAAGVCAREKPQGSVAAPEEED
TDPRRLVQLLRQHSSPWQVYGFVRACLRRLVPPGLWGSRHNERRFLRNTKKFISLGKHA
KLSLQELTWKMSVRDCAWLRRSPGVGCVPAAEHRLREEILAKFLHWLMSVYVVELLRSFF
YVTETTFQKNRLFFYRKSVWSKLQSIGIRQHLKRVQLRELSEAEVRQHREARPALLTSRLR
FIPKPDGLRPIVNMDYVVGARTFRREKRAERLTSRVKALFSVLNYERARRPGLLGASVLGL
DDIHRAWRTFVLRVRAQDPPPELYFVKVDVTGAYDTIPQDRLTEVIASIIKPQNTYCVRRYA
VVQKAAHGHVRKAFKSHVSTLTDLQPYMRQFVAHLQETSPLRDAVVIEQSSSLNEASSGL
FDVFLRFMCHHAVRIRGKSYVQCQGIPQGSILSTLLCSLCYGDMENKLFAGIRRDGLLLRL
VDDFLLVTPHLTHAKTFLRTLVRGVPEYGCVVNLRKTVVNFPVEDEALGGTAFVQMPAHG
LFPWCGLLLDTRTLEVQSDYSSYARTSIRASLTFNRGFKAGRNMRRKLFGVLRLKCHSLFL
DLQVNSLQTVCTNIYKILLLQAYRFHACVLQLPFHQQVWKNPTFFLRVISDTASLCYSILKA
KNAGMSLGAKGAAGPLPSEAVQWLCHQAFLLKLTRHRVTYVPLLGSLRTAQTQLSRKLP
GTTLTALEAAANPALPSDFKTILD

Fig. S2. Protein sequence of MBP-hTERT (Fig 1A, construct 12). In blue, the MBP protein sequence without its original periplasmic targeting sequence; in red, the linker; in bold black, the TEV cleavage site (ENLYFQ); in green, hTERT.

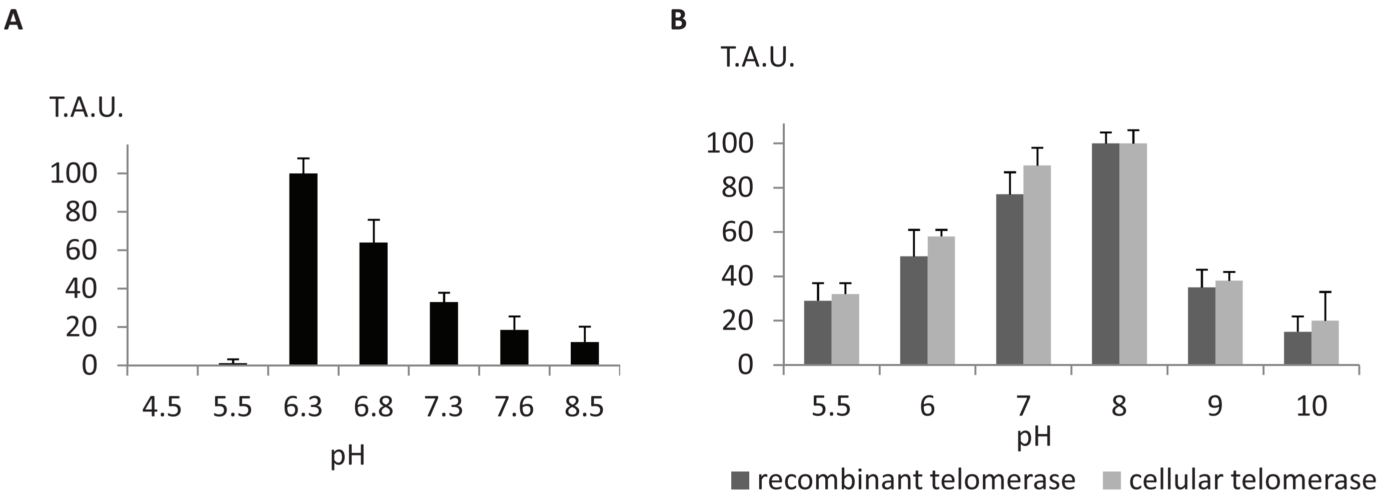

C

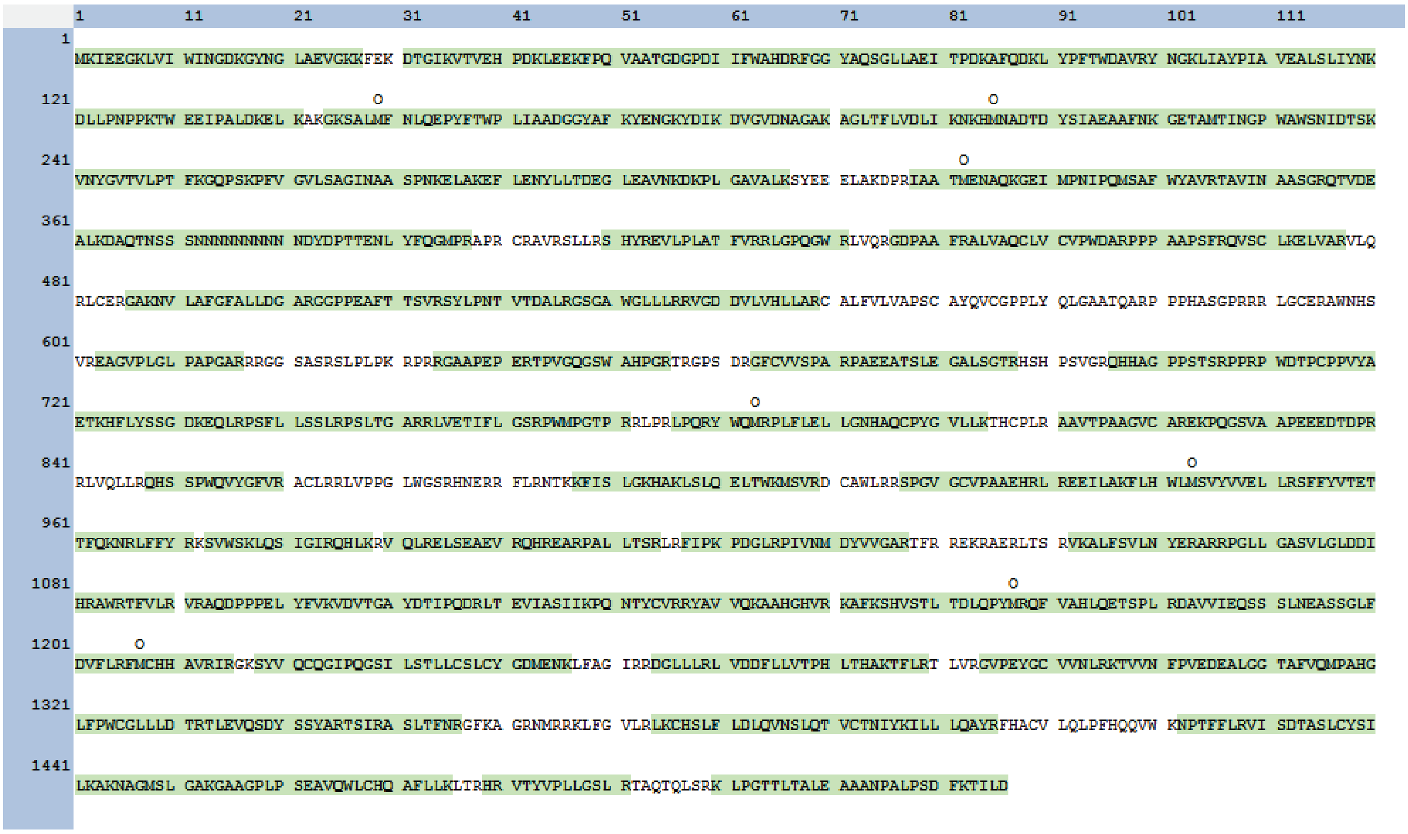

Fig. S3. Purification of MBP-hTERT. (A) Yeast expressing MBP-hTERT (Fig 1A, construct 12) were lysed and the pH of the extract was adjusted with Tris or sodium acetate before column binding. All the washing steps were performed at the corresponding pH for each condition. hTERT was eluted at pH 7.0 and telomerase activity was reconstituted with *in vitro* transcribed hTR and measured by RT-TRAP. Telomerase activity is expressed in arbitrary units (T.A.U.). (B) Recombinant telomerase as well as telomerase extracted from cancer cells were compared for telomerase activity at different pH. The enzymes were diluted 30-fold to decrease the initial buffer strength, and incubated 30 min at 37°C in 10 mM Hepes (range from pH 5.5 to 7) or 10mM Tris (range from pH 8 to 10) buffers, with the addition of 10 µM TS primer, 2 mM $MgCl_2$ and 0.5µM dNTP. Telomerase was inactivated 10 min at 80°C, then the samples were diluted 20-fold in the PCR buffer avec tested for telomerase activity using the RT-TRAP PCR conditions. The buffers pH 5.5 or 10 diluted following the previous scheme did not affected an independent TRAP assay (data not shown) (C) Validation of the identity of the purified protein by proteomic analysis. The purified MBP-hTERT protein (Fig. 1A, construct 12) was digested with trypsin, and the peptides were analyzed by nano-LC-MS/MS. The sequence coverage (showed in green) obtained at 1 % false discovery rate was near 84 %. The list of the peptides detected from MBP-hTERT, and the name of all the other proteins detected in the sample are shown in Tables S1 and S2.

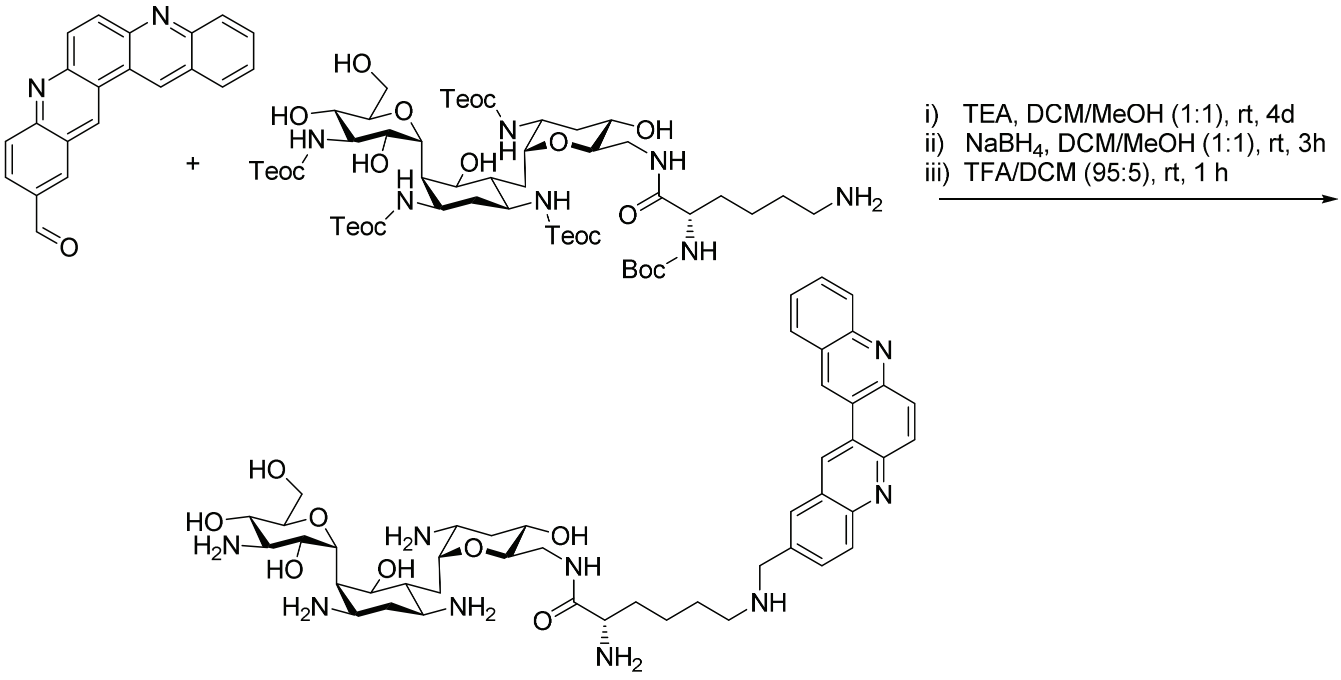

TAI1

Fig. S4. Scheme of synthesis of TAI1.

A

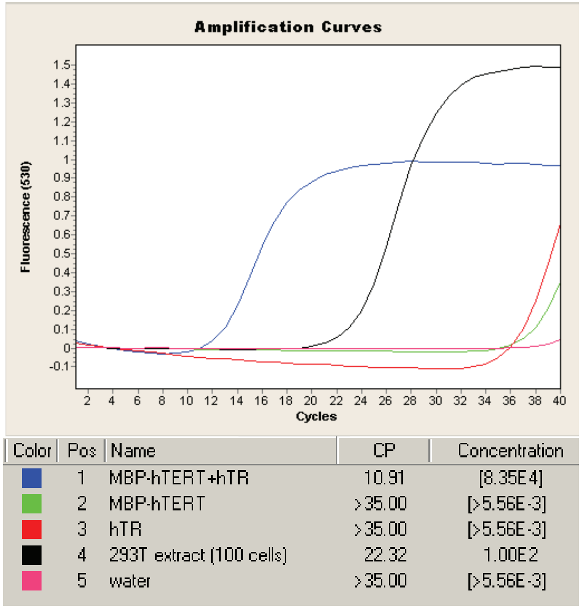

B

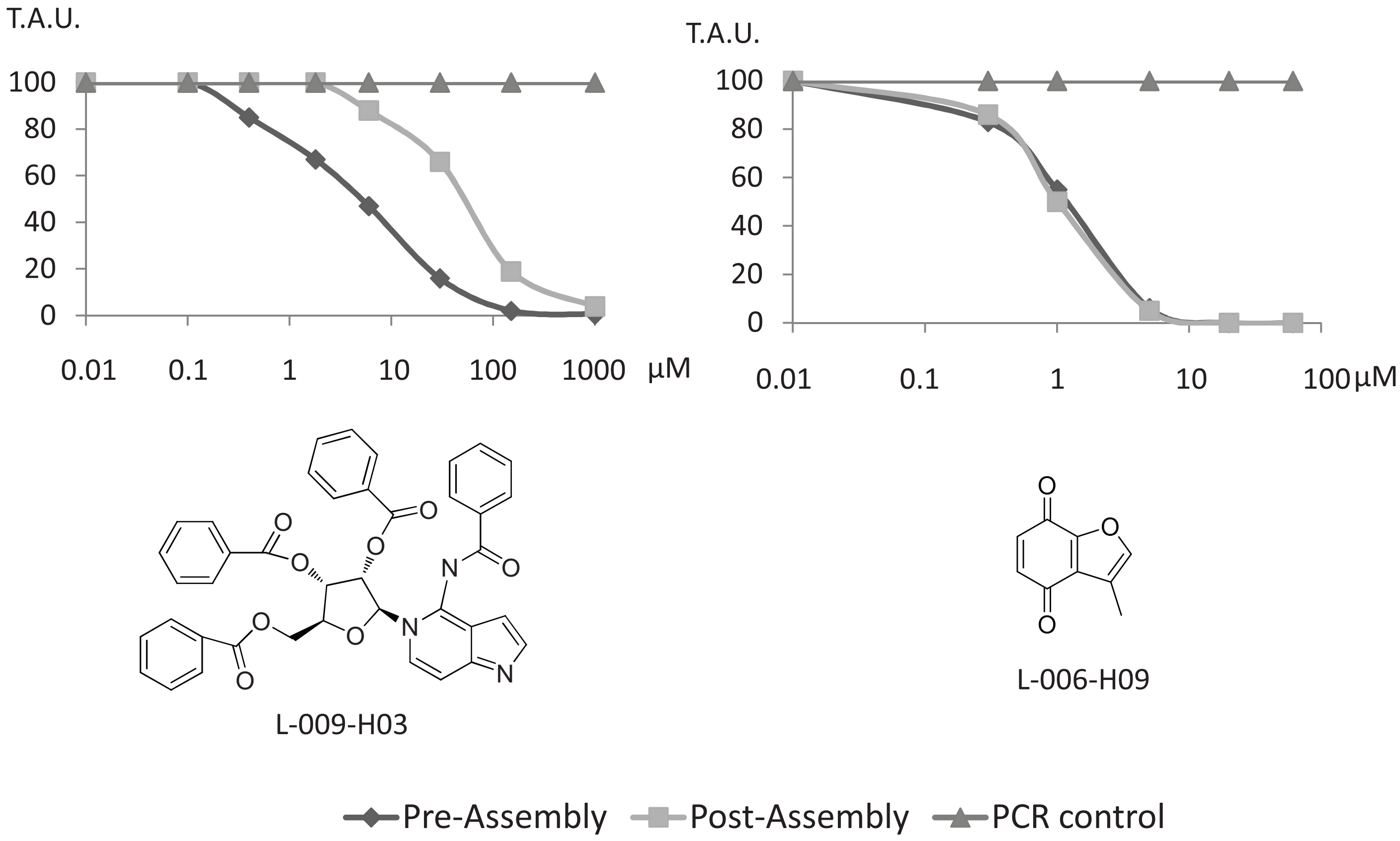

Fig. S5. Telomerase reconstitution and inhibition. (A) Telomerase activity level of the reconstituted telomerase (300 nM) is 83,500 fold higher than the one measured by RT-TRAP in one 293T cell. Crossing-points (CP) were determined using the second derivative maximum method (LightCycler® software). (B) The effects of two others hits from the screen on telomerase assembly and activity has been determined by RT-TRAP. Telomerase activity is expressed in arbitrary units (T.A.U.).

Identification of human telomerase assembly inhibitors enabled by a novel method to produce hTERT

Table S1: Peptides from MBP-hTERT detected by LC-MS/MS

Peptides from MBP

#	Spec. %	Score	Mr(Obs)	Mr(Exp)	Mr(calc)	Delta	Miss	Sequence
1	100	5.4	937.02	1872.02	1872.02	-0.00	2	-.MKIEEGKLVIWINGDK.G
2	100	5.8	716.14	2860.52	2860.52	0.00	3	-.MKIEEGKLVIWINGDKGYNGLAEVGK.K
3	100	5.3	598.73	2988.61	2988.61	0.00	4	-.MKIEEGKLVIWINGDKGYNGLAEVGKK.F
4	100	4.3	807.45	1612.88	1612.88	-0.00	1	K.IEEGKLVIWINGDK.G
5	100	5.5	868.13	2601.38	2601.38	0.00	2	K.IEEGKLVIWINGDKGYNGLAEVGK.K
6	100	2.1	529.31	1056.60	1056.60	0.00	0	K.LVIWINGDK.G
7	100	5.5	1023.55	2045.09	2045.09	-0.00	1	K.LVIWINGDKGYNGLAEVGK.K
8	100	3.4	725.40	2173.19	2173.19	-0.00	2	K.LVIWINGDKGYNGLAEVGKK.F
9	100	2.8	504.26	1006.51	1006.51	-0.00	0	K.GYNGLAEVGK.K
10	100	2.8	568.31	1134.60	1134.60	0.00	1	K.GYNGLAEVGKK.F
11	100	2.0	533.30	1064.59	1064.59	-0.00	2	K.KFEKDTGIK.V
12	100	5.0	781.41	2341.22	2341.22	-0.00	3	K.FEKDTGIKVTVEHPDKLEEK.F
13	100	3.4	719.88	1437.75	1437.75	-0.00	1	K.DTGIKVTVEHPDK.L
14	100	4.6	969.51	1937.01	1937.01	-0.00	2	K.DTGIKVTVEHPDKLEEK.F
15	100	3.0	712.38	1422.74	1422.74	-0.00	1	K.VTVEHPDKLEEK.F
16	100	6.7	905.21	3616.79	3616.80	-0.00	2	K.VTVEHPDKLEEKFPQVAATGDGPDIIFWAHDR.F
17	100	3.3	892.85	4459.21	4459.21	-0.00	2	K.LEEKFPQVAATGDGPDIIFWAHDRFGGYAQSGLLAEITPDK.A
18	100	5.4	1107.04	2212.07	2212.07	0.00	0	K.FPQVAATGDGPDIIFWAHDR.F
19	100	7.4	990.99	3959.95	3959.95	-0.00	1	K.FPQVAATGDGPDIIFWAHDRFGGYAQSGLLAEITPDK.A
20	100	5.8	910.85	4549.23	4549.23	-0.00	2	K.FPQVAATGDGPDIIFWAHDRFGGYAQSGLLAEITPDKAFQDK.L
21	100	5.1	883.95	1765.89	1765.89	0.00	0	R.FGGYAQSGLLAEITPDK.A
22	100	4.1	786.07	2355.17	2355.17	-0.00	1	R.FGGYAQSGLLAEITPDKAFQDK.L
23	100	6.5	901.96	3603.80	3603.80	-0.00	2	R.FGGYAQSGLLAEITPDKAFQDKLYPFTWDAVR.Y
24	100	3.4	814.21	4066.03	4066.03	-0.00	3	R.FGGYAQSGLLAEITPDKAFQDKLYPFTWDAVRYNGK.L
25	100	4.3	928.97	1855.93	1855.93	-0.00	1	K.AFQDKLYPFTWDAVR.Y
26	100	6.1	1048.56	4190.23	4190.22	0.00	3	K.AFQDKLYPFTWDAVRYNGKLIAYPIAVEALSLIYNK.D
27	100	2.1	634.33	1266.64	1266.64	-0.00	0	K.LYPFTWDAVR.Y
28	100	3.6	896.10	4475.44	4475.43	0.01	3	K.LYPFTWDAVRYNGKLIAYPIAVEALSLIYNKDLLPNPPK.T
29	100	3.7	785.11	2352.31	2352.31	0.00	1	R.YNGKLIAYPIAVEALSLIYNK.D
30	100	5.9	807.71	3226.80	3226.80	0.00	2	R.YNGKLIAYPIAVEALSLIYNKDLLPNPPK.T
31	100	6.1	1103.36	4409.39	4409.39	-0.00	3	R.YNGKLIAYPIAVEALSLIYNKDLLPNPPKTWEEIPALDK.E
32	100	4.5	956.93	4779.61	4779.61	-0.00	4	R.YNGKLIAYPIAVEALSLIYNKDLLPNPPKTWEEIPALDKELK.A
33	100	4.6	946.05	1890.09	1890.09	0.00	0	K.LIAYPIAVEALSLIYNK.D
34	100	3.5	692.15	2764.58	2764.58	0.00	1	K.LIAYPIAVEALSLIYNKDLLPNPPK.T
35	100	2.4	790.44	3947.17	3947.17	-0.00	2	K.LIAYPIAVEALSLIYNKDLLPNPPKTWEEIPALDK.E
36	100	4.0	720.57	4317.39	4317.39	0.00	3	K.LIAYPIAVEALSLIYNKDLLPNPPKTWEEIPALDKELK.A
37	100	3.2	601.31	1200.60	1200.60	-0.00	0	K.TWEEIPALDK.E
38	100	3.2	786.42	1570.82	1570.82	-0.00	1	K.TWEEIPALDKELK.A
39	100	2.0	590.99	1769.96	1769.96	-0.00	2	K.TWEEIPALDKELKAK.G
40	100	6.3	1045.86	3134.56	3134.56	0.00	1	K.GKSALMFNLQEPYFTWPLIAADGGYAFK.Y
41	100	6.4	984.15	2949.44	2949.44	0.00	0	K.SALMFNLQEPYFTWPLIAADGGYAFK.Y

42	100	5.4	1181.24	3540.71	3540.71	-0.00	1	K.SALMFNLQEPYFTWPLIAADGGYAFKYENGK.Y
43	100	4.6	1016.00	4059.97	4059.98	-0.00	2	K.SALMFNLQEPYFTWPLIAADGGYAFKYENGKYDIK.D
44	100	5.6	1020.00	4075.97	4075.97	-0.00	2	K.SALMFNLQEPYFTWPLIAADGGYAFKYENGKYDIK.D + Oxidation (M:4)
45	100	2.1	565.28	1128.54	1128.55	-0.00	1	K.YENGKYDIK.D
46	100	5.0	1028.50	2054.99	2054.99	0.00	2	K.YENGKYDIKDVGVDNAGAK.A
47	100	3.9	732.87	1463.73	1463.73	0.00	1	K.YDIKDVGVDNAGAK.A
48	100	3.8	595.36	1188.71	1188.71	0.00	0	K.AGLTFLVDLIK.N
49	100	4.3	716.43	1430.85	1430.85	0.00	1	K.AGLTFLVDLIKNK.H
50	100	4.9	828.42	3309.67	3309.67	0.00	2	K.AGLTFLVDLIKNKHMNADTDYSIAEAAFNK.G
51	100	5.9	1070.49	2138.97	2138.97	0.00	1	K.NKHMNADTDYSIAEAAFNK.G
52	100	4.0	633.28	1896.83	1896.83	0.00	0	K.HMNADTDYSIAEAAFNK.G
53	100	5.2	957.42	1912.83	1912.83	0.00	0	K.HMNADTDYSIAEAAFNK.G + Oxidation (M:2)
54	100	5.7	1015.21	4056.82	4056.83	-0.00	1	K.HMNADTDYSIAEAAFNKGETAMTINGPWAWSNIDTSK.V
55	100	5.1	1090.01	2178.00	2178.01	-0.00	0	K.GETAMTINGPWAWSNIDTSK.V
56	100	5.2	1166.59	3496.73	3496.73	-0.00	1	K.GETAMTINGPWAWSNIDTSKVNYGVTVLPTFK.G
57	100	3.4	669.38	1336.74	1336.74	-0.00	0	K.VNYGVTVLPTFK.G
58	100	4.6	1070.08	2138.15	2138.15	-0.00	1	K.GQPSKPFVGVLSAGINAASPNK.E
59	100	3.3	860.81	2579.40	2579.41	-0.00	2	K.GQPSKPFVGVLSAGINAASPNKELAK.E
60	100	5.7	846.77	2537.29	2537.29	0.00	1	K.ELAKEFLENYLLTDEGLEAVNK.D
61	100	6.0	699.68	2096.03	2096.03	0.00	0	K.EFLENYLLTDEGLEAVNK.D
62	100	6.0	1030.55	3088.63	3088.63	0.00	2	K.EFLENYLLTDEGLEAVNKDKPLGAVALK.S
63	100	2.5	506.31	1010.61	1010.61	-0.00	1	K.DKPLGAVALK.S
64	100	3.7	538.77	1075.53	1075.53	-0.00	0	R.IAATMENAQK.G
65	100	2.9	546.77	1091.53	1091.53	-0.00	0	R.IAATMENAQK.G + Oxidation (M:5)
66	100	5.6	1056.52	3166.54	3166.54	-0.00	1	R.IAATMENAQKGEIMPNIPQMSAFWYAVR.T
67	100	4.3	1027.77	4107.05	4107.05	-0.00	2	R.IAATMENAQKGEIMPNIPQMSAFWYAVRTAVINAASGR.Q
68	100	4.6	1055.52	2109.02	2109.02	-0.00	0	K.GEIMPNIPQMSAFWYAVR.T
69	100	4.4	763.39	3049.53	3049.53	-0.00	1	K.GEIMPNIPQMSAFWYAVRTAVINAASGR.Q
70	100	3.2	480.27	958.52	958.52	-0.00	0	R.TAVINAASGR.Q
71	100	5.0	922.50	1842.98	1842.98	-0.00	1	R.TAVINAASGRQTVDEALK.D
72	100	5.1	970.15	3876.59	3876.59	-0.00	0	K.DAQTNSSSNNNNNNNNNNDYDPTTENLYFQGMPR.A

Peptides from hTERT

#	Spec. %	Score	Mr(Obs)	Mr(Exp)	Mr(calc)	Delta	Miss	Sequence
73	100	3.9	844.47	1686.92	1686.92	-0.00	1	R.SHYREVLPLATFVR.R
74	100	2.6	572.84	1143.67	1143.67	0.00	0	R.EVLPLATFVR.R
75	100	1.9	485.27	968.53	968.53	-0.00	1	R.RLGPQGWR.L
76	100	4.1	820.42	3277.67	3277.66	0.00	2	R.GDPAAFRALVAQCLVCVPWDARPPPAAPSFR.Q
77	100	5.5	855.45	2563.32	2563.32	-0.00	1	R.ALVAQCLVCVPWDARPPPAAPSFR.Q
78	100	2.5	623.35	1244.69	1244.69	0.00	1	R.QVSCLKELVAR.V
79	100	4.5	860.48	1718.95	1718.95	0.00	1	R.GAKNVLAFGFALLDGAR.G
80	100	5.0	730.64	2918.54	2918.54	-0.00	2	R.GAKNVLAFGFALLDGARGGPPEAFTTSVR.S
81	100	4.4	732.40	1462.79	1462.79	0.00	0	K.NVLAFGFALLDGAR.G
82	100	3.2	609.81	1217.60	1217.60	0.00	0	R.GGPPEAFTTSVR.S
83	100	2.9	675.36	1348.70	1348.70	0.00	0	R.SYLPNTVTDALR.G
84	100	3.8	787.43	2359.27	2359.26	0.00	1	R.SYLPNTVTDALRGSGAWGLLLR.R
85	100	2.5	839.46	2515.37	2515.37	0.00	2	R.SYLPNTVTDALRGSGAWGLLLRR.V
86	100	3.1	515.30	1028.58	1028.58	-0.00	0	R.GSGAWGLLLR.R
87	100	2.4	593.35	1184.68	1184.68	-0.00	1	R.GSGAWGLLLRR.V
88	100	4.2	488.29	1461.84	1461.84	-0.00	1	R.RVGDDVLVHLLAR.C
89	100	3.2	653.88	1305.74	1305.74	0.00	0	R.VGDDVLVHLLAR.C
90	100	3.1	652.87	1303.72	1303.72	0.00	0	R.EAGVPLGLPAPGAR.R
91	100	2.1	532.34	1062.67	1062.67	-0.00	2	R.SLPLPKRPR.R
92	100	2.2	491.76	981.50	981.50	-0.00	1	R.RGAAPEPER.T
93	100	4.1	771.72	2312.15	2312.15	-0.00	2	R.RGAAPEPERTPVGQGSWAHPGR.T
94	100	4.2	719.69	2156.05	2156.05	-0.00	1	R.GAAPEPERTPVGQGSWAHPGR.T
95	100	3.7	675.34	1348.66	1348.66	0.00	0	R.TPVGQGSWAHPGR.T
96	100	3.6	835.75	2504.23	2504.23	-0.00	1	R.GFCVVSPARPAEEATSLEGALSGTR.H
97	100	2.9	1037.17	3108.50	3108.50	0.00	2	R.QHHAGPPSTSRPPRPWDTPCPPVYAETK.H
98	100	2.2	527.25	1052.49	1052.49	-0.00	0	K.HFLYSSGDK.E
99	100	3.2	653.35	3261.73	3261.73	0.00	3	K.HFLYSSGDKEQLRPSFLLSSLRPSLTGAR.R
100	100	4.0	743.42	2227.24	2227.24	-0.00	2	K.EQLRPSFLLSSLRPSLTGAR.R
101	100	3.4	738.40	2212.19	2212.19	-0.00	2	R.RLVETIFLGSRPWMPGTPR.R
102	100	5.7	686.37	2056.09	2056.09	0.00	1	R.LVETIFLGSRPWMPGTPR.R
103	100	2.2	554.06	2212.19	2212.19	0.00	2	R.LVETIFLGSRPWMPGTPRR.L
104	100	5.5	871.72	3482.85	3482.85	-0.00	2	R.LPQRYWQMRPLFLELLGNHAQCPYGVLLK.T
105	100	5.9	748.14	2988.55	2988.55	-0.00	1	R.YWQMRPLFLELLGNHAQCPYGVLLK.T
106	100	4.6	752.14	3004.55	3004.55	0.00	1	R.YWQMRPLFLELLGNHAQCPYGVLLK.T + Oxidation (M:4)
107	100	2.6	543.79	1085.56	1085.57	-0.00	0	R.AAVTPAAGVCAR.E
108	100	3.3	977.95	1953.89	1953.89	-0.00	1	R.EKPQGSVAAPEEEDTDPR.R
109	100	3.1	704.34	2109.99	2109.99	-0.00	2	R.EKPQGSVAAPEEEDTDPRR.L
110	100	2.9	530.93	1589.77	1589.77	-0.00	0	R.QHSSPWQVYGFVR.A
111	100	2.2	619.36	1236.71	1236.71	0.00	1	R.RLVPPGLWGSR.H
112	100	2.5	564.85	1127.68	1127.68	0.00	2	K.KFISLGKHAK.L
113	100	1.8	500.80	999.59	999.59	-0.00	1	K.FISLGKHAK.L
114	100	4.0	485.28	1452.81	1452.81	-0.00	1	K.HAKLSLQELTWK.M
115	100	3.0	559.32	1116.62	1116.62	-0.00	0	K.LSLQELTWK.M
116	100	2.3	530.96	1589.86	1589.86	-0.00	1	K.LSLQELTWKMSVR.D
117	100	2.1	718.37	1434.72	1434.72	0.00	1	R.RSPGVGCVPAAEHR.L

118	100	3.9	640.32	1278.62	1278.61	0.00	0	R.SPGVGCVPAAEHR.L
119	100	2.1	486.30	970.58	970.58	-0.00	1	R.LREEILAK.F
120	100	6.3	953.21	2856.61	2856.61	-0.00	2	R.LREEILAKFLHWLMSVYVVELLR.S
121	100	3.6	719.16	2872.60	2872.60	0.00	2	R.LREEILAKFLHWLMSVYVVELLR.S + Oxidation (M:14)
122	100	7.0	1084.84	4335.31	4335.32	-0.00	3	R.LREEILAKFLHWLMSVYVVELLRSFFYVTETTFQK.N
123	100	4.5	846.69	3382.75	3382.75	0.00	1	K.FLHWLMSVYVVELLRSFFYVTETTFQK.N
124	100	3.6	749.37	1496.72	1496.72	-0.00	0	R.SFFYVTETTFQK.N
125	100	2.4	508.28	1014.54	1014.54	-0.00	1	K.NRLFFYR.K
126	100	3.5	687.40	1372.78	1372.78	-0.00	1	K.SVWSKLQSIGIR.Q
127	100	2.8	646.89	1291.77	1291.77	-0.00	1	K.LQSIGIRQHLK.R
128	100	1.9	528.97	1583.87	1583.87	-0.00	2	K.RVQLRELSEAEVR.Q
129	100	3.4	476.93	1427.77	1427.77	0.00	1	R.VQLRELSEAEVR.Q
130	100	2.9	617.34	1848.99	1848.99	0.00	2	R.VQLRELSEAEVRQHR.E
131	100	1.9	677.35	1352.68	1352.68	0.00	1	R.ELSEAEVRQHR.E
132	100	3.3	612.83	2447.30	2447.30	-0.00	3	R.ELSEAEVRQHREARPALLTSR.L
133	100	2.6	557.32	1112.63	1112.63	-0.00	1	R.EARPALLTSR.L
134	100	3.3	590.08	2356.27	2356.27	0.00	2	R.FIPKPDGLRPIVNMDYVVGAR.T
135	100	3.4	719.91	1437.80	1437.80	0.00	1	R.VKALFSVLNYER.A
136	100	3.6	606.33	1210.63	1210.63	0.00	0	K.ALFSVLNYER.A
137	100	2.2	719.89	1437.77	1437.77	-0.00	1	K.ALFSVLNYERAR.R
138	100	4.8	504.79	2015.14	2015.14	0.00	2	R.ARRPGLLGASVLGLDDIHR.A
139	100	5.6	597.01	1788.00	1788.00	-0.00	1	R.RPGLLGASVLGLDDIHR.A
140	100	4.4	551.31	2201.22	2201.22	-0.00	2	R.RPGLLGASVLGLDDIHRAWR.T
141	100	4.0	705.40	2817.59	2817.59	-0.00	3	R.RPGLLGASVLGLDDIHRAWRTFVLR.V
142	100	3.2	829.95	1657.88	1657.88	-0.00	1	R.VRAQDPPPELYFVK.V
143	100	2.5	702.36	1402.71	1402.71	-0.00	0	R.AQDPPPELYFVK.V
144	100	4.2	978.82	2933.45	2933.44	0.00	1	R.AQDPPPELYFVKVDVTGAYDTIPQDR.L
145	100	3.1	775.38	1548.74	1548.74	-0.00	0	K.VDVTGAYDTIPQDR.L
146	100	5.5	1193.62	3577.84	3577.85	-0.00	2	K.VDVTGAYDTIPQDRLTEVIASIIKPQNTYCVR.R
147	100	3.8	1024.56	2047.11	2047.11	-0.00	1	R.LTEVIASIIKPQNTYCVR.R
148	100	2.9	735.41	2203.21	2203.21	-0.00	2	R.LTEVIASIIKPQNTYCVRR.Y
149	100	4.3	718.40	1434.78	1434.78	-0.00	1	R.YAVVQKAAHGHVR.K
150	100	4.9	708.04	2121.10	2121.10	-0.00	2	R.KAFKSHVSTLTDLQPYMR.Q
151	100	4.4	824.41	1646.81	1646.81	0.00	0	K.SHVSTLTDLQPYMR.Q
152	100	3.4	832.41	1662.80	1662.80	0.00	0	K.SHVSTLTDLQPYMR.Q + Oxidation (M:13)
153	100	3.0	763.41	1524.81	1524.80	0.00	0	R.QFVAHLQETSPLR.D
154	100	2.8	1023.28	4089.08	4089.08	0.00	1	R.QFVAHLQETSPLRDAVVIEQSSSLNEASSGLFDVFLR.F
155	100	5.9	861.77	2582.29	2582.29	0.00	0	R.DAVVIEQSSSLNEASSGLFDVFLR.F
156	100	4.4	891.94	3563.73	3563.73	0.00	1	R.DAVVIEQSSSLNEASSGLFDVFLRFMCHHAVR.I
157	100	2.9	767.59	3832.92	3832.91	0.00	2	R.DAVVIEQSSSLNEASSGLFDVFLRFMCHHAVRIR.G
158	100	2.5	500.73	999.45	999.45	0.00	0	R.FMCHHAVR.I
159	100	2.5	508.73	1015.45	1015.45	0.00	0	R.FMCHHAVR.I + Oxidation (M:2)
160	100	5.8	1050.82	3149.45	3149.45	-0.00	0	K.SYVQCQGIPQGSILSTLLCSLCYGDMENK.L
161	100	5.5	829.48	2485.41	2485.41	0.00	1	R.DGLLLRLVDDFLLVTPHLTHAK.T
162	100	4.2	751.68	3002.71	3002.71	0.00	2	R.DGLLLRLVDDFLLVTPHLTHAKTFLR.T
163	100	4.0	910.01	1818.00	1818.00	-0.00	0	R.LVDDFLLVTPHLTHAK.T
164	100	2.6	653.34	1304.66	1304.65	0.00	0	R.GVPEYGCVVNLR.K
165	100	3.1	717.38	1432.75	1432.75	0.00	1	R.GVPEYGCVVNLRK.T
166	100	4.4	1008.02	4028.03	4028.03	0.00	1	R.KTVVNFPVEDEALGGTAFVQMPAHGLFPWCGLLLDTR.T
167	100	4.2	975.99	3899.94	3899.94	0.00	0	K.TVVNFPVEDEALGGTAFVQMPAHGLFPWCGLLLDTR.T

168	100	3.5	759.86	1517.70	1517.70	0.00	0	R.TLEVQSDYSSYAR.T
169	100	2.7	633.35	1264.69	1264.69	-0.00	1	R.TSIRASLTFNR.G
170	100	2.0	570.81	1139.61	1139.61	0.00	1	R.ASLTFNRGFK.A
171	100	2.6	751.01	3750.04	3750.04	0.00	2	R.LKCHSLFLDLQVNSLQTVCTNIYKILLLQAYR.F
172	100	2.3	495.31	988.61	988.61	-0.00	0	K.ILLLQAYR.F
173	100	5.4	796.76	2387.26	2387.26	0.00	1	K.NPTFFLRVISDTASLCYSILK.A
174	100	5.8	863.14	2586.39	2586.39	-0.00	2	K.NPTFFLRVISDTASLCYSILKAK.N
175	100	5.5	854.96	3415.80	3415.80	0.00	3	K.NPTFFLRVISDTASLCYSILKAKNAGMSLGAK.G
176	100	4.3	756.90	1511.79	1511.79	0.00	0	R.VISDTASLCYSILK.A
177	100	3.4	856.47	1710.92	1710.92	0.00	1	R.VISDTASLCYSILKAK.N
178	100	2.8	524.28	1046.55	1046.55	0.00	1	K.AKNAGMSLGAK.G
179	100	3.5	1168.61	2335.21	2335.21	-0.00	0	K.GAAGPLPSEAVQWLCHQAFLLK.L
180	100	2.5	902.82	2705.45	2705.45	0.00	1	K.GAAGPLPSEAVQWLCHQAFLLKLTR.H
181	100	3.5	755.95	1509.88	1509.88	0.00	1	R.HRVTYVPLLGSLR.T
182	100	3.2	609.37	1216.72	1216.72	-0.00	0	R.VTYVPLLGSLR.T
183	100	3.0	776.09	2325.26	2325.26	-0.00	1	R.KLPGTTLTALEAAANPALPSDFK.T
184	100	3.9	923.51	2767.50	2767.50	-0.00	1	R.KLPGTTLTALEAAANPALPSDFKTILD.-

Table S2: proteins co-detected with MBP-hTERT

822/984 Proteins	Gene name	MW kDa	Peptides	Cov. %	known hTERT-interacting protein	Description - Species
**MBP-hTERT**	**MBP-hTERT**	**170.4**	**150**	**82.5**		**Maltose Binding Protein (*E. coli*) and Telomerase reverse transcriptase (*Homo sapiens*) fusion protein**
F2QYW1_PICP7	HSA1	69.6	45	65.3	Forsythe et al., J Biol Chem. 2001 May 11;276(19):15571-4.	Heat shock 70kDa protein 1/8 Pichia pastoris
F2QTY0_PICP7	EEF2	212.6	40	24	Li et al., J Biol Chem. 1998 Dec 11;273(50):33436-42.	Classical protein kinase C Pichia pastoris
F2QN77_PICP7	PP7435_Chr1-0897	80.9	38	59.6	Holt et al., Genes Dev. 1999 Apr 1;13(7):817-26.	Heat shock cognate protein HSP 90-beta Pichia pastoris
F2QX14_PICP7	PP7435_Chr3-0996	70.8	34	60.3	Forsythe et al., J Biol Chem. 2001 May 11;276(19):15571-4.	Heat shock 70 kDa protein Pichia pastoris
F2QRW8_PICP7	agl	173.9	32	23.6		4-alpha-glucanotransferase Pichia pastoris
F2QU09_PICP7	PAB1	70.1	26	48.2		Polyadenylate-binding protein, cytoplasmic and nuclear Pichia pastoris
F2QTT5_PICP7	glgP	97.7	23	29		Phosphorylase Pichia pastoris
F2QQI4_PICP7	PP7435_Chr1-1551	44.6	21	62.2		Small heat shock protein, chloroplastic Pichia pastoris
F2QMR6_PICP7	PP7435_Chr1-0728	123.1	21	23.3		Putative uncharacterized protein Pichia pastoris
F2QVJ7_PICP7	PP7435_Chr3-0464	66.5	20	34.7	Forsythe et al., J Biol Chem. 2001 May 11;276(19):15571-4.	Heat shock 70kDa protein 1/8 Pichia pastoris
F2R049_PICP7	PP7435_Chr4-0875	66.4	20	36.3		ATP-dependent RNA helicase Pichia pastoris
F2QVS4_PICP7	upf1	107.8	19	26.1		Regulator of nonsense transcripts 1 Pichia pastoris
F2QQG8_PICP7	TEF2	50.1	19	44.9		Elongation factor 1-alpha Pichia pastoris

F2QLR2_PICP7	sbe1	80.5	19	29.9		1,4-alpha-glucan branching enzyme Pichia pastoris
F2QM64_PICP7	PP7435_Chr1-0352	121.4	18	23.2		Eukaryotic initiation factor 4F subunit p130 Pichia pastoris
F2R0F1_PICP7	CEF3	116.6	18	19.6		Elongation factor EF-3 Pichia pastoris
F2QLH6_PICP7	PP7435_Chr1-1078	206.1	18	12.1		Putative uncharacterized protein Pichia pastoris
F2QLC7_PICP7	PP7435_Chr1-1029	250.2	18	9.8		Biotin carboxylase Pichia pastoris
F2R033_PICP7	CPNA	73.9	17	36.2		Heat shock protein 60 Pichia pastoris
F2R050_PICP7	RPL3	43.7	17	47.9		60S ribosomal protein L3 Pichia pastoris
F2QQU0_PICP7	PP7435_Chr2-0071	43.4	17	45		Chaperone protein DnaJ 1 Pichia pastoris
F2QR18_PICP7	URA2	245.0	17	9.4		Carbamoyl-phosphate synthase (Glutamine hydrolyzing), aspartate carbamoyltransferase Ura1 (EC:2.1.3.2 2.4.2.-3.5.2.3 6.3.5.5) Pichia pastoris
F2QNM8_PICP7	FAS1	229.7	17	10.5		Fatty acid synthase subunit beta, fungi type Pichia pastoris
ARO1_PICPG	ARO1	170.1	16	13.2		Pentafunctional AROM polypeptide Pichia pastoris
F2QXG9_PICP7	ENO2	46.5	14	41.5		Enolase Pichia pastoris
F2QNB7_PICP7	CFT1	155.9	14	13.4		Protein cft1 Pichia pastoris
F2QMJ7_PICP7	RPL1	24.5	13	45.2		Ribosomal protein Pichia pastoris
F2QTX9_PICP7	PP7435_Chr2-1181	139.6	13	13		DNA-directed RNA polymerase Pichia pastoris
C4R506_PICPG	PAS_chr3_0595	45.0	13	32.3		Translation initiation factor eIF4A, identical to Tif1p Pichia pastoris
F2QZT6_PICP7	RPS4	31.8	13	37.9		40S ribosomal protein S4 Pichia pastoris
F2QZ17_PICP7	PP7435_Chr4-0479	69.5	12	24.8		Putative uncharacterized protein Pichia pastoris
F2QZQ1_PICP7	PP7435_Chr4-0723	58.0	12	32.4		Nucleolar protein NOP58 Pichia pastoris
F2QVV6_PICP7	CRP1	32.2	12	40.2		N-acetyltransferase eco Pichia pastoris
F2QVX2_PICP7	SRP1	61.2	12	31.4		Importin subunit alpha Pichia pastoris
F2QLH3_PICP7	PGK1	44.1	12	39.7		Phosphoglycerate kinase Pichia pastoris
F2QWW3_PICP7	PGD	54.0	12	32.7		6-phosphogluconate dehydrogenase, decarboxylating Pichia pastoris
F2QRS4_PICP7	POR1	29.6	12	51.2		Voltage-dependent anion-selective channel protein 3 Pichia pastoris
RS3A_PICPG	RPS1	28.9	12	57.4		40S ribosomal protein S1 Pichia pastoris

F2QNT9_PICP7	PP7435_Chr1-0531	26.8	12	46.1		60S ribosomal protein L8-B Pichia pastoris
F2QPD9_PICP7	PP7435_Chr1-1137	41.4	11	36.8		60S ribosomal protein L18 Pichia pastoris
F2QVE6_PICP7	GYS2	79.8	11	20.9		Glycogen(Starch) synthase Pichia pastoris
F2QLR1_PICP7	PP7435_Chr1-0028	36.9	11	34.8		60S ribosomal protein L4-B Pichia pastoris
C4QV56_PICPG	PAS_chr1-3_0074	54.0	11	29.5		Cytoplasmic ATP-dependent RNA helicase of the DEAD-box family Pichia pastoris
F2QRZ6_PICP7	PP7435_Chr2-0486	17.7	11	56.2		40S ribosomal protein S18 Pichia pastoris
C4QYC8_PICPG	PAS_chr1-4_0403	55.5	11	27.4		Essential evolutionarily-conserved nucleolar protein component of the box C/D snoRNP complexes Pichia pastoris
F2QV67_PICP7	MAS1	50.9	11	28.3		Mitochondrial processing peptidase Pichia pastoris
F2QT12_PICP7	TDH1	35.6	10	42.6		Glyceraldehyde-3-phosphate dehydrogenase Pichia pastoris
F2QNH1_PICP7	PSE1	123.9	10	13.1		Importin subunit beta-3 Pichia pastoris
F2QRI7_PICP7	COQ6	51.0	10	27.9		Ubiquinone biosynthesis monooxygenase Coq6 Pichia pastoris
F2R0B0_PICP7	PP7435_Chr4-0937	45.2	10	27.6		Moesin/ezrin/radixin homolog 1 Pichia pastoris
F2QS13_PICP7	PYK2	55.6	10	27.7		Pyruvate kinase Pichia pastoris
F2QY29_PICP7	COQ1	54.3	10	26.8		Hexaprenyl pyrophosphate synthetase,mitochondrial Pichia pastoris
F2QPZ0_PICP7	Chc	187.3	10	8.2		Clathrin heavy chain 1 Pichia pastoris
F2QRT3_PICP7	PP7435_Chr2-0421	119.5	10	10.7		60 kDa chaperonin 3 Pichia pastoris
F2QLZ7_PICP7	PP7435_Chr1-0118	26.9	10	39.9		40S ribosomal protein S3 Pichia pastoris
F2QQ33_PICP7	RPS2	28.2	9	40.7		40S ribosomal protein S2 Pichia pastoris
F2QLM3_PICP7	PP7435_Chr1-0981	47.4	9	21.6		Midasin Pichia pastoris
F2QRQ6_PICP7	PP7435_Chr2-0391	67.5	9	21.5		Similar to outer mitochondrial membrane translocase Pichia pastoris
C4QWG6_PICPG	PAS_chr1-1_0219	27.2	9	34.6		Protein component of the large (60S) ribosomal subunit Pichia pastoris
F2QYB8_PICP7	PP7435_Chr4-0221	22.8	9	41.6		Uncharacterized protein MRP8 Pichia pastoris
F2QV49_PICP7	VIG9	39.9	9	31.9		Mannose-1-phosphate guanylyltransferase Pichia pastoris

F2QVG5_PICP7	RPS5	24.6	9	48.9		40S ribosomal protein S5 Pichia pastoris
F2R058_PICP7	RPL6	18.6	9	53.9		60S ribosomal protein L6 Pichia pastoris
F2QW22_PICP7	TUF1	46.8	9	29.9		Elongation factor Tu Pichia pastoris
F2QTU9_PICP7	metE	85.8	9	13.2		5-methyltetrahydropteroyltriglutamate--homocysteine methyltransferase Pichia pastoris
F2QXR5_PICP7	PP7435_Chr4-0013	13.2	9	67.6		40S ribosomal protein S10 Pichia pastoris
C4R1F0_PICPG	PAS_chr2-1_0677	115.0	9	10.7		Karyopherin, a carrier protein involved in nuclear import of proteins Pichia pastoris
F2QSG4_PICP7	GFA1	77.4	9	14.8		Glucosamine--fructose-6-phosphate aminotransferase (Isomerizing) Pichia pastoris
F2QWP9_PICP7	RPG1	97.4	9	12.9		Eukaryotic translation initiation factor 3 subunit A Pichia pastoris
C4QYP5_PICPG	PAS_chr1-4_0517	96.0	9	13.2		Subunit of both RNase MRP, which cleaves pre-rRNA, and nuclear RNase P Pichia pastoris
F2QSA1_PICP7	PP7435_Chr2-0592	51.3	9	19.3	Venteicher AS, et al., Cell. 2008 Mar 21;132(6):945-57.	RuvB-like protein 2 Pichia pastoris
F2QUG8_PICP7	APR1	44.3	8	25.1		Vacuolar aspartic proteinase similar to S. cerevisiae PEP4 (YPL154C) Pichia pastoris
F2QSX6_PICP7	ADH2	37.0	8	31.4		Alcohol dehydrogenase Pichia pastoris
F2QU82_PICP7	PP7435_Chr2-1286	27.6	8	34.2		60S ribosomal protein L7 Pichia pastoris
F2QRA9_PICP7	PP7435_Chr2-0241	45.7	8	22.9		Peptidyl-prolyl cis-trans isomerase Pichia pastoris
F2QSW8_PICP7	PP7435_Chr2-0813	15.2	8	54.9		40S ribosomal protein S14 Pichia pastoris
F2QVI0_PICP7	PP7435_Chr3-0446	61.3	8	17.6		Eukaryotic translation initiation factor 3 subunit D Pichia pastoris
F2QW09_PICP7	atpA	58.8	8	19.4		ATP synthase subunit alpha Pichia pastoris
F2QXS9_PICP7	PP7435_Chr4-0027	91.1	8	11.9		Transitional endoplasmic reticulum ATPase Pichia pastoris
F2QWM4_PICP7	SSC1	69.7	8	17		Molecular chaperone DnaK Pichia pastoris

C4R903_PICPG	PAS_chr4_0808	33.6	8	36.1		Nucleolar protein, component of the small subunit processome complex, which is required for processi Pichia pastoris
F2QZA5_PICP7	PP7435_Chr4-0571	16.4	8	59.2		40S ribosomal protein S16 Pichia pastoris
C4R5U7_PICPG	PAS_chr3_0876	42.4	8	21.9		S-adenosylmethionine synthetase Pichia pastoris
F2QXX2_PICP7	SLA3	118.1	8	9.6		Huntingtin-interacting protein 1 Pichia pastoris
F2QV66_PICP7	UBA1	115.2	8	10.2		Ubiquitin-activating enzyme E1 Pichia pastoris
F2QUN5_PICP7	NRP1	70.7	8	15.6		Uncharacterized RNA-binding protein C17H9.04c Pichia pastoris
F2QU21_PICP7	PP7435_Chr2-1224	17.9	8	40.5		60S ribosomal protein L24-A Pichia pastoris
F2QR48_PICP7	NAP1	48.9	7	25		Putative nucleosome assembly protein C2D10.11C Pichia pastoris
F2QSD2_PICP7	PP7435_Chr2-0623	24.2	7	35.3		Ribosomal protein L15 Pichia pastoris
F2QT51_PICP7	PFK1	108.8	7	9.8		6-phosphofructokinase Pichia pastoris
F2QS58_PICP7	PP7435_Chr2-0549	16.5	7	55.7		60S ribosomal protein L27a-3 Pichia pastoris
F2QMU3_PICP7	rps8	22.5	7	42.5		40S ribosomal protein S8 Pichia pastoris
F2QNU8_PICP7	PRB2	59.1	7	14.8		Cerevisin Pichia pastoris
F2QRL2_PICP7	PP7435_Chr2-0346	12.0	7	44.4		40S ribosomal protein S25 Pichia pastoris
F2QTW5_PICP7	bip2	74.2	7	13		78 kDa glucose-regulated protein homolog Pichia pastoris
F2QLU5_PICP7	RPP0	33.7	7	26.9		60S acidic ribosomal protein P0 Pichia pastoris
F2QQW5_PICP7	PP7435_Chr2-0096	79.2	7	14.2		Pumilio homolog 2 Pichia pastoris
F2QY88_PICP7	PP7435_Chr4-0191	92.3	7	11.1		Protein PUF2 Pichia pastoris
F2QYT2_PICP7	PP7435_Chr4-0392	63.5	7	13.7		Putative uncharacterized protein Pichia pastoris
F2QZ39_PICP7	PP7435_Chr4-0502	49.9	7	18.9	Venteicher AS, et al., Cell. 2008 Mar 21;132(6):945-57.	RuvB-like helicase 1 Pichia pastoris
F2QXK2_PICP7	PP7435_Chr3-1188	21.5	7	34.8		60S ribosomal protein L20 Pichia pastoris
F2QWI5_PICP7	acsA	74.1	7	13.1		Acetyl-CoA synthetase Pichia pastoris
F2QP67_PICP7	PP7435_Chr1-0662	34.1	7	30.3		60S ribosomal protein L5-B Pichia pastoris
F2QRU8_PICP7	PP7435_Chr2-0436	133.6	7	6.5		DIS3-like exonuclease 2 Pichia pastoris

F2QP07_PICP7	Rrm1	97.5	7	9.9		Ribonucleoside-diphosphate reductase Pichia pastoris
F2QVA4_PICP7	MAS2	52.7	7	16.2		Mitochondrial processing peptidase Pichia pastoris
F2QRI5_PICP7	-	18.2	6	55		Zinc finger protein GIS2 Pichia pastoris
F2QVK8_PICP7	PP7435_Chr3-0476	17.2	6	54		Ubiquitin-like protein Pichia pastoris
COQ4_PICPG	COQ4	35.1	6	30.4		Ubiquinone biosynthesis protein COQ4, mitochondrial Pichia pastoris
F2QZY4_PICP7	PP7435_Chr4-0808	58.6	6	15.5		ATP-dependent RNA helicase DDX5/DBP2 Pichia pastoris
F2QU20_PICP7	PP7435_Chr2-1223	11.6	6	50.5		60S ribosomal protein L30 Pichia pastoris
F2QLY3_PICP7	clpB	100.3	6	8.4		Chaperone protein clpB Pichia pastoris
ACT_PICPG	ACT1	41.7	6	25.8		Actin Pichia pastoris
F2QR41_PICP7	atpD	54.0	6	15.9		ATP synthase subunit beta Pichia pastoris
F2R000_PICP7	NSR1	38.8	6	23.5		Nuclear localization sequence-binding protein Pichia pastoris
F2QQJ5_PICP7	SSE1	78.7	6	9.8		Heat shock 70 kDa protein 1 Pichia pastoris
F2QYJ3_PICP7	PP7435_Chr4-0299	19.9	6	31		60S ribosomal protein L11 Pichia pastoris
F2R087_PICP7	CAM1	47.1	6	15.6		Elongation factor EF-1 gamma subunit Pichia pastoris
F2QQA2_PICP7	CIC1	46.4	6	19.5		Proteasome-interacting protein CIC1 Pichia pastoris
F2QN05_PICP7	PFK1	103.8	6	8.4		6-phosphofructokinase Pichia pastoris
F2QMX2_PICP7	PP7435_Chr1-0787	115.9	6	7.8		Putative transcription initiation factor TFIID 111 kDa subunit Pichia pastoris
F2QWL8_PICP7	ERG6	43.3	6	19.3		Sterol 24-C-methyltransferase Pichia pastoris
F2QZX6_PICP7	PP7435_Chr4-0800	22.7	6	24.6		60S ribosomal protein L16-B Pichia pastoris
F2QVF3_PICP7	NIP1	92.5	6	8.6		Eukaryotic translation initiation factor 3 subunit C Pichia pastoris
F2QQS5_PICP7	PP7435_Chr2-0056	25.5	6	28.8		60S ribosomal protein L10 Pichia pastoris
F2QU02_PICP7	BMH1	29.0	6	33.5	Seimiya H, et al. EMBO J. 2000 Jun 1;19(11):2652-61.	14-3-3 protein homolog Pichia pastoris
F2QS01_PICP7	PP7435_Chr2-0491	15.7	6	44.1		40S ribosomal protein S17-A Pichia pastoris
F2QZA6_PICP7	PP7435_Chr4-0572	22.2	6	34.5		60S ribosomal protein L13 Pichia pastoris
F2R084_PICP7	PP7435_Chr4-0911	72.9	6	12.4		Nucleolar GTP-binding protein 1 Pichia pastoris

F2QR25_PICP7	SIS1	37.0	6	23.1		Chaperone protein dnaJ Pichia pastoris
F2QUW6_PICP7	kars	67.5	6	11.9		Lysyl-tRNA synthetase, class II Pichia pastoris
C4R2J6_PICPG	PAS_c034_0035	11.4	6	58.3		Histone H4 Pichia pastoris
F2QQ37_PICP7	PP7435_Chr1-1400	85.7	6	8.6		Pre-mRNA-splicing factor ATP-dependent RNA helicase DHX16 Pichia pastoris
C4R5B4_PICPG	PAS_chr3_0701	82.1	6	10.1		Subunit of the core complex of translation initiation factor 3(EIF3), essential for translation Pichia pastoris
F2QS72_PICP7	PP7435_Chr2-0563	33.7	6	26		Peroxisomal biogenesis factor 19 Pichia pastoris
F2QRA8_PICP7	RPL6	17.4	6	42.4		60S ribosomal protein L6 Pichia pastoris
F2QSL1_PICP7	PP7435_Chr2-0703	17.6	6	39.4		40S ribosomal protein S11 Pichia pastoris
F2QST1_PICP7	STI1	63.5	6	12.8		Heat shock protein sti1 homolog Pichia pastoris
F2QR23_PICP7	CBF5	54.5	6	15.1	Venteicher AS, et al., Cell. 2008 Mar 21;132(6):945-57.	H/ACA ribonucleoprotein complex subunit 4 Pichia pastoris (Dyskerin)
C4QXL7_PICPG	PAS_chr1-4_0160	92.1	6	8.4		Multifunctional enzyme HIS4 Pichia pastoris
F2QZ43_PICP7	AVL9	91.6	6	9.3		Late secretory pathway protein AVL9 Pichia pastoris
F2QQ91_PICP7	VPS1	76.6	6	10.8		Dynamin-related protein 3B Pichia pastoris
F2QLM9_PICP7	PP7435_Chr1-0988	69.1	6	11.7		Signal recognition particle subunit SRP72 Pichia pastoris
F2QQY3_PICP7	PP7435_Chr2-0114	19.1	6	27.6		60S ribosomal protein L17 Pichia pastoris
F2QPL4_PICP7	PP7435_Chr1-1214	20.9	6	34.6		60S ribosomal protein L21-B Pichia pastoris
F2QM24_PICP7	PP7435_Chr1-0146	14.5	5	38		60S ribosomal protein L23 Pichia pastoris
F2QXG1_PICP7	PP7435_Chr3-1146	23.6	5	24		60S ribosomal protein L19 Pichia pastoris
F2QW14_PICP7	PP7435_Chr3-0635	57.1	5	13.9		T-complex protein 1 subunit delta Pichia pastoris
F2QUZ8_PICP7	PP7435_Chr3-0255	67.4	5	11.8		ATP-binding cassette sub-family F member 2 Pichia pastoris
F2QN25_PICP7	AHA1	37.5	5	21.6		Activator of 90 kDa heat shock protein ATPase homolog 1 Pichia pastoris
F2QYT1_PICP7	IDH1	39.6	5	20		Isocitrate dehydrogenase (NAD+) Pichia pastoris
F2R095_PICP7	RS6	27.0	5	27.5		40S ribosomal protein S6 Pichia pastoris
F2QPL3_PICP7	PP7435_Chr1-1213	22.2	5	27.5		40S ribosomal protein S9-A Pichia pastoris

RSSA_PICPG	RPS0	29.2	5	26.6		40S ribosomal protein S0 Pichia pastoris
F2QLT2_PICP7	TPS2	95.2	5	7.9		Alpha,alpha-trehalose-phosphate synthase (UDP-forming) Pichia pastoris
F2QRB8_PICP7	-	82.5	5	10		Threonyl-tRNA synthetase Pichia pastoris
F2QRP6_PICP7	PP7435_Chr2-0381	119.6	5	6.7		UPF0202 protein YNL132W Pichia pastoris
F2QQP0_PICP7	PP7435_Chr2-0019	129.3	5	5.7		Pyruvate carboxylase Pichia pastoris
F2QXR6_PICP7	RPS6	27.3	5	27.3		40S ribosomal protein S6 Pichia pastoris
F2QZN6_PICP7	PP7435_Chr4-0707	15.4	5	35.6		40S ribosomal protein S24 Pichia pastoris
F2QM84_PICP7	FBA1	39.7	5	19.4		Fructose-bisphosphate aldolase variant 1, class II Pichia pastoris
F2QZ21_PICP7	TPD3	71.9	5	11.5	Xi et al. J Cell Biochem. 2013 Feb;114(2):409-17.	Serine/threonine-protein phosphatase 2A 65 kDa regulatory subunit A alpha isoform , subunit A, PR65-alpha isoform; , subunit A, R1-alpha isoform Pichia pastoris
F2QSD4_PICP7	PP7435_Chr2-0625	106.3	5	8.7		26S proteasome non-ATPase regulatory subunit 1 Pichia pastoris
F2QTZ2_PICP7	PP7435_Chr2-1194	60.4	5	13.5		T-complex protein 1 subunit alpha Pichia pastoris
F2QMN9_PICP7	RPL9A	21.7	5	24.1		60S ribosomal protein L9-A Pichia pastoris
F2QLF3_PICP7	PP7435_Chr1-1055	12.1	5	41.1		60S ribosomal protein L33-A Pichia pastoris
F2QM69_PICP7	NUG1	55.5	5	12.2		GTP-binding protein engA Pichia pastoris
F2QWL4_PICP7	GARS	74.2	5	9.8		Glycyl-tRNA synthetase Pichia pastoris
C4QYP7_PICPG	PAS_chr1-4_0519	59.3	5	12.8		Co-chaperone that stimulates the ATPase activity of Ssa1p Pichia pastoris
F2QVZ9_PICP7	PP7435_Chr3-0620	15.6	5	50.7		40S ribosomal protein S12 Pichia pastoris
F2QXV9_PICP7	NMD2	110.1	5	6.4		Nonsense-mediated mRNA decay protein 2 Pichia pastoris
F2QRR4_PICP7	PAT1	94.5	5	8.2		DNA topoisomerase 2-associated protein PAT1 Pichia pastoris
F2QNJ7_PICP7	PP7435_Chr1-0269	17.6	5	35.4		Peptidyl-prolyl cis-trans isomerase Pichia pastoris
F2QP29_PICP7	PP7435_Chr1-0622	13.9	5	29.2		60S ribosomal protein L35 Pichia pastoris
F2QP63_PICP7	PP7435_Chr1-0658	9.7	5	28.6		60S ribosomal protein L37 Pichia pastoris

F2QYZ3_PICP7	gua1	56.1	4	12.5		Inosine-5'-monophosphate dehydrogenase Pichia pastoris
F2QP20_PICP7	PP7435_Chr1-0613	136.7	4	4.8		Coatomer subunit alpha Pichia pastoris
F2QY41_PICP7	cprA	77.5	4	10.8		NADPH--cytochrome P450 reductase Pichia pastoris
F2QMK4_PICP7	PP7435_Chr1-0499	17.8	4	32.1		60S ribosomal protein L12 Pichia pastoris
F2QYT7_PICP7	PP7435_Chr4-0397	75.7	4	8.5		DNA repair protein RAD5 Pichia pastoris
F2QWU7_PICP7	LTV1	48.8	4	14.9		Helicase domino Pichia pastoris
F2QS18_PICP7	COQ5	32.8	4	18		Ubiquinone biosynthesis methyltransferase COQ5 Pichia pastoris
F2QXL1_PICP7	TOM1	374.1	4	1.5		E3 ubiquitin-protein ligase HUWE1 Pichia pastoris
F2QUG4_PICP7	PP7435_Chr3-0068	66.2	4	7		Putative uncharacterized protein Pichia pastoris
F2QMP8_PICP7	PP7435_Chr1-0710	76.3	4	9.3		Prolyl-tRNA synthetase Pichia pastoris
F2QPQ6_PICP7	SES1	52.9	4	11.5		Seryl-tRNA synthetase Pichia pastoris
F2QQZ5_PICP7	PP7435_Chr2-0126	44.2	4	12.5		Eukaryotic translation initiation factor 3 subunit M Pichia pastoris
C4R868_PICPG	PAS_chr4_0533	48.8	4	10.8		Beta-tubulin Pichia pastoris
F2QMB9_PICP7	GDH1	49.3	4	12.5		Glutamate dehydrogenase Pichia pastoris
F2QWM8_PICP7	PP7435_Chr3-0854	30.6	4	21.6		Mitochondrial peculiar membrane protein 1 Pichia pastoris
F2QUA4_PICP7	PP7435_Chr3-0008	14.9	4	30.1		60S ribosomal protein L14-B Pichia pastoris
F2QLH1_PICP7	PP7435_Chr1-1073	35.0	4	16.3		Eukaryotic translation initiation factor 3 subunit F Pichia pastoris
F2QX59_PICP7	PDC1	61.4	4	10		Pyruvate decarboxylase Pichia pastoris
F2QLE7_PICP7	PP7435_Chr1-1049	100.2	4	6.1		Replication factor C subunit 1 Pichia pastoris
F2QRS1_PICP7	nuc1	183.7	4	3.1		DNA-directed RNA polymerase Pichia pastoris
F2QP72_PICP7	PP7435_Chr1-0669	51.2	4	12		Protein SLF1 Pichia pastoris
C4R0T8_PICPG	PAS_chr2-1_0482	14.6	4	34.6		Protein component of the small (40S) ribosomal subunit Pichia pastoris
F2QYC2_PICP7	Rpt1	49.5	4	11.3		26S protease regulatory subunit 7 Pichia pastoris
F2QSD3_PICP7	SER3	51.0	4	11.3		D-3-phosphoglycerate dehydrogenase Pichia pastoris

C4QXU7_PICPG	-	7.6	4	58.2		Protein component of the small (40S) ribosomal subunit Pichia pastoris
F2QU73_PICP7	PP7435_Chr2-1277	56.2	4	10		Putative uncharacterized protein Pichia pastoris
F2QLA9_PICP7	PP7435_Chr1-0016	103.5	4	5.3		Transportin-1 Pichia pastoris
F2QNM0_PICP7	PMA1	97.9	4	5.6		H+-transporting ATPase Pichia pastoris
F2QQR8_PICP7	PP7435_Chr2-0049	52.4	4	11.5		Dihydrolipoyl dehydrogenase Pichia pastoris
C4R0F8_PICPG	PAS_chr2-1_0362	13.7	4	33.6		Non-essential regulatory subunit B of protein phosphatase 2A, which has multiple roles in mitosis an Pichia pastoris
F2QZW6_PICP7	YPT1	24.2	4	25.2		Ras-like GTP-binding protein YPT1 Pichia pastoris
F2QX17_PICP7	SLB2	33.9	4	16.6		Sphingolipid long chain base-responsive protein PIL1 Pichia pastoris
F2QNL4_PICP7	TIF34	37.6	4	14.6		Uncharacterized WD repeat-containing protein alr3466 Pichia pastoris
F2QSF4_PICP7	GLC7	36.9	4	14.1		Serine/threonine-protein phosphatase Pichia pastoris
F2QNC9_PICP7	RPN8	37.7	4	16.4		26S proteasome regulatory subunit RPN8 Pichia pastoris
F2QUJ2_PICP7	RPN6	49.8	4	10.6		Uncharacterized protein F59B2.5 Pichia pastoris
F2QZX4_PICP7	PP7435_Chr4-0798	21.3	4	14.4		40S ribosomal protein S7-B Pichia pastoris
F2QS15_PICP7	acsB	73.9	4	6.4		Acetyl-CoA synthetase Pichia pastoris
F2QNV7_PICP7	RPL9	21.7	4	16.8		50S ribosomal protein L6 Pichia pastoris
F2QY23_PICP7	PP7435_Chr4-0126	37.8	4	14.6		DNA-directed RNA Polymerase III subunit C5 Pichia pastoris
F2QQ36_PICP7	ABC1	66.5	4	6.3		Chaperone activity of bc1 complex-like,mitochondrial Pichia pastoris
F2QT71_PICP7	NMD3	57.5	4	9.1		60S ribosomal export protein NMD3 Pichia pastoris
F2QX48_PICP7	NAB3	77.7	4	7.6		Nuclear polyadenylated RNA-binding protein 3 Pichia pastoris
C4R5E1_PICPG	PAS_chr3_0728	127.8	4	4.2		ATP binding cassette family member Pichia pastoris
F2QQF5_PICP7	LAC9	112.5	4	5.8		Lactose regulatory protein LAC9 Pichia pastoris

C4R7G6_PICPG	PAS_chr4_0302	162.2	4	3.7		DNA-directed RNA polymerase Pichia pastoris
F2QTN7_PICP7	PP7435_Chr2-1088	48.5	4	11.6		37S ribosomal protein S23, mitochondrial Pichia pastoris
F2QUV8_PICP7	CP51	58.9	4	10.1		Cytochrome P450, family 51 (Sterol 14-demethylase) Pichia pastoris
F2QPW2_PICP7	PP7435_Chr1-1321	78.7	4	6.4		Protein SQS1 Pichia pastoris
F2QUU9_PICP7	GAD1	62.8	3	11.7		Glutamate decarboxylase Pichia pastoris
CBPY_PICPG	PRC1	59.4	3	8.6		Carboxypeptidase Y Pichia pastoris
F2R040_PICP7	HOM3	57.1	3	9.2		Aspartokinase Pichia pastoris
F2QYD1_PICP7	MAK5	85.2	3	5.8		Similar to ATP-dependent RNA helicase Pichia pastoris
F2QWK7_PICP7	TIM9	10.4	3	43.7		Mitochondrial import inner membrane translocase subunit TIM9 Pichia pastoris
F2QUS3_PICP7	RPF2	36.8	3	11.3		Ribosome biogenesis protein RPF2 Pichia pastoris
C4R1D1_PICPG	PAS_chr2-1_0660	63.3	3	7.8		Enzyme of 'de novo' purine biosynthesis Pichia pastoris
F2QWT8_PICP7	PP7435_Chr3-0918	60.6	3	7.7		Thermosome subunit Pichia pastoris
F2QUL4_PICP7	IF5A	17.0	3	19.4		Eukaryotic translation initiation factor 5A Pichia pastoris
F2QXA8_PICP7	SEC4	22.8	3	20.6		Ras-related protein RIC1 Pichia pastoris
C4QZ92_PICPG	PAS_FragB_0036	11.5	3	23.8		60S ribosomal protein L36 Pichia pastoris
F2QWR7_PICP7	PP7435_Chr3-0893	149.4	3	3.4		DNA topoisomerase 2 Pichia pastoris
F2QR51_PICP7	PRS5	45.6	3	10.5		Ribose-phosphate pyrophosphokinase Pichia pastoris
C4QYM0_PICPG	PAS_chr1-4_0490	12.2	3	23.7		60S acidic ribosomal protein P2-A Pichia pastoris
F2QYT6_PICP7	PP7435_Chr4-0396	76.3	3	6.4		Probable E3 ubiquitin-protein ligase MGRN1 Pichia pastoris
F2QS79_PICP7	SKI2	140.3	3	3.3		Antiviral helicase SKI2 Pichia pastoris
F2QNB3_PICP7	PP7435_Chr1-0933	69.8	3	7.3		Putative uncharacterized protein Pichia pastoris
F2QPK2_PICP7	PP7435_Chr1-1202	9.0	3	32.1		60S ribosomal protein L38 Pichia pastoris
F2QXZ5_PICP7	PP7435_Chr4-0097	33.1	3	16.1		Putative uncharacterized protein Pichia pastoris

F2QZI0_PICP7	RPB3	34.2	3	14.1		DNA-directed RNA polymerase II subunit RPB3 .5 Pichia pastoris
F2QPA7_PICP7	PP7435_Chr1-1105	134.6	3	3.5		RRP12-like protein Pichia pastoris
C4QZA3_PICPG	PAS_FragB_0044	49.8	3	11.8		Transcriptional coactivator HFI1/ADA1 Pichia pastoris
F2QVT2_PICP7	THI4	37.1	3	11.6		Thiazole biosynthetic enzyme, mitochondrial Pichia pastoris
F2QMV6_PICP7	PP7435_Chr1-0771	47.7	3	9.2		Polyadenylate-binding protein 1 Pichia pastoris
F2QZY7_PICP7	PP7435_Chr4-0811	45.9	3	6.8		Ribonucleoside-diphosphate reductase subunit M2 Pichia pastoris
F2QZR3_PICP7	PP7435_Chr4-0735	14.3	3	24.4		50S ribosomal protein L24P Pichia pastoris
C4R760_PICPG	PAS_chr4_0210	33.2	3	8.2		Major ADP/ATP carrier of the mitochondrial inner membrane Pichia pastoris
F2QQ12_PICP7	SLB2	35.2	3	13.1		Probable sphingolipid long chain base-responsive protein C3C7.02c .02c Pichia pastoris
C4QWE9_PICPG	PAS_chr1-1_0204	32.0	3	14.8		Putative uncharacterized protein Pichia pastoris
F2QPA4_PICP7	PP7435_Chr1-1102	114.8	3	4.4		Uncharacterized protein YPL009C Pichia pastoris
F2QRK9_PICP7	MDJ1	53.1	3	6.1		Chaperone protein dnaJ Pichia pastoris
F2QM36_PICP7	PP7435_Chr1-0158	59.9	3	9.6		Uncharacterized RNA-binding protein YPL184C Pichia pastoris
F2QY34_PICP7	PP7435_Chr4-0137	15.7	3	28.2		60S ribosomal protein L25-B Pichia pastoris
F2QSB0_PICP7	trp2	76.0	3	4.7		Tryptophan synthase alpha chain Pichia pastoris
F2QN95_PICP7	INT6	49.2	3	10.1		Eukaryotic translation initiation factor 3 subunit E Pichia pastoris
F2QSS9_PICP7	SNF1	62.7	3	6.9		Carbon catabolite-derepressing protein kinase Pichia pastoris
F2QYD2_PICP7	etf1	48.8	3	9.6		Eukaryotic peptide chain release factor subunit 1 Pichia pastoris
F2QXM1_PICP7	PP7435_Chr3-1208	51.3	3	9.5		Cysteine desulfurase Pichia pastoris
F2QUK8_PICP7	PP7435_Chr3-0114	44.1	3	9.5		Probable RNA-binding protein sce3 Pichia pastoris
F2QTN1_PICP7	PP7435_Chr2-1082	8.5	3	54.2		DNA-directed RNA Polymerase II subunit L Pichia pastoris
C4R783_PICPG	PAS_chr4_0228	73.0	3	5.7		Acetolactate synthase Pichia pastoris

F2QY52_PICP7	NHP2	16.8	3	27.9		H/ACA ribonucleoprotein complex subunit 2 Pichia pastoris
F2QQT0_PICP7	YNK1	17.0	3	25		Nucleoside-diphosphate kinase Pichia pastoris
F2QZ03_PICP7	CCT6	58.3	3	7.6		T-complex protein 1 subunit zeta Pichia pastoris
F2R0C3_PICP7	SCL1	27.6	3	13.7		20S proteasome subunit alpha 1 Pichia pastoris
F2QLX4_PICP7	KGD2	48.1	3	9.3		2-oxoglutarate dehydrogenase E2 component (Dihydrolipoamide succinyltransferase) Pichia pastoris
F2QPJ6_PICP7	COQ9	32.5	3	13.6		Ubiquinone biosynthesis protein COQ9,mitochondrial Pichia pastoris
F2QT01_PICP7	GSP1	24.1	3	12.7		GTP-binding nuclear protein GSP1/Ran Pichia pastoris
F2QQN5_PICP7	PP7435_Chr2-0014	50.1	3	8.7		Uncharacterized protein YBL086C Pichia pastoris
F2R062_PICP7	RNT1	59.4	3	7.3		Ribonuclease III Pichia pastoris
C4QYR8_PICPG	PAS_chr1-4_0688	37.2	3	11.7		Putative uncharacterized protein Pichia pastoris
GET3_PICPG	GET3	38.8	3	10.2		ATPase GET3 Pichia pastoris
C4R8T4_PICPG	-	7.2	3	39.3		Constituent of small nucleolar ribonucleoprotein particles containing H/ACA-type snoRNAs Pichia pastoris
F2QZQ4_PICP7	FRS2	56.7	3	7.1		Phenylalanyl-tRNA synthetase alpha chain Pichia pastoris
F2QM65_PICP7	PP7435_Chr1-0353	44.1	3	9.3		26S protease regulatory subunit 8 Pichia pastoris
F2QNG3_PICP7	RTN1	32.6	3	13.1		Reticulon-like protein 1 Pichia pastoris
F2QW11_PICP7	PP7435_Chr3-0632	39.3	3	12.5		DNA-directed RNA polymerase III subunit C4 Pichia pastoris
F2QZV9_PICP7	PP7435_Chr4-0783	100.9	3	4.2		Coatomer subunit gamma Pichia pastoris
F2QR13_PICP7	CYS4	54.9	3	8.2		Cystathionine beta-synthase Pichia pastoris
C4R3W9_PICPG	PAS_chr3_0223	53.9	3	4.9		Non-ATPase regulatory subunit of the 26S proteasome lid Pichia pastoris
F2QS12_PICP7	URK1	54.6	3	7.4		Uridine kinase Pichia pastoris
F2QMP7_PICP7	PP7435_Chr1-0709	61.4	3	6.7		Asparaginyl-tRNA synthetase Pichia pastoris
F2QQC1_PICP7	PP7435_Chr1-1486	49.6	3	9.1		Protein VTS1 Pichia pastoris

F2R006_PICP7	PP7435_Chr4-0831	13.6	3	29.9		60S ribosomal protein L31 Pichia pastoris
F2QR44_PICP7	PP7435_Chr2-0175	40.1	3	11.1		Homoisocitrate dehydrogenase, mitochondrial Pichia pastoris
F2QNX4_PICP7	PP7435_Chr1-0566	115.5	3	3.9		Translation initiation factor IF-2 Pichia pastoris
F2QQ44_PICP7	PP7435_Chr1-1407	82.4	3	4.6		Glutamyl-tRNA synthetase Pichia pastoris
F2QU81_PICP7	PP7435_Chr2-1285	17.3	3	21.1		UPF0368 protein Cxorf26 Pichia pastoris
F2QPT5_PICP7	PP7435_Chr1-1294	16.3	3	25.7		30S ribosomal protein S19P Pichia pastoris
C4R396_PICPG	PAS_c131_0021	55.4	3	7.2		Vacuolar ATP synthase subunit B Pichia pastoris
C4R7V7_PICPG	PAS_chr4_0431	223.4	3	2.2		Component of the CCR4-NOT complex, which has multiple roles in regulating mRNA levels Pichia pastoris
F2QTL7_PICP7	MDH1	34.9	3	11.4		Malate dehydrogenase Pichia pastoris
F2QME4_PICP7	PP7435_Chr1-0438	21.8	3	13		Putative uncharacterized protein Pichia pastoris
F2QVI7_PICP7	MEU1	34.1	3	11.8		Purine nucleoside phosphorylase Pichia pastoris
F2QZZ1_PICP7	DCP2	100.8	3	4.3		mRNA-decapping enzyme subunit 2 Pichia pastoris
F2QSU8_PICP7	-	17.7	3	20.1		Putative uncharacterized protein Pichia pastoris
F2QM72_PICP7	PP7435_Chr1-0360	60.9	3	6		ATP-dependent RNA helicase DDX18 Pichia pastoris
F2QVF7_PICP7	PP7435_Chr3-0423	84.4	3	4.7		Phosphoribosylamine--glycine ligase / phosphoribosylformylglycinamidine cyclo-ligase Pichia pastoris
F2QZA4_PICP7	SHM2	52.1	3	6.6		Glycine hydroxymethyltransferase Pichia pastoris
F2QMI5_PICP7	FRQ1	22.1	3	17.4		Frequenin-1 Pichia pastoris
F2QZC6_PICP7	-	125.4	3	3.5		Leucyl-tRNA synthetase Pichia pastoris
F2QWY4_PICP7	ADK1	28.0	3	13.5		Adenylate kinase Pichia pastoris
C4R0N8_PICPG	PAS_chr2-1_0892	40.6	3	9.7		Putative uncharacterized protein Pichia pastoris
F2QP58_PICP7	-	36.6	3	10.5		KRR1 small subunit processome component Pichia pastoris
F2QV35_PICP7	tbpA	46.4	3	8.7		26S protease regulatory subunit 6B homolog Pichia pastoris
F2QNA7_PICP7	CLC1	24.9	3	17.1		Midasin Pichia pastoris

F2QYC8_PICP7	PP7435_Chr4-0231	48.6	3	8		26S protease regulatory subunit 4 homolog Pichia pastoris
F2QZ60_PICP7	PP7435_Chr4-0524	17.1	3	16.6		40S ribosomal protein S13 Pichia pastoris
F2QUC5_PICP7	PP7435_Chr3-0029	75.3	3	5.6		Histone-lysine N-methyltransferase, H3 lysine-79 specific Pichia pastoris
F2QZE6_PICP7	PP7435_Chr4-0614	26.8	3	15.7		MKI67 FHA domain-interacting nucleolar phosphoprotein-like Pichia pastoris
F2QZV7_PICP7	OLA1	44.0	3	7.6		GTP-dependent nucleic acid-binding protein engD Pichia pastoris
F2QYQ8_PICP7	PP7435_Chr4-0368	28.9	2	12.1		Mitochondrial acidic protein mam33 Pichia pastoris
F2QLB6_PICP7	TRP1	26.2	2	13.5		Phosphoribosylanthranilate isomerase Pichia pastoris
F2QRK6_PICP7	ABP1	60.5	2	5.5		Actin-binding protein Pichia pastoris
F2QSZ4_PICP7	PP7435_Chr2-0840	79.5	2	4		NADH dehydrogenase (Ubiquinone) Fe-S protein 1 Pichia pastoris
C4R315_PICPG	PAS_chr2-2_0045	202.7	2	1.6		Nucleolar protein, component of the small subunit (SSU) processome containing the U3 snoRNA Pichia pastoris
F2QMI4_PICP7	CAT5	26.0	2	12.7		Ubiquinone biosynthesis monooxygenase Coq7 Pichia pastoris
F2QV18_PICP7	PP7435_Chr3-0275	102.1	2	3.5		26S proteasome regulatory subunit rpn1 Pichia pastoris
C4R0H3_PICPG	PAS_chr2-1_0376	23.3	2	11.8		Putative GTPase, member of the Obg family Pichia pastoris
F2QLH8_PICP7	PP7435_Chr1-1080	29.7	2	11.1		Suppressor protein STM1 Pichia pastoris
F2QUW7_PICP7	PP7435_Chr3-0223	38.3	2	7.7		Eukaryotic translation initiation factor 3 subunit H Pichia pastoris
F2QS28_PICP7	EGD2	21.7	2	16.3		Nascent polypeptide-associated complex subunit alpha Pichia pastoris
F2QYH3_PICP7	PP7435_Chr4-0276	10.6	2	26.2		60S acidic ribosomal protein P1 Pichia pastoris
C4R2E3_PICPG	PAS_chr2-2_0247	25.9	2	13.1		Beta' regulatory subunit of casein kinase 2, a Ser/Thr protein kinase Pichia pastoris
F2R0H2_PICP7	CYC1	12.1	2	13.6		Cytochrome c Pichia pastoris
F2QZK1_PICP7	PP7435_Chr4-0671	57.0	2	6.2		Signal recognition particle subunit SRP54 Pichia pastoris

F2QTA6_PICP7	MES1	84.2	2	3.8		Methionyl-tRNA synthetase Pichia pastoris
F2QVF2_PICP7	PP7435_Chr3-0418	40.3	2	7.5		Putative uncharacterized protein Pichia pastoris
F2QUL9_PICP7	dis3	108.8	2	3		Exosome complex exonuclease DIS3/RRP44 Pichia pastoris
F2QU85_PICP7	PP7435_Chr2-1289	24.9	2	14.9		Putative uncharacterized protein Pichia pastoris
F2QNF8_PICP7	PHR1	57.3	2	6.1		pH-responsive protein 1 Pichia pastoris
F2QWD7_PICP7	PP7435_Chr3-0761	27.8	2	9.9		20S proteasome subunit alpha 4 Pichia pastoris
F2QX91_PICP7	BRX1	33.7	2	14		Brix domain-containing protein 2 Pichia pastoris
F2QXF2_PICP7	PP7435_Chr3-1137	11.1	2	15.6		60S acidic ribosomal protein P2 Pichia pastoris
C4R1S3_PICPG	PAS_chr2-1_0789	61.6	2	5.2		Subunit of the membrane-associated retromer complex essential for endosome-to-Golgi retrograde prote Pichia pastoris
F2QZL8_PICP7	PP7435_Chr4-0688	172.8	2	2.1		Transcription elongation factor SPT6 Pichia pastoris
F2QMK6_PICP7	ATP5	21.7	2	13.7		F-type H+-transporting ATPase oligomycin sensitivity conferral protein Pichia pastoris
F2QU28_PICP7	PP7435_Chr2-1231	58.8	2	5.3		T-complex protein 1 subunit eta Pichia pastoris
F2QS54_PICP7	PP7435_Chr2-0545	40.4	2	6.8		Putative uncharacterized protein Pichia pastoris
C4R5Z7_PICPG	CLU1	144.6	2	2.2		Putative uncharacterized protein Pichia pastoris
F2QXL9_PICP7	LEU2	39.8	2	7.5		3-isopropylmalate dehydrogenase Pichia pastoris
F2QN63_PICP7	SGTA	36.7	2	7.4		Small glutamine-rich tetratricopeptide repeat-containing protein alpha Pichia pastoris
F2QNN9_PICP7	SUB2	49.2	2	6.2		ATP-dependent RNA helicase UAP56/SUB2 Pichia pastoris
F2QYJ5_PICP7	-	104.9	2	3.3		Fork head protein homolog 2 Pichia pastoris
F2QPQ3_PICP7	FAL1	45.0	2	6.1		DEAD-box protein, putative RNA helicase similar to S. cerevisiae FAL1 (YDR021W) involved in rRNA processing Pichia pastoris

F2QNU1_PICP7	SBP1	38.6	2	8.9		Polyadenylate-binding protein, cytoplasmic and nuclear Pichia pastoris
F2QZY3_PICP7	PP7435_Chr4-0807	15.1	2	20.6		DNA-directed RNA polymerase III subunit C19 Pichia pastoris
F2QUG0_PICP7	PP7435_Chr3-0064	125.4	2	2.6		Zn2+-dependent endopeptidase Pichia pastoris
F2QRR5_PICP7	NOP2	68.3	2	4.9		Putative ribosomal RNA methyltransferase Nop2 Pichia pastoris
F2QX77_PICP7	HCH1	17.1	2	20.1		Uncharacterized protein C1711.08 Pichia pastoris
F2QUU1_PICP7	PP7435_Chr3-0197	23.1	2	10.9		Eukaryotic translation initiation factor 4E Pichia pastoris
F2QWP7_PICP7	NSF	84.0	2	4.2		Vesicle-fusing ATPase Pichia pastoris
F2QRV7_PICP7	PP7435_Chr2-0446	146.6	2	2.4		5-oxoprolinase (ATP-hydrolysing) Pichia pastoris
F2QU18_PICP7	sucA	112.8	2	2.6		2-oxoglutarate dehydrogenase E1 component Pichia pastoris
F2QXF9_PICP7	PP7435_Chr3-1144	112.6	2	2.5		Uncharacterized protein C4F10.09c Pichia pastoris
F2QZT3_PICP7	PP7435_Chr4-0756	43.0	2	7.4		Branched-chain-amino-acid aminotransferase Pichia pastoris
F2QNR0_PICP7	RET2	79.2	2	4.5		Coatomer subunit delta Pichia pastoris
F2QSR3_PICP7	PUF3	84.4	2	3		Pumilio homolog 1 Pichia pastoris
F2QN73_PICP7	PP7435_Chr1-0893	54.0	2	4.6		Histone deacetylase Pichia pastoris
F2QWC4_PICP7	ATP4	25.4	2	11.6		F-type H+-transporting ATPase subunit b Pichia pastoris
F2QTC9_PICP7	clpB	92.8	2	3.2		Chaperone protein clpB Pichia pastoris
F2QV79_PICP7	RAT1	114.0	2	2.5		5'-3' exoribonuclease 2 Pichia pastoris
F2QUB0_PICP7	MRP4	43.0	2	6.3		37S ribosomal protein MRP4, mitochondrial Pichia pastoris
SAR1_PICPG	SAR1	21.7	2	17.4		Small COPII coat GTPase SAR1 Pichia pastoris
F2QTN2_PICP7	NAN1	85.4	2	3.3		NET1-associated nuclear protein 1 Pichia pastoris
F2QRC0_PICP7	PP7435_Chr2-0252	28.5	2	9.5		50S ribosomal protein L3 Pichia pastoris
F2QR95_PICP7	PP7435_Chr2-0227	47.9	2	5.3		Putative uncharacterized protein Pichia pastoris
C4QXC0_PICPG	PAS_chr1-4_0063	34.7	2	10.1		G-protein beta subunit and guanine nucleotide dissociation inhibitor for Gpa2p Pichia pastoris
F2QTE2_PICP7	PP7435_Chr2-0990	38.6	2	7.8		Alcohol dehydrogenase Pichia pastoris

F2QNR4_PICP7	-	70.9	2	4.9		Myb-related protein B Pichia pastoris
F2QZF7_PICP7	PP7435_Chr4-0625	36.7	2	7.8		Cell division control protein 28 Pichia pastoris
F2QN81_PICP7	PP7435_Chr1-0901	65.3	2	4.1		Signal recognition particle 68 kDa protein Pichia pastoris
F2QS71_PICP7	HEM1	61.5	2	4.6		5-aminolevulinate synthase Pichia pastoris
F2QX79_PICP7	COQ3	36.4	2	9.2		Similar to S. cerevisiae COQ3 (YOL096C) 3,4-dihydroxy-5-hexaprenylbenzoate methyltransferase,involved in ubiquinone biosynthesis Pichia pastoris
F2R003_PICP7	PP7435_Chr4-0828	37.8	2	7.8		Meiotically up-regulated gene 64 protein Pichia pastoris
F2QV94_PICP7	PP7435_Chr3-0354	33.4	2	9.1		Succinyl-CoA synthetase alpha subunit Pichia pastoris
C4QVC8_PICPG	PAS_chr1-3_0142	14.2	2	16.4		Lsm (Like Sm) protein Pichia pastoris
F2QXU8_PICP7	PP7435_Chr4-0046	18.4	2	16.1		50S ribosomal protein L13 Pichia pastoris
F2QVH7_PICP7	PP7435_Chr3-0443	88.5	2	2.5		Putative uncharacterized protein Pichia pastoris
F2QVT0_PICP7	PP7435_Chr3-0549	46.6	2	5.8		DNA-directed RNA polymerase I subunit A49 Pichia pastoris
F2QZA8_PICP7	SIP5	54.8	2	4.8		Protein SIP5 Pichia pastoris
F2QNG6_PICP7	PP7435_Chr1-0238	40.3	2	7.2		p38 MAP kinase Pichia pastoris
F2QXI1_PICP7	PP7435_Chr3-1167	66.7	2	4		Stress response protein nst1 Pichia pastoris
C4R4G2_PICPG	PAS_chr3_1188	43.3	2	6.2		Putative uncharacterized protein Pichia pastoris
F2QT63_PICP7	PP7435_Chr2-0909	77.0	2	3.8		Sorting nexin-4 Pichia pastoris
F2QUQ8_PICP7	PP7435_Chr3-0164	42.7	2	5.8		GTPase Pichia pastoris
F2QM85_PICP7	-	80.8	2	3.6		Hepatocyte growth factor-regulated tyrosine kinase substrate Pichia pastoris
C4R1Z8_PICPG	PAS_chr2-2_0383	123.6	2	2.2		Member of the imitation-switch (ISWI) class of ATP-dependent chromatin remodeling complexes Pichia pastoris
F2QQ40_PICP7	NOT4	48.0	2	7.5		CCR4-NOT transcription complex subunit 4 Pichia pastoris
F2QM54_PICP7	SCJ1	40.1	2	5.9		Chaperone protein dnaJ Pichia pastoris

F2QWB3_PICP7	HOM2	38.7	2	7.9		Aspartate-semialdehyde dehydrogenase Pichia pastoris
F2QNV0_PICP7	PP7435_Chr1-0542	36.6	2	8.4		Immunoglobulin A1 protease Pichia pastoris
F2QNC2_PICP7	YTM1	49.6	2	5.8		Uncharacterized WD repeat-containing protein alr3466 Pichia pastoris
F2QMW5_PICP7	DHH1	53.5	2	5.3		ATP-dependent RNA helicase DDX6/DHH1 Pichia pastoris
F2QR03_PICP7	STH1	142.8	2	2		ATP-dependent helicase STH1/SNF2 Pichia pastoris
F2QYF1_PICP7	ileS	123.4	2	2.1		Isoleucyl-tRNA synthetase Pichia pastoris
F2QSR0_PICP7	PP7435_Chr2-0753	19.5	2	17.2		Receptor expression-enhancing protein 5 Pichia pastoris
F2R015_PICP7	PP7435_Chr4-0840	22.9	2	11.7		E3 ubiquitin-protein ligase BRE1 Pichia pastoris
F2QS00_PICP7	ADA2	51.8	2	4.7		Transcriptional adapter 2 Pichia pastoris
F2QLH0_PICP7	PP7435_Chr1-1072	89.1	2	3		Midasin Pichia pastoris
F2QNY2_PICP7	Lsm7	17.7	2	14.1		U6 snRNA-associated Sm-like protein LSm7 Pichia pastoris
F2QYQ1_PICP7	MKT1	85.4	2	2.8		Protein MKT1 Pichia pastoris
F2QXS5_PICP7	PP7435_Chr4-0023	39.7	2	7.4		Putative uncharacterized protein Pichia pastoris
F2QUR7_PICP7	CYT1	31.9	2	10.1		Ubiquinol-cytochrome c reductase cytochrome c1 subunit Pichia pastoris
F2QRG4_PICP7	PP7435_Chr2-0298	34.5	2	7.2		37S ribosomal protein S24, mitochondrial Pichia pastoris
F2QXC7_PICP7	ERG1	55.1	2	4.7		Squalene monooxygenase Pichia pastoris
F2QV08_PICP7	PP7435_Chr3-0265	27.2	2	11.1		Peroxiredoxin (Alkyl hydroperoxide reductase subunit C) Pichia pastoris
C4QWD5_PICPG	PAS_chr1-1_0192	42.1	2	7.4		Mitochondrial ribosomal protein of the large subunit, has similarity to E. coli L2 ribosomal protein Pichia pastoris
F2QXE3_PICP7	PP7435_Chr3-1128	33.2	2	7.3		Uncharacterized protein C5D6.13 Pichia pastoris
F2QXM0_PICP7	PP7435_Chr3-1207	34.7	2	9		Ribosome biogenesis protein RLP7 Pichia pastoris
F2QRC4_PICP7	XPO1	123.8	2	2		Exportin-1 Pichia pastoris

F2QZU4_PICP7	PP7435_Chr4-0768	130.1	2	1.9		DNA-directed RNA polymerase Pichia pastoris
F2QZD2_PICP7	PP7435_Chr4-0600	18.5	2	12.7		Cell division control protein 31 Pichia pastoris
C4R126_PICPG	PAS_chr2-1_0863	59.1	2	4		Putative uncharacterized protein Pichia pastoris
C4R938_PICPG	PAS_chr4_0844	52.7	2	5.5		Protein disulfide isomerase, multifunctional protein resident in the endoplasmic reticulum lumen Pichia pastoris
F2QVD7_PICP7	PP7435_Chr3-0401	38.0	2	7.7		40S ribosomal protein MRP51, mitochondrial Pichia pastoris
F2QRF3_PICP7	GCD2	52.5	2	4.7		Translation initiation factor eIF-2B subunit delta Pichia pastoris
F2QSP1_PICP7	ARP3	47.8	2	4.9		Actin-related protein 3 Pichia pastoris
F2QMT5_PICP7	ADE4	59.9	2	4.2		Amidophosphoribosyltransferase Pichia pastoris
C4R5B2_PICPG	PAS_chr3_0699	28.4	2	10.1		Alpha 7 subunit of the 20S proteasome Pichia pastoris
F2QRW4_PICP7	PZF1	42.6	2	5.6		Zinc finger protein 167 Pichia pastoris
C4R444_PICPG	-	31.1	2	3.9		Protein component of the small (40S) ribosomal subunit Pichia pastoris
F2QZL1_PICP7	-	67.3	2	3.2		Putative uncharacterized protein Pichia pastoris
F2QQN7_PICP7	URA1	47.3	2	4.8		Dihydroorotate oxidase Pichia pastoris
F2QLS1_PICP7	PP7435_Chr1-0039	111.5	2	2.1		Vigilin Pichia pastoris
F2QV16_PICP7	THR1	38.3	2	5.6		Homoserine kinase Pichia pastoris
F2QQX3_PICP7	EBP2	41.4	2	5.5		rRNA-processing protein EBP2 Pichia pastoris
F2QP18_PICP7	RRP3	53.6	2	5.9		ATP-dependent RNA helicase Pichia pastoris
F2QTR9_PICP7	PP7435_Chr2-1120	49.8	2	2.5		Tubulin alpha chain Pichia pastoris
F2QWI9_PICP7	ERF2	78.2	2	3.1		Eukaryotic peptide chain release factor GTP-binding subunit Pichia pastoris
PARD3_HUMAN	-	109.9	1(3)	<4.9		Isoform 10 of Partitioning defective 3 homolog Homo sapiens
F2QYN7_PICP7	EMI2	52.4	1	4		Hexokinase Pichia pastoris
C4R0M8_PICPG	PAS_chr2-1_0429	13.9	1	22.3		Histone H2A Pichia pastoris
F2QNK9_PICP7	-	97.2	1	2.2		Cutinase transcription factor 1 alpha Pichia pastoris

F2QUR3_PICP7	TUP1	65.9	1	3.7		Transcriptional repressor TUP1 Pichia pastoris
F2QW05_PICP7	RRP4	38.4	1	6		Exosome complex component RRP4 Pichia pastoris
F2QN76_PICP7	22	93.2	1	2.8		Uncharacterized gene 22 protein Pichia pastoris
F2QMX1_PICP7	PP7435_Chr1-0786	16.0	1	13.1		Putative uncharacterized protein Pichia pastoris
F2QUJ1_PICP7	PP7435_Chr3-0097	91.2	1	2.3		Nuclear pore complex protein Nup107 Pichia pastoris
F2QMI1_PICP7	PP7435_Chr1-0476	7.8	1	30.6		DNA-directed RNA Polymerase II subunit K Pichia pastoris
F2QMA7_PICP7	TAF6	54.8	1	3.5		Transcription initiation factor TFIID subunit 6 Pichia pastoris
F2QPU3_PICP7	PP7435_Chr1-1302	43.8	1	4.1		Glucose repression protein GAL83 Pichia pastoris
F2QSC9_PICP7	GSL2	215.2	1	0.9		1,3-beta-glucan synthase Pichia pastoris
F2QMT9_PICP7	PP7435_Chr1-0752	46.0	1	4.8		Zinc finger protein 167 Pichia pastoris
F2QY75_PICP7	PP7435_Chr4-0178	31.1	1	6.1		Putative uncharacterized protein Pichia pastoris
C4R487_PICPG	PAS_chr3_0335	10.1	1	19.6		Protein component of the large (60S) ribosomal subunit, identical to Rpl43Ap Pichia pastoris
F2QXJ4_PICP7	PRS1	34.9	1	5		Ribose-phosphate pyrophosphokinase Pichia pastoris
F2QU14_PICP7	PP7435_Chr2-1217	49.1	1	4.1		Putative uncharacterized protein Pichia pastoris
F2QY50_PICP7	PP7435_Chr4-0153	106.3	1	1.6		Coatomer subunit beta Pichia pastoris
F2QLQ0_PICP7	PP7435_Chr1-1012	143.3	1	1.2		Putative uncharacterized protein Pichia pastoris
C4R2A0_PICPG	-	33.8	1	5.9		Putative uncharacterized protein Pichia pastoris
F2QPZ4_PICP7	PP7435_Chr1-1354	56.7	1	3.8		Exocyst complex component SEC3 Pichia pastoris
C4QYQ9_PICPG	-	56.0	1	3.7		Putative uncharacterized protein Pichia pastoris
F2QP89_PICP7	PP7435_Chr1-1087	45.0	1	3.8		Putative uncharacterized protein Pichia pastoris
F2QZR6_PICP7	PP7435_Chr4-0739	96.1	1	1.8		Importin subunit beta-1 Pichia pastoris
F2QUT3_PICP7	PP7435_Chr3-0189	38.9	1	5.2		Putative uncharacterized protein Pichia pastoris

F2QRF2_PICP7	PP7435_Chr2-0285	73.9	1	2.6		Serine/threonine-protein phosphatase 2A 56 kDa regulatory subunit delta isoform , B subunit, B' delta isoform Pichia pastoris
F2QN03_PICP7	PP7435_Chr1-0818	40.5	1	5.3		RNA-binding protein rnc1 Pichia pastoris
F2QXF6_PICP7	-	24.8	1	6.6		Polyadenylate-binding protein 2 Pichia pastoris
F2QWE5_PICP7	LSM1	17.8	1	10.2		Sm-like protein LSm1 Pichia pastoris
F2QMT3_PICP7	PP7435_Chr1-0746	26.1	1	7		Phosphoglycerate mutase Pichia pastoris
F2R030_PICP7	-	18.1	1	9.7		Coiled-coil domain-containing protein 117 Pichia pastoris
C4QWF0_PICPG	PAS_chr1-1_0205	13.3	1	12.1		Putative uncharacterized protein Pichia pastoris
C4R6F7_PICPG	PAS_chr3_1078	61.6	1	3.2		Subunit of the anaphase-promoting complex/cyclosome (APC/C) Pichia pastoris
F2QVL8_PICP7	MTR4	120.5	1	1.9		ATP-dependent RNA helicase DOB1 Pichia pastoris
F2QUU5_PICP7	FBP2	28.5	1	6.5		Agamous-like MADS-box protein AGL9 homolog Pichia pastoris
F2QUN8_PICP7	PWP1	64.9	1	2.4		Periodic tryptophan protein 1 Pichia pastoris
F2QTK7_PICP7	VMA5	44.1	1	4.1		V-type H+-transporting ATPase subunit C Pichia pastoris
F2QZ24_PICP7	CCR4	85.0	1	2.6		CCR4-NOT transcription complex subunit 6 Pichia pastoris
F2QQH0_PICP7	PP7435_Chr1-1537	16.8	1	12.2		Ubiquitin-conjugating enzyme E2 W Pichia pastoris
C4R2D9_PICPG	-	14.0	1	11.4		Putative uncharacterized protein Pichia pastoris
C4QY67_PICPG	PAS_chr1-4_0348	107.0	1	1.7		Putative uncharacterized protein Pichia pastoris
F2QYI0_PICP7	RGD1	70.6	1	2.2		Probable Rho-GTPase-activating protein 7 Pichia pastoris
C4R0F7_PICPG	PAS_chr2-1_0361	18.1	1	9.9		Subunit IV of cytochrome c oxidase Pichia pastoris
F2QRW5_PICP7	PP7435_Chr2-0455	17.8	1	10.3		DNA-directed RNA Polymerase II subunit F Pichia pastoris
F2QNX0_PICP7	COG4	93.7	1	2.2		Conserved oligomeric Golgi complex subunit 4 Pichia pastoris
AMPM2_PICPG	-	50.2	1	3.1		Methionine aminopeptidase 2 Pichia pastoris
NOP9_PICPG	-	74.3	1	2		Nucleolar protein 9 Pichia pastoris

F2QXL3_PICP7	PP7435_Chr3-1199	10.7	1	15.7		Uncharacterized protein YOR020W-A Pichia pastoris
F2QS25_PICP7	CMD1	16.8	1	10.7		Calmodulin Pichia pastoris
F2QQP2_PICP7	SOH1	21.8	1	8		Mediator of RNA polymerase II transcription subunit 31 Pichia pastoris
F2QXV4_PICP7	PP7435_Chr4-0052	23.0	1	6.8		Ras-related protein Rab-7A Pichia pastoris
F2QSK4_PICP7	PP7435_Chr2-0696	66.0	1	2.3		Uncharacterized RING finger protein P8B7.23 Pichia pastoris
F2QN01_PICP7	SBA1	22.5	1	7.5		Protein tyrosine phosphatase-like protein PTPLAD1 Pichia pastoris
F2QLF0_PICP7	PP7435_Chr1-1052	26.3	1	5.6		GrpE protein homolog, mitochondrial Pichia pastoris
F2QZ51_PICP7	CFA1	56.3	1	3.3		Uncharacterized methyltransferase in lpd-3 5'region Pichia pastoris
F2QSH4_PICP7	PP7435_Chr2-0665	61.7	1	2.4		Cortactin-binding protein 2 Pichia pastoris
F2R046_PICP7	-	182.0	1	0.8		Helicase SWR1 Pichia pastoris
C4QYZ9_PICPG	PAS_chr1-4_0614	11.2	1	17		Core Sm protein Sm D3 Pichia pastoris
F2QYC4_PICP7	PUF6	75.0	1	1.8		Protein PUF6 Pichia pastoris
F2QZR9_PICP7	PP7435_Chr4-0742	88.2	1	1.7		Putative uncharacterized protein Pichia pastoris
F2R0K4_PICP7	VAR1	43.8	1	3.7		Ribosomal protein VAR1, mitochondrial Pichia pastoris
F2QNV9_PICP7	PP7435_Chr1-0551	116.2	1	1.4		Putative SNF2 family helicase/ATPase Pichia pastoris
F2QX38_PICP7	ELC1	11.9	1	13.5		Transcription elongation factor B polypeptide 1 Pichia pastoris
F2QN99_PICP7	TKL1	73.9	1	2.2		Transketolase Pichia pastoris
C4QVV0_PICPG	-	48.5	1	3.6		Actin-like protein Pichia pastoris
F2QPP7_PICP7	-	26.0	1	7.3		Exosome complex component RRP40 Pichia pastoris
C4R294_PICPG	PAS_chr2-2_0291	9.9	1	12		Essential protein of the mitochondrial intermembrane space, forms a complex with Tim9p Pichia pastoris
F2QUI9_PICP7	PP7435_Chr3-0093	20.8	1	9.4		Exosome complex protein LRP1 Pichia pastoris
C4R837_PICPG	PAS_chr4_0506	28.0	1	6.3		Alpha 3 subunit of the 20S proteasome, the only nonessential 20S subunit Pichia pastoris

F2QSW6_PICP7	PRA1	19.6	1	7.4		Prenylated Rab acceptor protein 1 Pichia pastoris
F2QZX1_PICP7	PP7435_Chr4-0795	69.3	1	2.1		Putative uncharacterized protein Pichia pastoris
F2QSN8_PICP7	WHI2	36.0	1	3.8		Growth regulation protein Pichia pastoris
F2QWJ6_PICP7	GCD6	72.6	1	1.9		Translation initiation factor eIF-2B subunit epsilon Pichia pastoris
F2QQQ2_PICP7	PGP	32.3	1	6.1		4-nitrophenyl phosphatase Pichia pastoris
F2QLE1_PICP7	PP7435_Chr1-1043	92.7	1	1.7		Eukaryotic ribosome biogenesis protein 1 Pichia pastoris
C4R4P3_PICPG	PAS_chr3_0480	43.6	1	3.9		Putative chaperone, homolog of E. coli DnaJ, closely related to Ydj1p Pichia pastoris
F2QW06_PICP7	GUT2	74.2	1	2.1		Glycerol-3-phosphate dehydrogenase Pichia pastoris
C4R4V6_PICPG	PAS_chr3_0544	66.3	1	2.2		Component of the RSC chromatin remodeling complex Pichia pastoris
F2QPT7_PICP7	-	50.6	1	2.7		Nucleolar protein 12 Pichia pastoris
F2QNV4_PICP7	-	48.5	1	2.5		Isocitrate dehydrogenase [NADP] Pichia pastoris
F2QNA0_PICP7	LTP1	17.8	1	9.6		Protein-tyrosine phosphatase Pichia pastoris
F2QZ58_PICP7	PP7435_Chr4-0522	55.2	1	3.8		Protein SGT1 Pichia pastoris
F2QZT1_PICP7	PP7435_Chr4-0754	73.3	1	2.3		Putative uncharacterized protein Pichia pastoris
F2QZM2_PICP7	PP7435_Chr4-0693	23.5	1	7.7		Putative uncharacterized protein Pichia pastoris
F2QLL7_PICP7	PP7435_Chr1-0975	16.3	1	9.2		54S ribosomal protein L20, mitochondrial Pichia pastoris
F2QUY9_PICP7	-	94.9	1	1.4		Staphylococcal nuclease domain-containing protein 1 Pichia pastoris
F2QZC5_PICP7	PP7435_Chr4-0592	67.1	1	3.3		Nuclear RNA export factor 2 Pichia pastoris
F2QRJ4_PICP7	-	50.5	1	2.9		Uncharacterized WD repeat-containing protein alr3466 Pichia pastoris
F2QUM0_PICP7	PP7435_Chr3-0126	13.7	1	30.2		NHP2-like protein 1 homolog Pichia pastoris
F2QRP8_PICP7	NAF1	63.1	1	2.9		H/ACA ribonucleoprotein complex non-core subunit NAF1 Pichia pastoris
F2QU46_PICP7	PP7435_Chr2-1249	26.3	1	5.6		Glutaredoxin-3 Pichia pastoris
F2QVN9_PICP7	PP7435_Chr3-0507	35.8	1	4.5		Uncharacterized protein y4nL Pichia pastoris

F2QX66_PICP7	MAE1	69.4	1	2.5		Malic enzyme Pichia pastoris
F2QRA7_PICP7	COG8	47.5	1	3.6		Conserved oligomeric Golgi complex subunit 8 Pichia pastoris
F2QXF8_PICP7	-	67.9	1	2.1		Phenylpyruvate decarboxylase Pichia pastoris
F2QLY2_PICP7	TIF6	26.3	1	5.7		Eukaryotic translation initiation factor 6 Pichia pastoris
C4R059_PICPG	PAS_chr2-1_0821	53.1	1	2.6		Putative uncharacterized protein Pichia pastoris
F2QRB1_PICP7	PP7435_Chr2-0243	32.4	1	5		Transcription initiation factor TFIID subunit 11 Pichia pastoris
F2QP97_PICP7	SWI3	90.8	1	2		SWI/SNF complex subunit SMARCC2 Pichia pastoris
F2QT68_PICP7	PP7435_Chr2-0914	33.4	1	4.9		37S ribosomal protein S28, mitochondrial Pichia pastoris
F2QZA0_PICP7	-	90.8	1	1.7		Uncharacterized AAA domain-containing protein C16E9.10c Pichia pastoris
F2QLN0_PICP7	nip7	20.5	1	6.6		60S ribosome subunit biogenesis protein NIP7 Pichia pastoris
F2QUT5_PICP7	-	54.1	1	2.4		Protein WHI4 Pichia pastoris
F2QW75_PICP7	PP7435_Chr3-0697	16.6	1	11.9		Putative uncharacterized protein Pichia pastoris
F2QLQ8_PICP7	PP7435_Chr1-1020	13.4	1	10.2		60S ribosomal protein L34-A Pichia pastoris
F2QPG4_PICP7	PP7435_Chr1-1162	83.8	1	1.9		Protein-serine/threonine kinase Pichia pastoris
F2QW28_PICP7	PP7435_Chr3-0649	9.9	1	18.1		Putative uncharacterized protein Pichia pastoris
F2QZ84_PICP7	TIF35	31.7	1	4.2		Eukaryotic translation initiation factor 3 subunit G Pichia pastoris
F2QNA1_PICP7	NOT5	69.8	1	2.3		General negative regulator of transcription subunit 3 Pichia pastoris
F2QMZ0_PICP7	PP7435_Chr1-0805	31.0	1	4.3		Mitochondrial genome maintenance protein MGM101 Pichia pastoris
F2QMS6_PICP7	EHT1	51.1	1	2.4		Abhydrolase domain-containing protein 1 Pichia pastoris
F2QPE3_PICP7	PP7435_Chr1-1141	111.2	1	1.5		ATP-dependent RNA helicase Pichia pastoris
F2QSV6_PICP7	PP7435_Chr2-0801	21.4	1	8		Putative uncharacterized protein Pichia pastoris

F2QQ96_PICP7	-	51.7	1	3.3		Chromatin structure-remodeling complex protein RSC7 Pichia pastoris
F2QY67_PICP7	GLN1	41.4	1	4.9		Glutamine synthetase Pichia pastoris
F2QV59_PICP7	RIM1	14.2	1	11		Single-stranded DNA-binding protein Pichia pastoris
F2QRH8_PICP7	PDA1	44.0	1	3.5		Pyruvate dehydrogenase E1 component subunit alpha Pichia pastoris
F2QVA8_PICP7	-	61.1	1	2		ATP-dependent RNA helicase Pichia pastoris
F2QQ71_PICP7	PP7435_Chr1-1435	38.0	1	4.8		Cyclin-D1-binding protein 1 Pichia pastoris
F2QSD5_PICP7	PP7435_Chr2-0626	58.3	1	2.7		U3 small nucleolar RNA-associated protein 7 Pichia pastoris
F2QU62_PICP7	FES1	32.4	1	4.2		Hsp70 nucleotide exchange factor FES1 Pichia pastoris
F2QV31_PICP7	UTP5	67.2	1	2.5		WD repeat-containing protein 43 Pichia pastoris
C4QWC9_PICPG	PAS_chr1-1_0186	75.4	1	1.9		Putative GTPase involved in 60S ribosomal subunit biogenesis Pichia pastoris
F2QR53_PICP7	-	30.1	1	4.2		Cell division control protein 14 Pichia pastoris
C4R1M3_PICPG	PAS_chr2-1_0746	7.1	1	24.2		Putative uncharacterized protein Pichia pastoris
F2QTW6_PICP7	PP7435_Chr2-1168	33.0	1	4.9		37S ribosomal protein PET123, mitochondrial Pichia pastoris
F2QWH8_PICP7	CAR2	47.1	1	3.7		Ornithine--oxo-acid transaminase Pichia pastoris
F2QQ32_PICP7	PP7435_Chr1-1395	18.0	1	7.1		Putative uncharacterized protein Pichia pastoris
F2QW03_PICP7	PP7435_Chr3-0624	19.4	1	8.2		Mitochondrial import receptor subunit TOM20 Pichia pastoris
C4QWR3_PICPG	PAS_chr1-1_0309	63.2	1	2.6		Vacuolar ATPase V1 domain subunit A containing the catalytic nucleotide binding sites Pichia pastoris
F2R0E7_PICP7	PP7435_Chr4-0975	13.9	1	16.7		60S ribosomal protein L22-like 1 Pichia pastoris
C4R4X6_PICPG	PAS_chr3_0564	42.6	1	3.2		Beta subunit of the translation initiation factor eIF2B Pichia pastoris
F2QR43_PICP7	PP7435_Chr2-0174	49.9	1	3.2		Putative uncharacterized protein Pichia pastoris

F2QRX5_PICP7	PP7435_Chr2-0465	37.2	1	4.4		Probable DNA repair protein RAD23 Pichia pastoris
F2QVE4_PICP7	PP7435_Chr3-0410	88.2	1	1.5		Uncharacterized WD repeat-containing protein alr3466 Pichia pastoris
F2QZL2_PICP7	CLA4	84.9	1	1.7		Protein-serine/threonine kinase Pichia pastoris
F2QR05_PICP7	LCP5	39.2	1	4.4		Neuroguidin-A Pichia pastoris
C4R6A0_PICPG	PAS_chr3_1024	236.9	1	0.6		NAD(+)-dependent glutamate synthase (GOGAT) Pichia pastoris
F2R0A6_PICP7	PP7435_Chr4-0933	54.0	1	3		Histone acetyltransferase HTATIP Pichia pastoris
F2QVY9_PICP7	PP7435_Chr3-0609	9.7	1	14.9		40S ribosomal protein S21 Pichia pastoris
C4QYX1_PICPG	PAS_chr1-4_0691	56.9	1	2.1		Putative uncharacterized protein Pichia pastoris
F2QMV8_PICP7	-	43.4	1	3.5		60S ribosomal protein L2, mitochondrial Pichia pastoris
F2QP99_PICP7	PP7435_Chr1-1097	80.6	1	2		Protein SDA1 homolog Pichia pastoris
F2QW42_PICP7	PP7435_Chr3-0663	21.2	1	9		Uncharacterized protein YIL024C Pichia pastoris
F2QQ13_PICP7	PP7435_Chr1-1374	59.6	1	2.4		Hexose transporter 2 Pichia pastoris
C4QV58_PICPG	-	6.2	1	21.8		Putative uncharacterized protein Pichia pastoris
F2QYE4_PICP7	NOP7	66.7	1	2.2		Pescadillo homolog Pichia pastoris
F2QWW7_PICP7	MED4	32.0	1	3.9		Mediator of RNA polymerase II transcription subunit 4 Pichia pastoris
F2QRV2_PICP7	PP7435_Chr2-0441	58.3	1	2.3		Putative uncharacterized protein Pichia pastoris
F2QQU1_PICP7	CAP2	56.8	1	2.3		Adenylyl cyclase-associated protein Pichia pastoris
F2QX72_PICP7	SNF5	65.2	1	2.4		SWI/SNF chromatin-remodeling complex subunit SNF5 Pichia pastoris
F2QMX5_PICP7	RIA1	115.7	1	1.3		Elongation factor EF-2 Pichia pastoris
C4QZX5_PICPG	-	48.9	1	3.3		3-hydroxy-3-methylglutaryl-CoA (HMG-CoA) synthase, catalyzes the formation of HMG-CoA from acetyl-Co Pichia pastoris
F2QY03_PICP7	PP7435_Chr4-0105	36.3	1	4.1		Cell division control protein 10 Pichia pastoris
F2QV86_PICP7	PP7435_Chr3-0345	57.5	1	3.2		Eukaryotic translation initiation factor 2 subunit 3 Pichia pastoris

F2QZM8_PICP7	PP7435_Chr4-0699	8.9	1	14.9		26 proteasome complex subunit DSS1 Pichia pastoris
F2QTY5_PICP7	IDH2	40.0	1	3.3		Isocitrate dehydrogenase subunit 2, mitochondrial Pichia pastoris
F2QMU7_PICP7	PP7435_Chr1-0761	289.8	1	0.6		U3 small nucleolar RNA-associated protein 20 Pichia pastoris
F2QMV9_PICP7	PP7435_Chr1-0774	18.8	1	8		Molybdopterin-converting factor subunit 2 Pichia pastoris
F2QZN8_PICP7	PBP1	73.6	1	2.4		PAB1-binding protein 1 Pichia pastoris
F2QWA8_PICP7	PP7435_Chr3-0731	70.6	1	2.3		Anaphase-promoting complex subunit 4 Pichia pastoris
F2QN40_PICP7	-	45.5	1	3.2		Uncharacterized GTP-binding protein MJ1332 Pichia pastoris
F2QMA0_PICP7	RRS1	22.5	1	6.5		Regulator of ribosome biosynthesis Pichia pastoris
F2QPW7_PICP7	PP7435_Chr1-1326	17.0	1	8.5		Small nuclear ribonucleoprotein-associated protein B Pichia pastoris
F2QZD5_PICP7	GCD1	50.3	1	3.1		Bifunctional protein glmU Pichia pastoris
F2QSJ3_PICP7	PP7435_Chr2-0685	41.1	1	3.1		Putative uncharacterized protein Pichia pastoris
F2QRD7_PICP7	PP7435_Chr2-0270	13.5	1	10.2		30S ribosomal protein S10 Pichia pastoris
F2QNX3_PICP7	PP7435_Chr1-0565	40.7	1	3		Developmentally-regulated GTP-binding protein 1 Pichia pastoris
F2QT93_PICP7	-	40.3	1	3.8		Putative uncharacterized protein Pichia pastoris
F2QTM0_PICP7	PP7435_Chr2-1071	26.0	1	5.3		Nucleolar protein 16 Pichia pastoris
C4QW39_PICPG	PAS_chr1-1_0100	13.3	1	12.2		Mitochondrial ribosomal protein of the small subunit Pichia pastoris
F2R039_PICP7	PP7435_Chr4-0865	42.6	1	3		Cell surface glycoprotein 1 Pichia pastoris
F2QSL6_PICP7	ILV6	32.7	1	4		Acetolactate synthase I/III small subunit Pichia pastoris
C4R6F0_PICPG	PAS_chr3_1073	52.0	1	2.6		Peroxisomal membrane protein (PMP) Pichia pastoris
F2QNR8_PICP7	PP7435_Chr1-0342	65.1	1	2.1		Uncharacterized protein C11E10.04, mitochondrial Pichia pastoris
F2QLP0_PICP7	PP7435_Chr1-1002	54.2	1	3		Uncharacterized RNA-binding protein C23E6.01c Pichia pastoris

F2QRM7_PICP7	PP7435_Chr2-0361	34.6	1	3.5		26S proteasome non-ATPase regulatory subunit 14 Pichia pastoris
F2QZH5_PICP7	PP7435_Chr4-0645	15.9	1	7.6		30S ribosomal protein S12P Pichia pastoris
F2QRH0_PICP7	PP7435_Chr2-0304	28.3	1	5.5		Protein vip1 Pichia pastoris
F2QTI9_PICP7	pwp2	97.3	1	1.5		Periodic tryptophan protein 2 homolog Pichia pastoris
F2QXH7_PICP7	-	49.2	1	2.9		E3 ubiquitin-protein ligase BRE1 Pichia pastoris
F2QU35_PICP7	OLE1	56.0	1	2.4		Stearoyl-CoA desaturase (Delta-9 desaturase) Pichia pastoris
F2QWX2_PICP7	PP7435_Chr3-0954	44.9	1	3.4		Transcription initiation factor TFIID subunit 4 Pichia pastoris
F2QZC3_PICP7	PP7435_Chr4-0590	20.3	1	7.2		Putative uncharacterized protein Pichia pastoris
F2QUS6_PICP7	HBS1	86.3	1	2.2		Elongation factor EF-1 alpha subunit Pichia pastoris
F2QX28_PICP7	SIT4	36.1	1	3.2		Serine/threonine-protein phosphatase Pichia pastoris
F2QT99_PICP7	PP7435_Chr2-0946	87.8	1	1.4		E3 ubiquitin-protein ligase Pichia pastoris
F2QNJ0_PICP7	PP7435_Chr1-0262	24.4	1	7		Putative uncharacterized protein Pichia pastoris
F2QRW1_PICP7	QCR2	37.4	1	3.4		Ubiquinol-cytochrome c reductase core subunit 2 Pichia pastoris
F2QM33_PICP7	PP7435_Chr1-0155	31.6	1	4.7		Glucose repression protein GAL83 Pichia pastoris
F2QYZ8_PICP7	-	60.6	1	2.3		Halomucin Pichia pastoris
F2QQ10_PICP7	PRE4	29.7	1	4.2		20S proteasome subunit beta 7 Pichia pastoris
F2QUQ6_PICP7	-	32.8	1	4.4		Putative uncharacterized protein Pichia pastoris
F2QTU0_PICP7	PP7435_Chr2-1141	92.6	1	1.6		N-acetyl-gamma-glutamyl-phosphate reductase / acetylglutamate kinase Pichia pastoris
F2QRJ2_PICP7	PP7435_Chr2-0326	42.4	1	2.7		Protein FAM179B Pichia pastoris
F2QZ90_PICP7	PP7435_Chr4-0556	116.0	1	1.2		CCR4-NOT transcription complex subunit 7/8 Pichia pastoris
F2QWD8_PICP7	PP7435_Chr3-0762	39.2	1	4		Putative uncharacterized oxidoreductase YDR541C Pichia pastoris
F2QSU4_PICP7	PP7435_Chr2-0787	56.8	1	2.1		Putative uncharacterized protein Pichia pastoris

F2QQC6_PICP7	PP7435_Chr1-1491	177.6	1	0.8		Myosin-4 Pichia pastoris
F2QRV9_PICP7	PP7435_Chr2-0449	77.0	1	3.6		HDA1 complex subunit 3 Pichia pastoris
F2QRD5_PICP7	ada	19.2	1	7.1		Fused DNA-binding transcriptional dual regulator/O6-methylguanine-DNA methyltransferase Pichia pastoris
F2QQ55_PICP7	ARO8	54.1	1	2.7		Aromatic amino acid aminotransferase I Pichia pastoris
C4R872_PICPG	PAS_chr4_0537	56.9	1	2.9		Essential nuclear protein involved in early steps of ribosome biogenesis Pichia pastoris
F2QT73_PICP7	DBP8	48.2	1	2.3		ATP-dependent RNA helicase Pichia pastoris
F2QR37_PICP7	ADO1	37.9	1	3.4		Adenosine kinase Pichia pastoris
C4QY44_PICPG	-	41.0	1	2.9		Protein required for respiratory growth Pichia pastoris
F2QM04_PICP7	UGP1	56.4	1	3		UTP--glucose-1-phosphate uridylyltransferase Pichia pastoris
F2QYL7_PICP7	PP7435_Chr4-0325	18.5	1	8.8		54S ribosomal protein L25, mitochondrial Pichia pastoris
F2QZ91_PICP7	PP7435_Chr4-0557	27.2	1	5.4		Putative uncharacterized protein Pichia pastoris
F2QP61_PICP7	-	27.8	1	6.4		Essential for mitotic growth 1 Pichia pastoris
C4R6K3_PICPG	-	48.9	1	2.6		Subunit of tRNA (1-methyladenosine) methyltransferase with Gcd14p Pichia pastoris
F2QTN9_PICP7	-	156.0	1	0.8		SANT domain-containing protein 2 Pichia pastoris
F2QMU0_PICP7	PP7435_Chr1-0754	63.7	1	2.8		Ubiquitin carboxyl-terminal hydrolase Pichia pastoris
F2QQF3_PICP7	PP7435_Chr1-1520	86.8	1	1.4		GTPase-activating protein GYP5 Pichia pastoris
F2QR54_PICP7	GPM2	27.3	1	5		Phosphoglycerate mutase Pichia pastoris
F2QMM2_PICP7	-	27.0	1	5.5		mRNA turnover protein 4 homolog Pichia pastoris
F2QM73_PICP7	FOL2	28.7	1	4.8		GTP cyclohydrolase I Pichia pastoris
F2QT13_PICP7	PP7435_Chr2-0859	70.6	1	2.2		Uncharacterized RNA-binding protein YPL184C Pichia pastoris
C4QZS1_PICPG	-	10.2	1	18.2		Core Sm protein Sm E Pichia pastoris
F2R0D4_PICP7	PP7435_Chr4-0962	122.1	1	1.2		Nucleoporin nup120 Pichia pastoris
F2QV99_PICP7	GPM1	28.0	1	4.4		Phosphoglycerate mutase Pichia pastoris

C4R597_PICPG	PAS_chr3_0684	75.8	1	1.5		Protein that forms a nuclear complex with Noc2p that binds to 66S ribosomals Pichia pastoris
C4QY78_PICPG	PAS_chr1-4_0358	17.3	1	6.9		Protein associated with the mitochondrial nucleoid Pichia pastoris
F2QZJ5_PICP7	PP7435_Chr4-0665	48.2	1	2.7		O-acetylhomoserine (Thiol)-lyase Pichia pastoris
C4QVD7_PICPG	PAS_chr1-3_0150	208.0	1	0.6		Ubiquitin-protein ligase (E3) Pichia pastoris
C4R3V7_PICPG	PAS_chr3_0214	32.8	1	4.5		Cyclin-dependent kinase Pichia pastoris
F2QUV3_PICP7	TRP3	53.8	1	2.1		Anthranilate synthase / indole-3-glycerol phosphate synthase Pichia pastoris
F2QVW5_PICP7	PP7435_Chr3-0585	94.2	1	1.7		Ankyrin repeat domain-containing protein 50 Pichia pastoris
C4R7R0_PICPG	PAS_chr4_0387	46.9	1	2.6		One of six ATPases of the 19S regulatory particle of the 26S proteasome Pichia pastoris
F2QNX9_PICP7	PP7435_Chr1-0571	23.9	1	5		DNA-directed RNA polymerase III subunit C31 Pichia pastoris
F2QPP2_PICP7	KIN3	53.5	1	2.2		NIMA (Never in mitosis gene a)-related kinase Pichia pastoris
F2QLV3_PICP7	PDT1	41.9	1	2.9		Uncharacterized CDP-alcohol phosphatidyltransferase class-I family protein C22A12.08c Pichia pastoris
F2QQH7_PICP7	PFS2	50.0	1	3.2		Polyadenylation factor subunit 2 Pichia pastoris
F2QLP8_PICP7	GUF1	75.2	1	2.2		Translation factor GUF1, mitochondrial Pichia pastoris
F2QQ50_PICP7	PP7435_Chr1-1413	38.0	1	4.9		Sterol-4alpha-carboxylate 3-dehydrogenase (Decarboxylating) Pichia pastoris
F2QXV6_PICP7	PP7435_Chr4-0054	20.7	1	7		Uncharacterized protein YBL095W Pichia pastoris
F2QX09_PICP7	CRF1	57.2	1	2.3		DNA replication licensing factor MCM9 Pichia pastoris
F2QX40_PICP7	PP7435_Chr3-1022	44.6	1	2.6		Protein fyv10 Pichia pastoris
F2QMN2_PICP7	PP7435_Chr1-0694	85.5	1	1.6		Putative uncharacterized protein Pichia pastoris
F2QN26_PICP7	-	57.7	1	2.1		Putative uncharacterized protein Pichia pastoris
F2QTD2_PICP7	CAL1	63.0	1	2.1		Calnexin homolog 1 Pichia pastoris

F2QXG7_PICP7	-	105.2	1	1.5		Uncharacterized transcriptional regulatory protein C320.03 Pichia pastoris
C4R7Y3_PICPG	PAS_chr4_0455	13.2	1	8.4		Putative uncharacterized protein Pichia pastoris
F2QQW2_PICP7	PP7435_Chr2-0093	33.4	1	3.7		30S ribosomal protein S5, chloroplastic Pichia pastoris
F2QPU0_PICP7	-	55.8	1	2.6		Glutathione synthase Pichia pastoris
F2QTD5_PICP7	-	83.0	1	1.5		SWI5-dependent HO expression protein 4 Pichia pastoris
F2QS74_PICP7	PP7435_Chr2-0565	73.9	1	1.7		Uncharacterized protein YDL063C Pichia pastoris
F2QQE2_PICP7	PP7435_Chr1-1509	102.3	1	1.2		AP-3 complex subunit delta-1 Pichia pastoris
F2QSF8_PICP7	SAC6	70.2	1	2.1		Fimbrin Pichia pastoris
C4R7L9_PICPG	PAS_chr4_0350	5.7	1	28.6		Putative uncharacterized protein Pichia pastoris
C4R395_PICPG	-	53.7	1	2.1		Putative uncharacterized protein Pichia pastoris
ETT1_PICPG	ETT1	49.7	1	2.5		Enhancer of translation termination 1 Pichia pastoris
F2QSI0_PICP7	POL5	109.4	1	1.1		DNA polymerase phi subunit Pichia pastoris
C4R112_PICPG	PAS_chr2-1_0552	32.4	1	4.1		Protein involved in 20S proteasome assembly Pichia pastoris
F2QYK1_PICP7	PP7435_Chr4-0308	34.8	1	3.7		Protein MAK16 homolog A Pichia pastoris
F2R0F9_PICP7	PP7435_Chr4-0987	77.1	1	1.6		Putative uncharacterized protein Pichia pastoris
F2QNM6_PICP7	PRS2	46.6	1	2.8		Ribose-phosphate pyrophosphokinase Pichia pastoris
F2QXE2_PICP7	-	76.8	1	1.9		Putative uncharacterized protein Pichia pastoris
F2QVH3_PICP7	VAS1	118.7	1	1.1		Valyl-tRNA synthetase Pichia pastoris
C4R7E4_PICPG	PAS_chr4_0283	22.2	1	6.2		Putative uncharacterized protein Pichia pastoris
F2QXG6_PICP7	ade6	61.4	1	2.5		Phosphoribosylaminoimidazole carboxylase Pichia pastoris
F2QSK3_PICP7	CRN1	71.7	1	1.9		Coronin Pichia pastoris
F2QZJ7_PICP7	GCN1	303.0	1	0.4		Translational activator GCN1 Pichia pastoris
C4QVG0_PICPG	-	27.3	1	6		Putative uncharacterized protein Pichia pastoris
F2QQC0_PICP7	PP7435_Chr1-1485	22.7	1	6.5		Synaptobrevin homolog YKT6 Pichia pastoris
C4QYN4_PICPG	PAS_chr1-4_0504	6.7	1	17.9		40S ribosomal protein subunit Pichia pastoris
F2QVL3_PICP7	FET5	32.2	1	4.6		Transcription factor FET5 Pichia pastoris

F2QR07_PICP7	NDH	52.2	1	2.3		NADH-ubiquinone oxidoreductase 51 kDa subunit Pichia pastoris
F2QV27_PICP7	ahcy	48.7	1	2.7		Adenosylhomocysteinase Pichia pastoris
F2QP40_PICP7	PP7435_Chr1-0634	48.2	1	3		Homocitrate synthase Pichia pastoris
F2QVP2_PICP7	PP7435_Chr3-0510	33.2	1	3.4		Coatomer subunit epsilon-1 Pichia pastoris
F2QM18_PICP7	PP7435_Chr1-0139	37.0	1	3.5		Protein ATP11, mitochondrial Pichia pastoris
F2QQV3_PICP7	PP7435_Chr2-0084	33.7	1	5.9		Phosphatidylinositol transfer protein sfh5 Pichia pastoris
F2QQX8_PICP7	RPB4	20.6	1	6.5		DNA-directed RNA polymerase II subunit D Pichia pastoris
F2QVL9_PICP7	PP7435_Chr3-0487	16.3	1	6.8		Ubiquitin-conjugating enzyme E2 D/E Pichia pastoris
F2QW60_PICP7	-	103.3	1	1		Nucleoporin NUP1 Pichia pastoris
C4R5H5_PICPG	-	87.5	1	1.4		Carnitine acetyltransferase Pichia pastoris
F2QX35_PICP7	-	74.6	1	1.7		Nucleolar protein 4 Pichia pastoris
C4QZY3_PICPG	PAS_chr2-1_0199	34.7	1	3.9		Essential protein required for maturation of Gas1p and Pho8p, proposed to be involved in protein tra Pichia pastoris
F2QVW9_PICP7	SFB2	106.1	1	1.5		Protein transport protein SEC24 Pichia pastoris
F2R053_PICP7	RXT2	44.6	1	2.8		Transcriptional regulatory protein RXT2 Pichia pastoris
F2QSA8_PICP7	XPT1	23.3	1	5.4		Putative uncharacterized protein XPT1 Pichia pastoris
F2QS20_PICP7	CYC8	91.2	1	2.4		General transcriptional corepressor CYC8 Pichia pastoris
F2QRV1_PICP7	PP7435_Chr2-0439	31.9	1	3.9		54S ribosomal protein L10, mitochondrial Pichia pastoris
F2QQ15_PICP7	TRA1	437.6	1	0.3		Transcription-associated protein 1 Pichia pastoris
F2QT85_PICP7	LEUA	86.6	1	1.5		3-isopropylmalate dehydratase Pichia pastoris
F2QT54_PICP7	ASH2	39.8	1	3.2		Set1 complex component ash2 Pichia pastoris
F2QXC4_PICP7	PP7435_Chr3-1109	75.5	1	1.6		Non-specific serine/threonine protein kinase Pichia pastoris

C4QWQ3_PICPG	PAS_chr1-1_0299	51.1	1	2		Essential, non-ATPase regulatory subunit of the 26S proteasome lid Pichia pastoris
F2QZE8_PICP7	TFC7	51.6	1	2.4		Transcription factor tau 55 kDa subunit Pichia pastoris
F2QLC9_PICP7	PP7435_Chr1-1031	46.7	1	2.9		Homeobox-leucine zipper protein HOX33 Pichia pastoris
F2QZB7_PICP7	CKB1	28.4	1	5.7		Casein kinase II subunit beta Pichia pastoris
F2QZV1_PICP7	DBP4	87.2	1	2.2		RNA Helicase Pichia pastoris
F2QRA3_PICP7	PP7435_Chr2-0235	74.2	1	1.8		Internalin-I Pichia pastoris
F2QMH5_PICP7	PP7435_Chr1-0470	42.0	1	3		Protein disulfide isomerase family A,member 6 Pichia pastoris
F2QLI7_PICP7	PP7435_Chr1-0945	15.9	1	7.9		39S ribosomal protein L43, mitochondrial Pichia pastoris
F2QTK3_PICP7	PP7435_Chr2-1054	56.8	1	2.4		Putative uncharacterized protein Pichia pastoris
F2QS65_PICP7	-	91.3	1	1.5		Uncharacterized protein YKL050C Pichia pastoris
F2QSC8_PICP7	-	102.4	1	1.2		Nuclear cap-binding protein complex subunit 1 Pichia pastoris
F2QZA7_PICP7	PP7435_Chr4-0573	11.0	1	10.4		60S acidic ribosomal protein P1 Pichia pastoris
C4QZW4_PICPG	PAS_chr2-1_0179	25.6	1	4.9		S-adenosylmethionine-dependent methyltransferase of the seven beta-strand family Pichia pastoris
F2QQX4_PICP7	EFG5	106.5	1	1.4		116 kDa U5 small nuclear ribonucleoprotein component Pichia pastoris
F2QVA2_PICP7	COX6	16.7	1	6.2		Cytochrome c oxidase subunit Va Pichia pastoris
F2QZM7_PICP7	pam2	46.8	1	2.8		26S protease regulatory subunit 6A Pichia pastoris
F2QNK7_PICP7	SPB4	69.9	1	1.8		One of two almost identical potential DEAD box RNA helicase genes similar to S. cerevisiae SPB4 (YFL002C) ATP-dependent RNA helicase involved in the maturation of 25S ribosomal RNA Pichia pastoris
F2QMT7_PICP7	ILV5	44.3	1	2.8		Ketol-acid reductoisomerase Pichia pastoris
F2QQ41_PICP7	PP7435_Chr1-1404	121.4	1	1.2		Importin subunit beta-5 Pichia pastoris

F2QV19_PICP7	-	51.4	1	2.4		Putative uncharacterized protein Pichia pastoris
F2QYM6_PICP7	PP7435_Chr4-0334	80.9	1	1.9		Regulatory protein SWI6 Pichia pastoris
F2QUC4_PICP7	-	163.9	1	0.8		Mediator of RNA polymerase II transcription subunit 13 Pichia pastoris
MZM1_PICPG	-	13.5	1	8.2		Mitochondrial zinc maintenance protein 1, mitochondrial Pichia pastoris
F2QS52_PICP7	PP7435_Chr2-0543	16.1	1	7.6		Calmodulin-like protein 4 Pichia pastoris
F2QV91_PICP7	hob3	32.6	1	3.6		Probable guanine nucleotide exchange factor FLJ41603 homolog Pichia pastoris
C4QWH0_PICPG	PAS_chr1-1_0224	35.7	1	3.4		Nuclear pore protein that is part of the evolutionarily conserved Nup84p complex Pichia pastoris
F2QUJ5_PICP7	PP7435_Chr3-0101	31.8	1	4.5		Polyribonucleotide nucleotidyltransferase Pichia pastoris
F2QQP5_PICP7	-	33.5	1	3.4		U1 small nuclear ribonucleoprotein 70 kDa Pichia pastoris
F2QZ68_PICP7	Lsm4	11.6	1	11.8		U6 snRNA-associated Sm-like protein LSm4 Pichia pastoris
LIPA_PICPG	-	46.5	1	2.6		Lipoyl synthase, mitochondrial Pichia pastoris
F2QSJ0_PICP7	PP7435_Chr2-0682	153.3	1	0.8		Putative ATP-dependent RNA helicase YLR419W Pichia pastoris
F2QS87_PICP7	ACT2	57.8	1	2.1		Acyl-coenzyme A thioesterase 9, mitochondrial Pichia pastoris
F2QUI2_PICP7	PP7435_Chr3-0086	280.9	1	0.5		Pre-mRNA-processing-splicing factor 8 Pichia pastoris
F2QVH9_PICP7	PP7435_Chr3-0445	23.3	1	5.1		20S proteasome subunit beta 1 Pichia pastoris
F2QUX3_PICP7	PP7435_Chr3-0229	37.3	1	3.1		Coproporphyrinogen III oxidase Pichia pastoris
F2QNP3_PICP7	RRP1	43.0	1	2.7		Ribosomal RNA processing protein 1 homolog B Pichia pastoris
F2QLI5_PICP7	ARP4	52.9	1	2.6		Beta-actin-like protein 2 Pichia pastoris
F2QZ71_PICP7	-	81.6	1	1.7		Putative uncharacterized protein Pichia pastoris
F2QRY5_PICP7	-	141.8	1	0.9		Uncharacterized protein YHR155W Pichia pastoris

F2QUT9_PICP7	GCS1	40.0	1	3.4		ADP-ribosylation factor GTPase-activating protein GCS1 Pichia pastoris
F2QSR1_PICP7	APL4	89.2	1	1.4		AP-1 complex subunit gamma-1 Pichia pastoris
F2R0E5_PICP7	PP7435_Chr4-0973	18.6	1	6.2		Putative uncharacterized protein Pichia pastoris
F2QT25_PICP7	HTB2	14.4	1	8.3		Histone H2B Pichia pastoris
F2QRX2_PICP7	PP7435_Chr2-0462	47.2	1	2.7		Putative uncharacterized protein Pichia pastoris
F2QMW8_PICP7	APL2	75.9	1	1.6		AP-2 complex subunit beta-1 Pichia pastoris
F2QLD0_PICP7	MET3	61.2	1	2		Sulfate adenylyltransferase Pichia pastoris
F2QTE5_PICP7	-	57.7	1	2		Glucose-6-phosphate 1-dehydrogenase Pichia pastoris
C4R0T9_PICPG	-	6.2	1	19.6		Putative uncharacterized protein Pichia pastoris
F2QTP5_PICP7	-	51.7	1	2.3		Beclin-1-like protein Pichia pastoris
F2QQS9_PICP7	PP7435_Chr2-0060	68.1	1	2.3		Eukaryotic translation initiation factor 2A Pichia pastoris
F2QY44_PICP7	RPF1	34.8	1	3.4		Brix domain-containing protein F44G4.1 Pichia pastoris
F2QWK4_PICP7	SKI6	27.6	1	4.1		Exosome complex component RRP41 Pichia pastoris
C4R4T3_PICPG	PAS_chr3_0520	13.6	1	9.1		Putative mitochondrial ribosomal protein of the small subunit Pichia pastoris
C4R2X4_PICPG	PAS_chr2-2_0081	53.0	1	2.6		Constituent of the mitochondrial inner membrane presequence translocase (TIM23 complex) Pichia pastoris
F2QXA7_PICP7	-	66.1	1	2.9		Phenylalanyl-tRNA synthetase beta chain Pichia pastoris
F2QMY9_PICP7	MYH2	176.8	1	0.8		Myosin-2 , skeletal muscle, adult 2 Pichia pastoris
F2QR30_PICP7	SEN2	44.3	1	3.2		tRNA-intron endonuclease Pichia pastoris
F2QT03_PICP7	-	129.5	1	0.7		Putative DNA helicase ino80 Pichia pastoris
F2QRW7_PICP7	SKI3	158.9	1	0.8		Superkiller protein 3 Pichia pastoris
F2QNF9_PICP7	PHR2	58.3	1	2.1		pH-responsive protein 2 Pichia pastoris
F2QTL2_PICP7	PP7435_Chr2-1063	48.7	1	2.1		Protein ECM18 Pichia pastoris

F2QSH1_PICP7	PP7435_Chr2-0662	16.4	1	7.5		54S ribosomal protein L27, mitochondrial Pichia pastoris
F2QMB1_PICP7	RSM7	28.1	1	4.9		30S ribosomal protein S7 Pichia pastoris
F2QTS7_PICP7	CFT2	95.4	1	1.5		Cleavage and polyadenylation specificity factor subunit 2 Pichia pastoris
F2QMD2_PICP7	CIT1	51.9	1	2.6		Citrate synthase Pichia pastoris
F2QRM3_PICP7	TAL1	35.5	1	4		Transaldolase Pichia pastoris
F2QWD0_PICP7	PGI1	62.0	1	1.8		Glucose-6-phosphate isomerase Pichia pastoris
F2QXV1_PICP7	PAF1	38.5	1	3		RNA polymerase II-associated protein 1 homolog Pichia pastoris
F2QS95_PICP7	RIX1	82.7	1	1.8		Pre-rRNA-processing protein RIX1 Pichia pastoris
F2QLW8_PICP7	ARD1	23.5	1	5.4		N-terminal acetyltransferase complex ARD1 subunit Pichia pastoris
F2QVU0_PICP7	PTA1	85.4	1	1.6		mRNA cleavage and polyadenylation specificity factor complex subunit pta1 Pichia pastoris
F2QUF9_PICP7	PP7435_Chr3-0063	57.9	1	3.7		Putative uncharacterized protein Pichia pastoris
F2QXV2_PICP7	-	29.5	1	4.2		Pleckstrin homology-like domain family B member 1 Pichia pastoris
F2QPH3_PICP7	PP7435_Chr1-1172	30.2	1	4.2		Protein hob1 Pichia pastoris
F2QVW6_PICP7	-	28.0	1	4.3		Uncharacterized protein YNL050C Pichia pastoris
F2QYC5_PICP7	SDH1	70.2	1	1.6		Succinate dehydrogenase (Ubiquinone) flavoprotein subunit Pichia pastoris
F2QZP5_PICP7	-	27.6	1	3.6		Electron transfer flavoprotein subunit beta Pichia pastoris
F2QQD6_PICP7	ENT3	46.7	1	3.3		ENTH domain-containing protein C794.11c Pichia pastoris
F2QNB2_PICP7	PP7435_Chr1-0932	11.2	1	10.6		10 kDa chaperonin Pichia pastoris
C4QVU4_PICPG	-	9.4	1	11.9		Lsm (Like Sm) protein Pichia pastoris
F2QNT0_PICP7	ATP7	19.7	1	5.1		F-type H+-transporting ATPase subunit d Pichia pastoris
F2QMW6_PICP7	PP7435_Chr1-0781	47.8	1	2.8		Mitogen-activated protein kinase kinase Pichia pastoris

C4R075_PICPG	PAS_chr2-1_0285	91.3	1	1.2		Mitochondrial integral membrane protein involved in mitochondrial fusion and maintenanceof the mitoc Pichia pastoris
F2QX20_PICP7	PP7435_Chr3-1002	94.2	1	1.2		DNA repair and recombination protein RAD54 and RAD54-like protein Pichia pastoris
F2QSQ4_PICP7	-	45.8	1	3.3		Putative uncharacterized protein Pichia pastoris
F2QR02_PICP7	CCP1	42.0	1	3.2		Cytochrome c peroxidase Pichia pastoris
C4R9G0_PICPG	PAS_c034_0038	31.7	1	3.3		26S proteasome non-ATPase regulatory particle subunit Pichia pastoris
F2QLK4_PICP7	PP7435_Chr1-0962	54.8	1	2.4		Pre-mRNA-processing protein prp40 Pichia pastoris
F2QW37_PICP7	CAP2	31.5	1	3.6		F-actin-capping protein subunit beta Pichia pastoris
F2QVQ5_PICP7	PP7435_Chr3-0523	59.7	1	2.7		UPF0662 protein C30C2.08 Pichia pastoris
F2QUM6_PICP7	CTR9	120.1	1	1.2		RNA polymerase-associated protein CTR9 homolog Pichia pastoris
C4R3P3_PICPG	PAS_chr3_0150	22.0	1	7		GTP-binding protein of the rho subfamily of Ras-like proteins, involved in establishment of cell pol Pichia pastoris
F2QP31_PICP7	RBS1	39.3	1	3		R3H domain-containing protein 2 Pichia pastoris
C4R9C7_PICPG	PAS_FragD_0003	61.5	1	1.7		Subunit of the CCR4-NOT complex Pichia pastoris
C4QVU8_PICPG	PAS_chr1-1_0018	60.0	1	1.9		Subunit of the heterodimeric FACT complex (Spt16p-Pob3p) Pichia pastoris
C4R329_PICPG	-	16.8	1	7.2		Phosphorelay intermediate protein Pichia pastoris
C4R816_PICPG	PAS_chr4_0484	98.6	1	1.6		Member of the Sec24p family Pichia pastoris
F2QWZ5_PICP7	pst1	161.9	1	0.7		Transcriptional regulatory protein SIN3 Pichia pastoris

End of list.
